# Methoxylated Cinnamic Esters with Antiproliferative and Antimetastatic Effects on Human Lung Adenocarcinoma Cells

**DOI:** 10.3390/life13071428

**Published:** 2023-06-22

**Authors:** João Graciano Sampaio, Carolina Girotto Pressete, Adilson Vidal Costa, Felipe Terra Martins, Graziela Domingues de Almeida Lima, Marisa Ionta, Róbson Ricardo Teixeira

**Affiliations:** 1 Grupo de Síntese e Pesquisa de Compostos Bioativos (GSPCB), Departamento de Química, Universidade Federal de Viçosa, Viçosa 36570-900, MG, Brazil; joaogracianojg@hotmail.com; 2 Programa de Pós-Graduação em Biociências Aplicadas à Saúde, Instituto de Ciências Biomédicas, Universidade Federal de Alfenas, Alfenas 37130-000, MG, Brazil; carolina.pressete@sou.unifal-mg.edu.br (C.G.P.); graziela.lima@unifal-mg.edu.br (G.D.d.A.L.); 3 Departamento de Química e Física, Universidade Federal do Espírito Santo, Guararema, Alegre 29500-000, ES, Brazil; avcosta@hotmail.com; 4 Departamento de Química, Universidade Federal de Goiás, Goiânia 74690-900, GO, Brazil; felipe@ufg.br

**Keywords:** non-small-cell lung cancer, monomethoxylated cinnamic acid derivatives, antiproliferative activity

## Abstract

Lung cancer is the leading cause of cancer mortality worldwide, and malignant melanomas are highly lethal owing to their elevated metastatic potential. Despite improvements in therapeutic approaches, cancer treatments are not completely effective. Thus, new drug candidates are continuously sought. We synthesized mono- and di-methoxylated cinnamic acid esters and investigated their antitumor potential. A cell viability assay was performed to identify promising substances against A549 (non-small-cell lung cancer) and SK-MEL-147 (melanoma) cells. (*E*)-2,5-dimethoxybenzyl 3-(4-methoxyphenyl)acrylate (**4m**), a monomethoxylated cinnamic acid derivative, was identified as the lead antitumor compound, and its antitumor potential was deeply investigated. Various approaches were employed to investigate the antiproliferative (clonogenic assay and cell cycle analysis), proapoptotic (annexin V assay), and antimigratory (wound-healing and adhesion assays) activities of **4m** on A549 cells. In addition, western blotting was performed to explore its mechanism of action. We demonstrated that **4m** inhibits the proliferation of A549 by promoting cyclin B downregulation and cell cycle arrest at G2/M. Antimigratory and proapoptotic activities of **4m** on A549 were also observed. The antitumor potential of **4m** involved its ability to modulate the mitogen-activated protein kinases/extracellular signal-regulated kinase (MAPK/ERK) signaling pathway once phosphorylated-ERK expression was considerably reduced in response to treatment. Our findings demonstrate that **4m** is a promising anticancer drug candidate.

## 1. Introduction

Cancer is one of the leading causes of human deaths worldwide, and thus remains a public health problem [1,2]. It is projected that with the growth and aging of the population, the global burden will reach 30.3 million new cancer cases and 16.3 million cancer deaths by 2040 [3].

Cancer treatment involves different therapeutic approaches, which depend on the type and stage of cancer [4]. Systemic treatment is widely used [5]; however, it causes severe side effects in patients [6]. Besides this, drug resistance acquisition in cancer cells is a crucial problem in cancer therapy [7,8]. Thus, the research for and development of new drugs that overcome the aforementioned problems are relevant.

**Figure 1:**
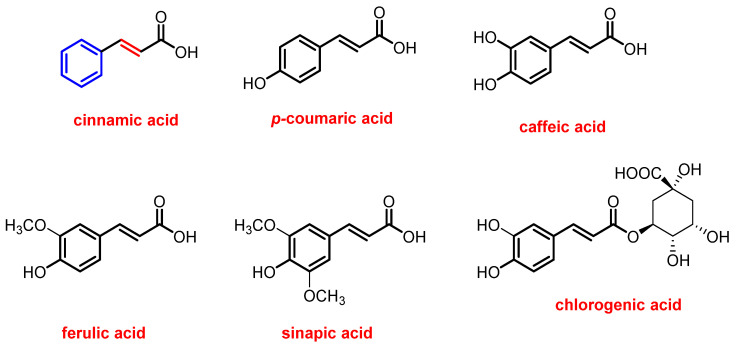
Structures of cinnamic acid and its derivatives found in nature.

The natural product pool is a valuable source of bioactive compounds to be explored in the research, and used in the development of new cancer drugs [9,10,11,12,13]. Cinnamic acid is an α,β-unsaturated (shown in red in Figure 1) carboxylic acid bearing a phenyl ring (shown in blue in Figure 1). This compound and its derivatives (some are also depicted in Figure 1) are abundantly found in plant-based foods such as fruits, vegetables, and whole grains [14]. Cinnamic acid can be generally obtained from cinnamon (*Cinnamomum cassia* (L.) Kuntze), cocoa (*Theobroma cacao* L.), spinach (*Spinacia oleracea* L.), celery (*Apium graveolens* L.), and brassicas vegetables. Other sources of cinnamic acid and derivatives include cereal grains, coffee (the primary source of caffeic acid), rice, wheat bran, sweet potatoes (*Ipomoeab batatas* L.), Chinese propolis, Cimicifuga (*Cimicifuga heracelifolia* var. *bifida* Nakai), peanuts (*Arachis hypogaea* L.), basil (*Ocimum basilicum* L.), garlic (*Allium sativum* L.), Granny’s Nightcap (*Aquilegia vulgaris* L.), Bueger’s Figwort (*Scrophylaria buergeriana* Miq.), and medicinal herbs in oriental countries such as China and Japan [15]. These natural compounds display pharmacological potential owing to their antimicrobial, antioxidant, antiprotozoal, neuroprotective, antidiabetic, and anticancer activities [14,15,16,17,18].

The antitumor potential of cinnamic acid and its derivatives [19,20,21] has been demonstrated. Niero et al. (2013) demonstrated cinnamic acid-induced apoptosis in HTT-144 human melanoma cells, enacted by disrupting the cytoskeleton [19]. Yen et al. (2011) reported that both *cis*- and *trans*-cinnamic acids could inhibit the phorbol-12-myristate-13-acetate-stimulated invasive behavior of lung adenocarcinoma A549 cells [20]. Tsai et al. (2013) demonstrated that cinnamic acid derivatives, such as caffeic acid, ferulic acid, and chlorogenic acid, inhibited the invasive behavior of A549 cells by modulating oncogenic signaling pathways, including mitogen-activated protein kinases/extracellular signal-regulated kinase (MAPK/ERK) and phosphoinositide-3-kinase/protein kinase [21].

Cutaneous melanoma is an aggressive skin cancer with high mortality rates [1,22,23]. Classical chemotherapy, and immune and targeted therapies, are generally used for metastatic melanoma. Although these treatments have improved the clinical outcome of many patients, there are still either refractory cases or those exhibiting side effects [24,25,26,27,28].

Lung cancer is the most common cause of cancer-related death in men and the second most common in women [29]. Among the diagnosed cases, ~85% are classified as non-small-cell lung cancer (NSCLC), a group of histological subtypes in which lung adenocarcinoma is the most prevalent (~40% of cases) [30,31,32]. Systemic treatment for NSCLC uses different substances, including platinum-based drugs, molecular-targeted agents, and immune checkpoint agents [33,34,35,36,37,38]. Although therapeutic improvements have been achieved by introducing targeted immunotherapy, the mortality rate for lung cancer remains high.

Based on the exposed scenario, it is imperative to advance the engineering of new chemical structures that can serve as starting structures for developing new chemotherapeutic agents, especially for cancers that are aggressive and resistant to available drugs.

We have been interested in synthesizing and biologically investigating compounds inspired by natural products that may have a promising antitumor effect against solid tumors, such as melanoma and lung adenocarcinoma [39,40,41,42]. Thus, herein, we describe the preparation of methoxylated cinnamic acid esters and cinnamides, and their cytotoxic profile against melanoma SK-MEL-147 and lung adenocarcinoma A549 cells. In addition, the antiproliferative and antimetastatic activities and proapoptotic effects of lead compound **4m** on A549 cells were investigated.

## 2. Materials and Methods

### 2.1. Synthesis

#### 2.1.1. Generalities

Solvents, sodium chloride, and sodium sulfate were purchased from F Maia (Charqueada, São Paulo State, Brazil). Commercially available 4-methoxy cinnamic acid, 3,4-dimethoxy cinnamic acid, *N,N*′-diisopropylcarbodiimide (DIC), 4-*N,N*-dimethylaminopyridine (DMAP), benzyl alcohol, 4-isopropylbenzyl alcohol, 4-methoxybenzyl alcohol, 4-trifluoromethoxybenzyl alcohol, 4-nitrobenzyl alcohol, 4-chlorobenzyl alcohol, 4-bromobenzyl alcohol, 4-fluorobenzyl alcohol, 3,4-difluorobenzyl alcohol, 2-bromobenzyl alcohol, perylic alcohol, 4-hydroxymethyl pyridine, 2,5-dimethoxybenzyl alcohol, eugenol, and guaiacol were purchased from Sigma Aldrich (St. Louis, MO, USA) and used without further purification. The nuclear magnetic resonance (NMR) spectra were recorded on a Varian Mercury 300 instrument (Varian, Palo Alto, CA, USA) at 300 MHz (^1^H) and 75 MHz (^13^C). Deuterated chloroform (CDCl_3_) was used as a solvent to acquire the NMR spectra. The ^1^H NMR data are presented as follows: chemical shift (*δ*) in ppm, multiplicity, number of protons, and *J* values in Hz. Multiplicities are indicated by the following abbreviations: s (singlet), d (doublet), m (multiplet), sept (septet), brs (broad singlet), d (doublet), ddtap (apparent doublet of doublet of triplets). For fluorine-containing derivatives, the multiplicity (quartet, q) and *J* values are described in Hz. IR spectra were obtained using Varian 660-IR equipped with GladiATR (Varian, Palo Alto, CA, USA), scanning from 4000 to 500 cm^−1^. Mass spectra were obtained with a GC-MS QP Plus 2010 from Shimadzu (Kyoto, Japan) using the electron ionization mode of 70 eV. A capillary column of fused silica Rtx-5MS (30 m long, 0.25 mm internal diameter) was used with helium as the carrier gas. The temperatures were 220 °C for the injector and 300 °C for the detector. The initial column temperature was 60 °C, programmed for an increase of 3 °C per minute until reaching the maximum temperature of 240 °C. Melting points were determined using an MQAPF-302 melting point apparatus (Microquímica, Palhoça, Santa Catarina State, Brazil) and are uncorrected. The progress of the reactions was monitored using thin-layer chromatography (TLC). After analysis, TLC was revealed using UV light and potassium permanganate solution. Flash column chromatography was performed using silica gel (60–230 mesh).

#### 2.1.2. Preparation of Compounds **4a**–**4o** Exemplified by Using the Obtention of (*E*)-Benzyl 3-(4-Methoxyphenyl)acrylate (**4a**)

A 50 mL round bottom flask was charged with benzyl alcohol (0.130 g, 1.20 mmol), dichloromethane (10 mL), 4-methoxy cinnamic acid (**1**) (0.195 g, 1.09 mmol), DIC (0.138 g, 1.09 mmol), and DMAP (0.0134 g, 0.110 mmol). The resulting mixture was stirred for 60 min at room temperature. After completion of the reaction, as confirmed by TLC analysis, the mixture was filtered and the organic layer was washed with distilled water (15.0 mL) and saturated sodium chloride solution (30.0 mL). The organic phase was reserved and the aqueous solution was extracted with dichloromethane (3 × 20.0 mL). The organic phases were combined and the resulting organic layer was dried over sodium sulfate, filtered, and concentrated under reduced pressure. The residue was submitted to purification using silica gel column chromatography, eluting with hexane/ethyl acetate (4:1 *v*/*v*). This procedure afforded compound **4a** with 64% yield (0.188 g, 0.701 mmol). The following data support the structure of **4a**.

Yellow solid, m.p. 47.9***–***48.8 °C. TLC R_f_ = 0.36 (hexane/ethyl acetate 4:1 *v*/*v*). IR (ATR) ${\overset{-}{v}}_{max}$(cm^−1^): 3031, 2955, 2834, 2158, 1974, 1702, 1630, 1600, 1573, 1509, 1458, 1419, 1376, 1286, 1249, 1201, 1150, 1029, 984, 902, 827, 742, 691, 600, 549, 510. ^1^H NMR (300 MHz, CDCl_3_) *δ*: 3.80 (s, 3H), 5.22 (s, 2H), 6.33 (d, 1H, *J_trans_* = 16.0 Hz), 6.87 (d, 2H, *J* = 8.7 Hz), 7.30***–***7.39 (m, 5H), 7.44 (d, 2H, *J* = 8.7 Hz), 7.66 (d, 1H, *J_trans_* = 16.0 Hz). ^13^C NMR (75 MHz, CDCl_3_) *δ*: 50.6, 61.4, 109.6, 110.6, 122.4, 123.4, 123.5, 123.8, 125.0, 131.5, 140.1, 156.7, 162.4. MS, *m/z* (%): 268, C_17_H_16_O_3_, [M^+^], (62.7); 223 (44.9); 161 (62.0); 134 (66.5); 91 (100).

The cinnamic acid derivatives **4b–4o** were obtained utilizing the procedure described above, and their structures are supported by the following data.

(*E*)-4-isopropylbenzyl 3-(4-methoxyphenyl)acrylate (**4b**)

White solid, obtained in 70% yield, purified by silica gel column chromatography, eluting with hexane/ethyl acetate (10:1 *v*/*v*), m.p. 75.2***–***75.4 °C. TLC R_f_ = 0.19 (hexane/ethyl acetate 10:1 *v*/*v*). IR (ATR) ${\overset{-}{v}}_{max}$(cm^−1^): 3028, 2952, 2834, 2161, 1699, 1630, 1600, 1573, 1509, 1452, 1422, 1376, 1286, 1246, 1156, 1032, 999, 969, 917, 827, 760, 700, 634, 591, 516. ^1^H NMR (300 MHz, CDCl_3_) *δ*: 1.26 (d, 6H, *J* = 6.9 Hz), 2.92 (sept, 1H, *J* = 6.9 Hz), 3.83 (s, 3H), 5.21 (s, 2H), 6.35 (d, 1H, *J_trans_* = 16.0 Hz), 6.90 (d, 2H, *J* = 8.7 Hz), 7.25 (d, 2H, *J* = 8.1 Hz), 7.35 (d, 2H, *J* = 8.1 Hz), 7.47 (d, 2H, *J* = 8.7 Hz), 7.68 (d, 1H, *J_trans_* = 16.0 Hz). ^13^C NMR (75 MHz, CDCl_3_) *δ*: 23.9, 33.9, 55.4, 66.2, 114.3, 115.5, 126.7, 127.1. 128.5, 129.7, 144.7, 149.0, 161.4, 167.2. MS, *m/z* (%): 310, C_20_H_22_O_3_, [M^+^], (35.4); 267 (38.6); 223 (25.2); 161 (51.2); 133 (69.6); 117 (24.2); 105 (34.7); 91 (25.0); 77 (12.4).

(*E*)-4-methoxybenzyl 3-(4-methoxyphenyl)acrylate (**4c**)

White solid, obtained in 73% yield, purified by silica gel column chromatography, eluting with hexane/ethyl acetate (3:2 *v*/*v*), m.p. 66.2***–***66.8 °C. TLC R_f_ = 0.31 (hexane/ethyl acetate 3:2 *v*/*v*). IR (ATR) ${\overset{-}{v}}_{max}$ (cm^−1^): 3010, 2961, 2925, 2828, 2364, 2164, 1696, 1633, 1600, 1573, 1512, 1461, 1422, 1289, 1243, 1159, 1023, 978, 824, 564, 519. ^1^H NMR (300 MHz, CDCl_3_) *δ*: 4.46***–***4.51 (m, 6H), 5.85 (s, 2H), 7.00 (d, 1H, *J_trans_* = 16.4 Hz), 7.50***–***7.61 (m, 4H), 8.02 (d, 2H, *J* = 8.7 Hz), 8.13 (d, 2H, *J* = 7.2 Hz), 8.33 (d, 1H, *J_trans_* = 16.4 Hz). ^13^C NMR (75 MHz, CDCl_3_) *δ*: 56.0, 66.7, 114.6, 115.0, 116.1, 127.8, 129.0, 130.4, 130.8, 145.3, 160.3, 162.1, 167.9. MS, *m/z* (%): 298, C_18_H_18_O_4_, [M^+^], (14.4); 253 (23.0); 121 (100); 77 (15.1).

(*E*)-4-(trifluoromethoxy)benzyl 3-(4-methoxyphenyl)acrylate (**4d**)

White solid, obtained at 82%, purified by silica gel column chromatography, eluting with hexane/ethyl acetate (*v*/*v*), m.p. 58.4***–***59.2 °C. TLC R_f_ = 0.50 (hexane/ethyl acetate 3:2 *v*/*v*). IR (ATR) ${\overset{-}{v}}_{max}$ (cm^−1^): 3004, 2922, 2847, 2155, 2032, 1965, 1705, 1633, 1600, 1512, 1461, 1422, 1373, 1307, 1252, 1198, 1150, 1017, 981, 917, 863, 821, 769, 715, 670, 606, 537, 513. ^1^H NMR (300 MHz, CDCl_3_) *δ*: 3.83 (s, 3H), 5.23 (s, 2H), 6.35 (d, 1H, *J_trans_* = 15.9 Hz), 6.90 (d, 2H, *J* = 8.7 Hz), 7.22 (d, 2H, *J* = 8.6 Hz), 7.44 (d, 2H, *J* = 8.6 Hz), 7.48 (d, 2H, *J* = 8.7 Hz), 7.69 (d, 1H, *J_trans_* = 15.9). ^13^C NMR (75 MHz, CDCl_3_) *δ*: 55.4, 65.2, 114.4, 115.0, 120.4 (q, *J_C-F_* = 255.6 Hz), 121.0, 127.0, 129.7, 129.8, 135.0, 145.2, 149.0, 161.5, 167.0. MS, *m/z* (%): 352, C_18_H_15_F_3_O_4_, [M^+^], (85.5); 307 (69.9); 175 (100); 161 (60.3); 134 (78.8); 109 (34.4); 77 (27.3); 69 (25.5).

(*E*)-4-nitrobenzyl 3-(4-methoxyphenyl)acrylate (**4e**)

White solid, obtained in 73% yield, purified by silica gel column chromatography, eluting with hexane/ethyl acetate (3:2 *v*/*v*), m.p. 111.6***–***112.1 °C. TLC R_f_ = 0.50 hexane/ethyl acetate (3:2 *v*/*v*). IR (ATR) ${\overset{-}{v}}_{max}$ (cm^−1^): 3109, 3037, 2940, 2847, 2360, 2025, 1965, 1696, 1603, 1512, 1440, 1343, 1246, 1165, 1104, 1038, 1011, 984, 920, 857, 818, 733, 703, 558, 510. ^1^H NMR (300 MHz, CDCl_3_) *δ*: ^13^C NMR (75 MHz, CDCl_3_) *δ*: 3.84 (s, 3H), 5.33 (s, 2H), 6.37 (d, 1H, *J_trans_* = 15.9), 6.91 (d, 2H, *J* = 8.9 Hz), 7.49 (d, 2H, *J* = 9.2 Hz), 7.50 (d, 2H, *J* = 8.9 Hz), 7.71 (d, 1H, *J_trans_* = 15.9), 8.23 (d, 2H, *J* = 9.2 Hz). ^13^C NMR (75 MHz, CDCl_3_) *δ*: 55.4, 64.6, 114.3, 114.4, 123.8, 126.8, 128.3, 129.9, 143.6, 145.7, 147.7, 161.7, 166.8. MS, *m/z* (%): 313, C_17_H_15_NO_5_, [M^+^], (52.3); 296 (17.2); 266 (16.0); 161 (45.9); 134 (100); 89 (24.7); 77 (15.8).

(*E*)-4-chlorobenzyl 3-(4-methoxyphenyl)acrylate (**4f**)

White solid, obtained in 82% yield, purified by silica gel column chromatography, eluting with hexane/ethyl acetate (3:2 *v*/*v*), m.p. 79.4***–***79.7 °C. TLC R_f_ = 0.61 (hexane/ethyl acetate 3:2 *v*/*v*). IR (ATR) ${\overset{-}{v}}_{max}$ (cm^−1^): 3052, 2964, 2916, 2840, 2364, 2158, 2019, 1890, 1705, 1630, 1603, 1573, 1512, 1422, 1422, 1289, 1252, 1153, 1089, 975, 866, 818, 724, 549, 516. ^1^H NMR (300 MHz, CDCl_3_) *δ*: 3.83 (s, 3H), 5.20 (s, 2H), 6.34 (d, 1H, *J_trans_* = 15.9 Hz), 6.90 (d, 2H, *J* = 8.7 Hz), 7.34 (s, 4H), 7.49 (d, 2H, *J* = 8.7 Hz), 7.68 (d, 1H, *J_trans_* = 15.9 Hz). ^13^C NMR (75 MHz, CDCl_3_) *δ*: 55.4, 65.3, 114.3, 115.0, 127.0, 128.7, 129.6, 129.8, 134.1, 134.7, 145.1, 161.5, 167.0. MS, *m/z* (%): 304, C_17_H_15_ClO_3_, [M^+^ + 2], (22.1); 302 [M^+^] (64.0); 257 (67.0); 161 (70.0); 134 (85.1); 125 (100); 89 (52.6); 77 (26.7).

(*E*)-4-bromobenzyl 3-(4-methoxyphenyl)acrylate (**4g**)

White solid, obtained in 80% yield, purified by silica gel column chromatography, eluting with hexane/ethyl acetate (3:2 *v*/*v*), m.p. 75.9***–***77.0 °C. TLC R_f_ = 0.66 (hexane/ethyl acetate 3:2 *v*/*v*). IR (ATR) ${\overset{-}{v}}_{max}$ (cm^−1^): 2964, 2922, 2837, 2357, 1890, 1702, 1630, 1600, 1573, 1509, 1485, 1422, 1370, 1289, 1249, 1156, 1068, 975, 863, 824, 800, 772, 715, 549, 516. ^1^H NMR (300 MHz, CDCl_3_) *δ*: 3.83 (s, 3H), 5.18 (s, 2H), 6.34 (d, 1H, *J_trans_* = 15.9 Hz), 6.90 (d, 2H, *J* = 8.7 Hz), 7.26 (d, 2H, *J* = 8.7 Hz), 7.45***–***7.52 (m, 4H), 7.68 (d, 1H, *J_trans_* = 15.9 Hz). ^13^C NMR (75 MHz, CDCl_3_) *δ*: 55.4, 65.4, 114.4, 115.0, 122.2, 127.0, 129.8, 129, 131.7, 135.3, 145.1, 161.5, 167.0. MS, *m/z* (%): 348, C_17_H_15_BrO_3_, [M^+^ + 2], (16.2); 346 [M^+^] (16.2); 267 (27.31); 161 (100); 134 (60.1); 90 (29.2).

(*E*)-4-fluorobenzyl 3-(4-methoxyphenyl)acrylate (**4h**)

White solid, obtained in 74% yield, purified by silica gel column chromatography, eluting with hexane/ethyl acetate (3:2 *v*/*v*), m.p. 66.6***–***67.2 °C. TLC R_f_ = 0.56 (hexane/ethyl acetate 3:2 *v*/*v*). IR (ATR) ${\overset{-}{v}}_{max}$ (cm^−1^): 2964, 2922, 2837, 2360, 1019, 1884, 1708, 1633, 1603, 1576, 1509, 1422, 1289, 1222, 1147, 978, 863, 815, 754, 552. ^1^H NMR (300 MHz, CDCl_3_) *δ*: 3.83 (s, 3H), 5.20 (s, 2H), 6.34 (d, 1H, *J_trans_* = 15.9 Hz), 6.89 (d, 2H, *J* = 8.7 Hz), 7.09 (t, 2H, *J* = 8.7 Hz), 7.36***–***7.42 (m, 2H), 7.47 (d, 2H, *J* = 8.7 Hz), 7.68 (d, 1H, *J_trans_* = 15.9 Hz). ^13^C NMR (75 MHz, CDCl_3_) *δ*: 55.4, 65.4, 114.3, 115.1, 115.5 (d*, J* = 21.3 Hz), 115.6, 127.0, 129.8, 130.2 (d, *J* = 8.3 Hz), 132.1 (d, *J* = 3.1 Hz), 145.0, 161.5, 162.6 (d, *J* = 245.3 Hz), 167.1. MS, *m/z* (%): 286, C_17_H_15_O_3_F, [M^+^], (42.0); 241 (43.1); 161 (32.2); 134 (42.2); 109 (100).

(*E*)-3,4-difluorobenzyl 3-(4-methoxyphenyl)acrylate (**4i**)

White solid, obtained in 87% yield, purified by silica gel column chromatography, eluting with hexane/ethyl acetate (3:2 *v*/*v*), m.p. 84.4***–***84.6 °C. TLC R_f_ = 0.61 (hexane/ethyl acetate 3:2 *v*/*v*). IR (ATR) ${\overset{-}{v}}_{max}$ (cm^−1^): 3043, 2961, 2922, 2844, 2364, 2161, 2041, 1956, 1702, 1639, 1606, 1573, 1512, 1425, 1379, 1289, 1246, 1162, 1114, 1023, 978, 869, 812, 769, 609, 555, 516. ^1^H NMR (300 MHz, CDCl_3_) *δ*: 3.83 (s, 3H), 5.17 (s, 2H), 6.34 (d, 1H, *J_trans_* = 15.9 Hz), 6.90 (d, 2H, *J* = 8.7 Hz), 7.11***–***7.27 (m, 3H), 7.48 (d, 2H, *J* = 8.7 Hz), 7.68 (d, 1H, *J_trans_* = 15.9 Hz). ^13^C NMR (75 MHz, CDCl_3_) *δ*: 55.4, 64.8, 114.4, 114.8, 117.1***–***117.5 (m), 124.1***–***124.4 (m), 126.9, 129.8, 133.2***–***133.4 (m), 145.3, 148.3***–***148.7 (m), 151.7***–***152.0 (m), 161.6, 166.9. MS, *m/z* (%): 304, C_17_H_14_O_3_F_2_, [M^+^], (85.5); 286 (14.9); 259 (58.4); 177 (14.1); 161 (71.1); 134 (88.2), 127 (100).

(*E*)-2-bromobenzyl 3-(4-methoxyphenyl)acrylate (**4j**)

White solid, obtained in 57% yield, purified by silica gel column chromatography, eluting with hexane/ethyl acetate (3:2 *v*/*v*), m.p. 54.3***–***55.2 °C. TLC R_f_ = 0.61 (hexane/ethyl acetate 3:2 *v*/*v*). IR (ATR) ${\overset{-}{v}}_{max}$ (cm^−1^): 3043, 2925, 2837, 2357, 2158, 2041, 1974, 1720, 1633, 1594, 1509, 1440, 1310, 1259, 1207, 1147, 1017, 981, 821, 742, 664, 510. ^1^H NMR (300 MHz, CDCl_3_) *δ*: 3.83 (s, 3H), 5.33 (s, 2H), 6.38 (d, 1H, *J_trans_* = 15.9 Hz), 6.90 (d, 2H, *J* = 8.7 Hz), 7.19 (dt, 1H, *J* = 1.8 Hz, *J* = 7.8 Hz), 7.33 (dt, 1H, *J =* 1.5 Hz, *J* = 7.5 Hz), 7.44***–***7.50 (m, 2H), 7.59 (d, 2H, *J* = 8.7 Hz), 7.72 (d, 1H, *J_trans_* = 15.9 Hz). ^13^C NMR (75 MHz, CDCl_3_) *δ*: 55.4, 65.7, 114.3, 115.0, 123.4, 127.0, 127.5, 129.6, 129.8, 132.8, 135.6, 145.2, 161.5, 166.9. MS, *m/z* (%): 286, C_17_H_15_O_3_F, [M^+^], (42.0); 241 (43.1); 161 (32.2); 134 (42.2); 109 (100). MS, *m/z* (%): 348, C_17_H_15_BrO_3_, [M^+^ + 2], (47.3); 346 [M^+^] (47.6); 303 (36.1); 301 (37.6); 267 (11.0); 222 (49.1); 169 (50.8); 161 (81.0); 134 (100); 118 (18.9); 90 (70.0).

(*S,E*)-(4-(prop-1-en-2-yl)cyclohex-1-en-1-yl)methyl-3-(4-methoxyphenyl)acrylate (**4k**)

Yellow oil, obtained in 77% yield, purified by silica gel column chromatography, eluting with hexane/ethyl acetate (3:2 *v*/*v*). TLC R_f_ = 0.69 (hexane/ethyl acetate 3:2 *v*/*v*). IR (ATR) ${\overset{-}{v}}_{max}$ (cm^−1^): 3076, 2964, 2919, 2837, 2360, 1971, 1702, 1633, 1600, 1573, 1512, 1434, 1373, 1289, 1249, 1156, 1029, 981, 890, 827, 637, 549, 519. ^1^H NMR (300 MHz, CDCl_3_) *δ*: 1.73***–***1.75 (m, 3H), 1.82***–***2.23 (m, 7H), 3.83 (s, 3H), 4.59 (s, 2H), 4.71***–***4.74 (m, 2H), 5.78***–***5.83 (m, 1H), 6.33 (d, 1H, *J_trans_* = 15.9 Hz), 6.90 (d, 2H, *J* = 8.7 Hz), 7.47 (d, 2H, *J* = 8.7 Hz), 7.65 (d, 1H, *J_trans_* = 15.9 Hz). ^13^C NMR (75 MHz, CDCl_3_) *δ*

δ: 20.7, 26.4, 27.3, 30.5, 40.8, 55.3, 68.3, 108.8, 114.3, 115.6, 125.7, 127.2, 129.7, 132.8, 144.5, 149.6, 161.4, 167.2. MS, *m/z* (%): 312, C_20_H_24_O_3_, [M^+^], (10.7); 178 (33.5); 161 (100); 134 (45.8); 119 (26.7); 91 (34.5); 79 (18.4); 77 (18.1).

(*E*)-pyridin-4-ylmethyl 3-(4-methoxyphenyl)acrylate (**4l**)

Orange solid, obtained in 86% yield, purified by silica gel column chromatography, eluting with ethyl acetate/hexane (2:1 *v*/*v*), m.p. 90.1***–***92.4 °C. TLC R_f_ = 0.31 (ethyl acetate/hexane 2:1 *v*/*v*). IR (ATR) ${\overset{-}{v}}_{max}$ (cm^−1^): 3339, 2964, 2928, 2871, 2360, 2161, 2032, 1962, 1711, 1609, 1558, 1515, 1455, 1325, 1246, 1165, 1129, 1017. ^1^H NMR (300 MHz, CDCl_3_) *δ*: 3.82 (s, 3H), 5.24 (s, 2H), 6.37 (d, 1H, *J_trans_* = 15.9 Hz), 6.90 (d, 2H, *J* = 8.7 Hz), 7.28 (d, 2H, *J* = 6.3 Hz), 7.48 (d, 2H, *J* = 8.7 Hz), 7.71 (d, 1H, *J_trans_* = 15.9 Hz), 8.59 (d, 2H, *J* = 6.3 Hz). ^13^C NMR (75 MHz, CDCl_3_) *δ*: 55.4, 64.1, 114.4, 114.5, 121.9, 126.8, 129.9, 145.3, 145.6, 150.0, 161.6, 166.7. MS, *m/z* (%): 269, C_16_H_15_NO_3_, [M^+^], (100); 268 (68.7); 240 (19.1); 177 (43.3); 161 (92.2); 134 (96.5); 118 (23.1); 93 (61.6); 77 (26.0); 65 (33.8).

(*E*)-2,5-dimethoxybenzyl 3-(4-methoxyphenyl)acrylate (**4m**)

White solid, obtained in 59% yield, purified by silica gel column chromatography, eluting with ethyl acetate/hexane (2:1 *v*/*v*), m.p. 58.8***–***58.9 °C. TLC R_f_ = 0.68 ethyl acetate/hexane (2:1 *v*/*v*). IR (ATR) ${\overset{-}{v}}_{max}$ (cm^−1^): 3001, 2934, 2907, 2837, 2158, 2032, 1971, 1705, 1633, 1600, 1500, 1455, 1422, 1370, 1283, 1243, 1216, 1150, 1023, 981, 863, 827, 803, 715, 634, 552, 519. ^1^H NMR (300 MHz, CDCl_3_) *δ*: 3.78 (s, 3H), 3.81 (s, 3H), 3.83 (s, 3H), 5.27 (s, 2H), 6.38 (d, 1H, *J_trans_* = 15.9 Hz); 6.82***–***6.84 (m, 2H); 6.89 (d, 2H, *J* = 8.7 Hz); 6.97***–***6.99 (s, 1H), 7.47 (d, 2H, *J* = 8.7 Hz), 7.69 (d, 1H, *J_trans_* = 15.9 Hz). ^13^C NMR (75 MHz, CDCl_3_) *δ*: 55.4, 55.8, 56.1, 61.5, 111.6, 113.7, 114.3, 115.5, 115.6, 125.6, 127.2, 129.7, 144.6, 151.7, 153.5, 161.4, 167.2. MS, *m/z* (%): 328, C_19_H_20_O_5_, [M^+^], (43.2); 161 (100); 151 (44.3); 121 (38.8); 91 (19.0); 77 (16.2).

(*E*)-4-allyl-2-methoxyphenyl 3-(4-methoxyphenyl)acrylate (**4n**)

Yellow solid, obtained in 79% yield, purified by silica gel column chromatography, eluting with hexane/ethyl acetate (3:2 *v*/*v*), m.p. 105.8***–***107.6 °C. TLC R_f_ = 0.54 (hexane/ethyl acetate 3:2 *v*/*v*). IR (ATR) ${\overset{-}{v}}_{max}$ (cm^−1^): 3073, 3004 2967, 2928, 2837, 2161, 2010, 1766, 1726, 1633, 1600, 1509, 1458, 1422, 1307, 1262, 1201, 1171, 1117, 1032, 984, 914, 854, 821, 745, 655, 603, 513. ^1^H NMR (300 MHz, CDCl_3_) *δ*: 3.40 (d, 2H, *J* = 6.6 Hz), 3.83 (s, 3H), 3.85 (s, 3H), 5.08***–***5.16 (m, 2H), 5.91***–***6.05 (m, 1H), 6.54 (d, 1H, *J_trans_* = 15.9 Hz), 6.78***–***6.83 (m, 2H), 6.93 (d, 2H, *J* = 8.7 Hz), 7.02 (d, 1H, *J* = 8.1 Hz), 7.54 (d, 2H, *J* = 8.7 Hz), 7.83 (d, 1H, *J_trans_* = 15.9 Hz). ^13^C NMR (75 MHz, CDCl_3_) *δ*: 40.1, 55.4, 55.9, 112.8, 114.4, 114.5, 116.1, 120.7, 122.7, 127.1, 130.0, 137.1, 138.1, 138.9, 146.1, 151.1, 161.6, 165.5. MS, *m/z* (%): 161 (100); 133 (18.0); 77 (6.56).

(*E*)-2-methoxyphenyl 3-(4-methoxyphenyl)acrylate (**4o**)

White solid, obtained in 72% yield, purified by silica gel column chromatography eluting with hexane/ethyl acetate (3:2 *v*/*v*), m.p. 104.8***–***105.4 °C. TLC R_f_ = 0.55 (hexane/ethyl acetate 3:2 *v*/*v*). IR (ATR) ${\overset{-}{v}}_{max}$ (cm^−1^): 3007, 2940, 2837, 2038, 1726, 1627, 1597, 1500, 1455, 1419, 1310, 1246, 1201, 1171, 1111, 1020, 966, 863, 824, 754, 519. ^1^H NMR (300 MHz, CDCl_3_) *δ*: 3.84 (s, 3H), 3.85 (s, 3H), 6.55 (d, 1H, *J_trans_* = 15.9 HZ), 6.93 (d, 2H, *J* = 8.7 Hz), 6.97***–***7.10 (m, 2H), 7.12 (dd, *J* = 1.5, *J* = 8.0 Hz), 7.19***–***7.26 (m, 1H), 7.54 (d, 2H, *J* = 8.7 Hz), 7.84 (d, 1H, *J_trans_* = 15.9 Hz). ^13^C NMR (75 MHz. CDCl_3_) *δ*: 55.4, 55.9, 112.4, 114.4, 114.4, 120.8, 123.0, 126.8, 127, 130.0, 139.8, 146.3, 151.3, 161.6, 165.4.

#### 2.1.3. Preparation of (*E*)-*N*-Isopropyl-*N*-(Isopropylcarbamoyl)-3-(4-Methoxyphenyl)acrylamide (**4p**)

The attempted reaction between (*E*)-4-methoxy cinnamic acid (**1**) and bisabolol led to the formation of **4p** in 53% yield. The structure of this compound is supported by the following data.

White solid, purified by silica gel column chromatography, eluting with ethyl acetate (2:1 *v*/*v*), m.p. 120.6***–***121.8 °C. TLC R_f_ = 0.51 (ethyl acetate/hexane 2:1 *v*/*v*). IR (ATR) ${\overset{-}{v}}_{max}$ (cm^−1^): 3251, 3052, 2970, 2928, 2874, 2837, 2360, 2007, 1696, 1645, 1591, 1512, 1455, 1370, 1301, 1234, 1174, 1029, 978, 899, 824, 794, 691, 552, 507. ^1^H NMR (300 MHz, CDCl_3_) *δ*: 1.30 (d, 6H, *J* = 6.6 Hz), 1.52 (d, 6H, *J* = 6.9 Hz), 3.90 (s, 3H), 4.02***–***4.18 (m, 1H), 4.61 (sept, 1H, *J* = 6.9 Hz), 6.71 (d, 1H, *J_trans_* = 15.3 Hz), 6.96 (d, 2H, *J* = 8.7 Hz), 7.50 (d, 2H, *J* = 8.7 Hz), 7.66***–***7.72 (m, 2H). ^13^C NMR (75 MHz, CDCl_3_) *δ*: 21.1, 22.6, 42.8, 48.4, 55.4, 114.3, 117.0, 127.4, 129.6, 143.6, 154.2, 161.3, 168.2.

#### 2.1.4. Preparation of Compounds **5a**–**5o** Exemplified Using the (*E*)-Benzyl 3-(4-Methoxyphenyl)acrylate (**5a**)

To a 50 mL round bottom flask, benzyl alcohol (**3a**) (0.117 g, 1.08 mmol), dichloromethane (10.0 mL), (*E*)-3,4-dimethoxy cinnamic acid (**2**) (0.205 g, 0.986 mmol), DIC (0.124 g, 0.986 mmol), and DMAP (0.0120 g, 0.982 mmol) were added. The reaction mixture was magnetically stirred at room temperature for 45 min. After the reaction was completed, as TLC analysis confirmed, the reaction mixture was washed with distilled water (15.0 mL) and saturated sodium chloride solution (30.0 mL). The organic phase was reserved and the aqueous solution was extracted with dichloromethane (3 × 20.0 mL). The organic phases were combined, and the resulting organic layer was dried over sodium sulfate, filtered, and concentrated under residue pressure. The residue was submitted to purification using silica gel column chromatography, eluting with hexane/ethyl acetate (3:2 *v*/*v*). This procedure afforded compound **5a** with 62% yield (0.182 g, 0.611 mmol). The following data support the structure of **5a**.

Colorless oil, TLC R_f_ = 0.52 (hexane/ethyl acetate 3:2 *v*/*v*). IR (ATR) ${\overset{-}{v}}_{max}$ (cm^−1^): 3062, 3008, 2932, 2835, 2361, 2039, 1706, 1631, 1594, 1510, 1453, 1416, 1302, 1253, 1135, 1042, 972, 843, 803, 734, 695, 601,568, 523. ^1^H NMR (300 MHz, CDCl_3_) *δ*: 3.89 (s, 3H), 3.90 (s, 3H), 5.24 (s, 2H), 6.36 (d, 1H, *J_trans_* = 15.8 Hz), 6.85 (d, 1H, *J* = 8.4 Hz), 7.04 (d, 1H, *J* = 2.1 Hz), 7.10 (dd, *J* = 1.8 Hz, *J* = 8.4 Hz), 7.30***–***7.45 (m, 5H), 7.67 (d, 1H, *J_trans_* = 15.8 Hz). ^13^C NMR (75 MHz. CDCl_3_) *δ*: 55.8, 56.0, 66.3, 109.5, 111.0, 115.5, 122.7, 127.3, 128.2, 128.3, 128.6, 136.1, 145.1, 149.2, 151.1, 167.1. MS, *m/z* (%): 298, C_17_H_15_O_3_F, [M^+^], (86.6); 253 (17.1); 207 (20.0); 191 (34.3); 164 (62.2); 91 (100).

(*E*)-4-isopropylbenzyl 3-(3,4-dimethoxyphenyl)acrylate (**5b**)

Colorless oil, obtained in 66% yield, purified by silica gel column chromatography eluted with hexane/ethyl acetate (3:2 *v*/*v*). TLC R_f_ = 0.66 hexane/ethyl acetate (3:2 *v*/*v*). IR (ATR) ${\overset{-}{v}}_{max}$(cm^−1^): 2955, 2928, 2868, 2837, 2357, 2035, 1699, 1630 1594, 1512, 1461, 1422, 1337, 1301, 1252, 1135, 1020, 981, 842, 806, 760, 570. ^1^H NMR (300 MHz, CDCl_3_) *δ*: 1.25 (d, 6H, *J* = 6.9 Hz), 2.92 (sept, 1H, *J* = 6.9 Hz), 3.89 (s, 3H), 3.91 (s, 3H), 5.21 (s, 2H), 6.35 (d, 1H, *J_trans_* = 15.9 Hz), 7.04 (d, 1H, *J* = 1.8 Hz), 7.09 (dd, 1H, *J* = 1.8 Hz, *J* = 8.4 Hz), 7.25 (d, 2H, *J* = 7.8 Hz), 7.35 (d, 2H, *J* = 7.8 Hz), 7.66 (d, 1H, *J_trans_* = 15.9 Hz). ^13^C NMR (75 MHz, CDCl_3_) *δ*: 24.0, 33.9, 55.8, 56.0, 66.3, 109.5, 111.0, 115.6, 122.7, 126.7, 127.4, 128.5, 133.5, 145.0, 149.1, 149.2, 151.1, 167.1. MS, *m/z* (%): 310, C_21_H_24_O_4_, [M^+^], (100); 297 (35.4); 253 (19.2); 207 (37.1); 191 (53.3); 164 (64.5); 133 (87.8); 105 (59.7); 91 (39.7); 77 (16.6).

(*E*)-4-methoxybenzyl 3-(3,4-dimethoxyphenyl)acrylate (**5c**)

White solid, obtained in 62% yield, purified by silica gel column chromatography, eluting with hexane/ethyl acetate (1:1 *v*/*v*), m.p. 56.9***–***57.3 °C. TLC R_f_ = 0.56 (hexane/ethyl acetate 1:1 *v*/*v*). IR (ATR) ${\overset{-}{v}}_{max}$ (cm^−1^): 3004, 2931, 2831, 2038, 1980, 1699, 1627, 1594, 1512, 1461, 1419, 1301, 1237, 1132, 1020, 978, 806, 763, 603, 558, 522. ^1^H NMR (300 MHz, CDCl_3_) *δ*: 3.81 (s, 3H), 3.88 (s, 3H), 3.89 (s, 3H), 5.17 (s, 2H), 6.33 (d, 1H, *J_trans_* = 15.9 Hz), 6.84 (d,1H, *J* = 8.4 Hz), 6.90 (d, 2H, *J* = 8.7 Hz), 7.03 (d, 1H, *J* = 2.1 Hz), 7.08 (dd, 1H, *J* = 2.1 Hz, *J* = 8.4 Hz), 7.35 (d, 2H, *J* = 8.7 Hz), 7.64 (d, 1H, *J_trans_* = 15.9 Hz). ^13^C NMR (75 MHz, CDCl_3_) *δ*: 55.3, 55.8, 55.9, 66.1, 109.6, 111.0, 114.0, 115.7, 122.6, 127.4, 128.3, 130.1, 144.9, 149.2, 151.1, 159.6, 167.1.

(*E*)-4-(trifluoromethoxy)benzyl 3-(3,4-dimethoxyphenyl)acrylate (**5d**)

White solid, obtained in 78% yield, purified by silica gel column chromatography, eluting with hexane/ethyl acetate (3:2 *v*/*v*), m.p. 58.6***–***59.7 °C. TLC R_f_ = 0.51 (hexane/ethyl acetate 3:2 *v*/*v*). IR (ATR) ${\overset{-}{v}}_{max}$ (cm^−1^): 3007, 2940, 2834, 2364, 2032, 1699, 1633, 1594, 1509, 1467, 1440, 1419, 1340, 1259, 1138, 1050, 1014, 978, 917, 842, 806, 766, 670, 609, 576. ^1^H NMR (300 MHz, CDCl_3_) *δ*: 3.90 (s, 3H), 3.91 (s, 3H), 5.23 (s, 2H), 6.35 (d, 1H, *J_trans_* = 15.9 Hz), 6.86 (d, 1H, *J* = 8.1 Hz), 7.04 (d, 1H, *J* = 1.8 Hz), 7.10 (dd, 1H, *J* = 1.8 Hz, *J* = 8.1 Hz), 7.22 (d, 2H, *J* = 8.7 Hz), 7.44 (d, 2H, *J* = 8.7 Hz), 7.67 (d, 1H, *J_trans_* = 15.9 Hz). ^13^C NMR (75 MHz, CDCl_3_) *δ*: 55.9, 56.0, 65.2, 109.6, 111.0, 115.2, 120.4 (q, *J_C-F_* = 255.8 Hz), 121.1, 122.8, 127.2, 128.2, 129.7, 134.9, 145.4, 149.2, 151.3, 166.9. MS, *m/z* (%): 382, C_19_H_17_O_3_F_5_, [M^+^], (100); 337 (17.8); 207 (29.8); 175 (71.8); 164 (58.8); 109 (20.3); 77 (19.2).

(*E*)-4-nitrobenzyl 3-(3,4-dimethoxyphenyl)acrylate (**5e**)

White solid, obtained in 57% yield, purified by silica gel column chromatography, eluting with ethyl acetate/hexane (2:1 *v*/*v*); m.p. 134.2***–***134.6 °C. TLC R_f_ = 0.59 (ethyl acetate/hexane 2:1 *v*/*v*). IR (ATR) ${\overset{-}{v}}_{max}$ (cm^−1^): 3115, 3004, 2922, 2834, 2035, 1693, 1630, 1594, 1515, 1437, 1343, 1246, 1171, 1138, 1056, 1014, 972, 842, 800, 733, 676, 576, 534. ^1^H NMR (300 MHz; CDCl_3_) *δ*: 3.906 (s; 3H); 3.908 (s; 3H); 5.33 (s, 2H); 6.37 (d, 1H, *J_trans_* = 15.9 Hz); 6.87 (d, 1H, *J* = 8.1 Hz); 7.05 (d, 1H, *J* = 2.1 Hz); 7.12 (dd, 1H, *J* = 2.1 Hz, *J* = 8.1 Hz); 7.56 (d, 2H, *J* = 9.0 Hz); 7.69 (d, 1H, *J_trans_* = 15.9 Hz), 8.23 (d, 2H, *J* = 9.0 Hz). ^13^C NMR (75 MHz, CDCl_3_) *δ*: 55.9, 56.0, 64.7, 109.6, 111.0, 114.6, 122.9, 123.8, 127.0, 128.3, 143.6, 145.9, 147.7, 149.3, 151.5, 166.7. MS; *m/z* (%): 343; C_18_H_17_NO_6_; [M^+^], (100); 208 (13.9); 191 (30.9); 164 (82.7); 89 (22.4); 78 (20.8)

(*E*)-4-chlorobenzyl 3-(3,4-dimethoxyphenyl)acrylate (**5f**)

White solid, obtained in 86% yield, purified by silica gel column chromatography, eluting with hexane/ethyl acetate (3:2 *v*/*v*), m.p. 93.6***–***94.8 °C. TLC R_f_ = 0.55 (hexane/ethyl acetate 3:2 *v*/*v*). IR (ATR) ${\overset{-}{v}}_{max}$ (cm^−1^): 3025, 2998, 2934, 2837, 2357, 2158, 1986, 1714, 1636, 1597, 1509, 1488, 1455, 1346, 1301, 1259, 1153, 1077, 1017, 996, 839, 812, 769, 652, 603, 531. ^1^H NMR (300 MHz, CDCl_3_) *δ*: 3.89 (s, 3H), 3.90 (s, 3H), 5.19 (s, 2H), 6.34 (d, 1H, *J_trans_* = 15.8 Hz), 6.85 (d, 1H, *J* = 8.1 Hz), 7.03 (d, 1H, *J* = 2.1 Hz), 7.10 (dd, 1H, *J* = 2.1 Hz, *J* = 8.1 Hz), 7.34 (s, 4H), 7.66 (d, 1H, *J_trans_* = 15.8 Hz). ^13^C NMR (75 MHz, CDCl_3_) *δ*: 55.9, 56.0, 65.4, 109.6, 111.0, 115.2, 122.8, 127.2, 128.7, 129.6, 134.1, 134.7, 145.3, 149.2, 151.3, 166.9. MS, *m/z* (%): 334, C_18_H_17_ClO_4_, [M^+^ + 2], (35.8); 332 [M^+^] (98.9); 287 (25.5); 207 (36.5); 191 (45.4); 164 (87.2); 125 (100); 89 (43.2); 77 (25.0).

(*E*)-4-bromobenzyl 3-(3,4-dimethoxyphenyl)acrylate (**5g**)

White solid, obtained in 82% yield, purified by silica gel column chromatography, eluting with hexane/ethyl acetate (3:2 *v*/*v*), m.p. 94.4***–***95.8 °C. TLC R_f_ = 0.49 (hexane/ethyl acetate 3:2 *v*/*v*). IR (ATR) ${\overset{-}{v}}_{max}$ (cm^−1^): 3037, 2982, 2931, 2834, 2013, 1905, 1696, 1591, 1518, 1461, 1413, 1370, 1337, 1265, 1138, 1041, 1002, 969, 927, 845, 803, 763, 694, 576. ^1^H NMR (300 MHz, CDCl_3_) *δ*: 3.89 (s, 3H), 3.90 (s, 3H), 5.18 (s, 2H), 6.34 (d, 1H, *J_trans_* = 15.9 Hz), 6.85 (d, 1H, *J* = 8.4 Hz), 7.06 (d, 1H, *J* = 2.1 Hz), 7.10 (dd, 1H, *J* = 2.1 Hz, *J* = 8.4 Hz), 7.28 (d, 2H, *J* = 8.4 Hz), 7.50 (d, 2H, *J* = 8.4 Hz), 7.66 (d, 1H, *J_trans_* = 15.9 Hz). ^13^C NMR (75 MHz, CDCl_3_) *δ*: 55.9, 56.0, 65.4, 109.6, 111.0, 115.2, 122.2, 122.8, 127.2, 129.9, 131.7, 135.2, 145.4, 149.2, 151.3, 166.9.

(*E*)-4-fluorobenzyl 3-(3,4-dimethoxyphenyl)acrylate (**5h**)

White solid, obtained in 77% yield, purified by silica gel column chromatography, eluting with ethyl acetate/hexane (2:1 *v*/*v*), m.p. 78.4***–***78.7 °C. TLC R_f_ = 0.66 (ethyl acetate/hexane 2:1 *v*/*v*). IR (ATR) ${\overset{-}{v}}_{max}$ (cm^−1^): 3079, 3004, 2958, 2913, 2840, 2364, 2161, 2041, 1696, 1633, 1578, 1509, 1467, 1340, 1259, 1216, 1135, 1017, 975, 848, 794, 763, 558. ^1^H NMR (300 MHz, CDCl_3_) *δ*: 3.60 (s, 3H), 3.61 (s, 3H), 4.91 (s, 2H), 6.04 (d, 1H, *J_trans_* = 15.9 Hz), 6.56 (d, 1H, *J* = 8.4 Hz), 6.73***–***6.81 (m, 4H), 7.07***–***7.12 (m, 2H), 7.37 (d, 1H, *J_trans_* = 15.9 Hz). ^13^C NMR (75 MHz, CDCl_3_) *δ*: 55.6, 55.7, 65.2, 109.3, 110.7, 115.1, 115.2 (d, *J* = 21.3 Hz), 122.4, 127.0, 130.0 (d, *J* = 8.3 Hz,), 131.7 (d, *J* = 3.3 Hz), 144.9, 148.9, 150.9, 162.3 (d. *J* = 245.4 Hz), 166.6. MS, *m/z* (%): 316, C_18_H_17_O_4_F, [M^+^], (68.5); 271 (15.9); 207 (25.0); 191 (21.5); 164 (43.9); 109 (100).

(*E*)-3,4-difluorobenzyl 3-(3,4-dimethoxyphenyl)acrylate (**5i**)

White solid, obtained in 73% yield, purified by silica gel column chromatography, eluting with ethyl acetate/hexane (2:1 *v*/*v*), m.p. 83.9***–***84.3 °C. TLC R_f_ = 0.59 (ethyl acetate/hexane 2:1 *v*/*v*). IR (ATR) ${\overset{-}{v}}_{max}$ (cm^−1^): 3031, 2919, 2840, 2364, 2032, 1696, 1630, 1584, 1509, 1467, 1265, 1138, 1011,981, 939, 845, 806, 766, 612, 558. ^1^H NMR (300 MHz, CDCl_3_) *δ*: 3.90 (s, 3H), 3.91 (s, 3H), 5.17 (s, 2H), 6.34 (d, 1H, *J_trans_* = 15.9 Hz), 6.86 (d, 1H, *J* = 8.1 Hz), 7.04 (d,1H, *J* = 2.1 Hz), 7.08***–***7.27 (m, 4H), 7.66 (d, 1H, *J_trans_* = 15.9 Hz). ^13^C NMR (75 MHz, CDCl_3_) *δ*: 55.9, 56.0, 64.9, 109.6, 111.0, 115.0, 117.1***–***117.5 (m), 122.8, 124.2***–***124.4 (m), 127.2, 133.1***–***133.3 (m), 145.6, 148.4***–***149.2 (m), 151.3–152.0 (m), 152.0, 149.2, 151.3. MS, *m/z* (%): 334, C_18_H_16_O_4_F_2_, [M^+^], (100); 207 (23.0); 191 (32.2); 164 (65.9); 127 (87.0).

(*E*)-2-bromobenzyl 3-(3,4-dimethoxyphenyl)acrylate (**5j**)

White solid, obtained in 89% yield, purified by silica gel column chromatography, eluting with hexane/ethyl acetate (3:2 *v*/*v*), m.p. 69.5***–***70.3 °C. TLC R_f_ = 0.49 (ethyl acetate/hexane 2:1 *v*/*v*). IR (ATR) ${\overset{-}{v}}_{max}$ (cm^−1^): 3055, 3004, 2943, 2837, 2364, 2038, 1708, 1591, 1509, 1461, 1419, 1370, 1277, 1225, 1138, 1053, 1017. ^1^H NMR (300 MHz, CDCl_3_) *δ*: 4.38 (s, 3H), 4.38 (s, 3H), 5.80 (s, 2H), 6.86 (d, 1H, *J_trans_* = 15.9 Hz), 7.34 (d, 1H, *J* = 8.4 Hz), 7.53***–***8.08 (m, 6H), 8.17 (d, 1H, *J_trans_* = 15.9 Hz). ^13^C NMR (75 MHz, CDCl_3_)*δ*: 56.3, 56.4, 66.3, 110.1, 111.5, 115.7, 123.3, 124.0, 127.7***,*** 128.1, 129.3***,*** 129.5, 130.0***,*** 130.4, 133.0, 133.4, 136.0, 140.2, 145.9, 149.7, 151.7, 167.3. MS, *m/z* (%): 378, C_18_H_17_BrO_4_, [M^+^ + 2], (52.3); 376 [M^+^] (52.5); 191 (100); 164 (97.4).

(*S,E*)-(4-(prop-1-en-2-yl)cyclohex-1-en-1-yl)methyl 3-(3,4-dimethoxyphenyl)acrylate (**5k**)

Yellow oil, obtained in 59% yield, purified by silica gel column chromatography, eluting with ethyl acetate/hexane (2:1 *v*/*v*). TLC R_f_ = 0.69 (ethyl acetate/hexane 2:1 *v*/*v*). IR (ATR) ${\overset{-}{v}}_{max}$ (cm^−1^): 3076, 3007, 2922, 2837, 2038, 1705, 1630, 1594, 1509, 1437, 1337, 1304, 1249, 1138, 1020, 975, 887,803, 757, 649, 600, 548. ^1^H NMR (300 MHz, CDCl_3_) *δ*: 1.73***–***2.20 (m, 7H), 3.90 (s, 6H), 4.59 (s, 2H), 4.72 (s, 2H), 5.79***–***5.84 (m, 1H), 6.33 (d, 1H, *J_trans_* = 15.9 Hz), 6.86 (d, 1H, *J* = 8.4 Hz), 7.05 (d, 1H, *J* = 1.8 Hz), 7.10 (dd, 1H, *J* = 1.8 Hz, *J* = 8.4 Hz), 7.63 (d, 1H, *J_trans_* = 15.9 Hz). ^13^C NMR (75 MHz, CDCl_3_) *δ*: 20.7, 26.5, 27.3, 30.5, 40.8, 55.9, 68.4, 108.8, 109.6, 111.0, 115.8, 122.6, 125.8, 127.4, 132.8, 144.7, 149.2, 150.0, 151.1, 167.1.

(*E*)-pyridin-4-ylmethyl 3-(3,4-dimethoxyphenyl)acrylate (**5l**)

White solid, obtained in 64% yield, purified by silica gel column chromatography, eluting with ethyl acetate/hexane (2:1 *v*/*v*), m.p. 115.3***–***117.2 °C. TLC R_f_ = 0.25 (ethyl acetate/hexane 2:1 *v*/*v*). IR (ATR) ${\overset{-}{v}}_{max}$ (cm^−1^): 3336, 3031, 2964, 2919, 2871, 2837, 2360, 2167, 2032, 1962, 1847, 1714, 1627, 1600, 1576, 1512, 1446, 1576. ^1^H NMR (300 MHz, CDCl_3_) *δ*: 3.89 (s, 6H), 5.24 (s, 2H), 6.37 (d, 1H, *J_trans_* = 15.9 Hz), 6.86 (d, 1H), 7.07 (d, 1H), 7.12 (s, 1H), 7.28 (d, 2H, *J* = 6.3 Hz), 7.69 (d, 1H, *J_trans_* = 15.9 Hz), 8.59 (d, 2H, *J* = 6.3 Hz). ^13^C NMR (75 MHz, CDCl_3_) *δ*: 55.9, 56.0, 64.1, 109.6, 111.0, 114.7, 121.9, 122.9, 127.1, 145.9, 149.2, 150.0, 151.4, 166.6. MS, *m/z* (%): 269, C_17_H_17_NO_4_, [M^+^], (100); 268 (68.3); 240 (18.8); 177 (42.6); 161 (92.2); 134 (96.7); 118 (22.8); 93 (61.8); 91 (9.4); 77 (25.6); 65 (33.2).

(*E*)-2,5-dimethoxybenzyl 3-(3,4-dimethoxyphenyl)acrylate (**5m**)

White solid, obtained in 61% yield, purified by silica gel column chromatography, eluting with ethyl acetate/hexane (2:1 *v*/*v*), m.p. 84.1***–***85.2 °C. TLC R_f_ = 0.67 (ethyl acetate/hexane 2:1 *v*/*v*). IR (ATR) ${\overset{-}{v}}_{max}$ (cm^−1^): 3064, 2992, 2940 2910, 2828, 2161, 2038, 1977, 1823, 1702, 1633, 1597, 1506, 1422, 1376, 1292, 1252, 1219, 1138, 1044, 972, 848, 794, 754, 682, 600, 561. ^1^H NMR (300 MHz, CDCl_3_) *δ*: 3.78 (s, 3H), 3.81 (s, 3H), 3.90 (s, 3H), 3.90 (s, 3H), 5.27 (s, 2H), 6.38 (d, 1H, *J_trans_* = 15.9 Hz), 6.82***–***6.87 (m, 3H, H-3), 6.96***–***6.98 (m, 1H), 7.06 (d, 1H, *J* = 2.1 Hz), 7.10 (d, 1H, *J* = 2.1 Hz, *J* = 8.1 Hz), 7.67 (d, 1H, *J_trans_* = 15.9 Hz). ^13^C NMR (75 MHz, CDCl_3_) *δ*: 55.8, 55.9, 55.9, 56.1, 61.5, 109.5, 111.0, 111.6, 113.6, 115.7, 122.7, 125.6, 127.4, 144.9, 149.2, 151.1, 151.7, 153.5, 167.1. MS, *m/z* (%): 358, C_20_H_22_O_6_, [M^+^], (52.2); 191 (100); 164 (17.3); 151 (65.4); 121 (44.6); 91 (26.7).

(*E*)-4-allyl-2-methoxyphenyl 3-(3,4-dimethoxyphenyl)acrylate (**5n**)

White solid, obtained in 75% yield, purified by silica gel column chromatography, eluted with hexane/ethyl acetate (3:2 *v*/*v*), m.p. 124.1***–***125.9 °C. TLC R_f_ = 0.42 (hexane/ethyl acetate 3:2 *v/v*). IR (ATR) ${\overset{-}{v}}_{max}$ (cm^−1^): 3079, 3001, 2958, 2925, 2837, 2032, 1965, 1720, 1630, 1597, 1512, 1458, 1419, 1331, 1289, 1259, 1231, 1117, 1023, 978, 899, 854, 812, 760, 727, 652, 597, 543. ^1^H NMR (300 MHz, CDCl_3_) *δ*: 3.39 (d, 2H, *J* = 6.6 Hz), 3.82 (s, 3H), 3.92 (s, 3H), 3.92 (s, 3H), 5.07***–***5.15 (m, 1H), 5.91***–***6.04 (m, 1H), 6.54 (d, 1H, *J_trans_* = 15.9 Hz), 6.78***–***6.82 (m, 2H), 6.88 (d, 1H, *J* = 8.1 Hz), 7.02 (d, 1H, *J* = 8.7 Hz), 7.13 (d, 1H, *J* = 2.1 Hz), 7.15 (dd, 1H, *J* = 2.1 Hz, *J* = 8.1 Hz), 7.81 (d, 1H, *J_trans_* = 15.9 Hz). ^13^C NMR (75 MHz, CDCl_3_) *δ*: 40.1, 55.9, 56.0, 109.7, 111.0, 112.7, 114.7, 116.1, 120.7, 122.7, 123.0, 127.3, 137.1, 138.0, 138.9, 146.4, 149.2, 151.0, 151.4, 165.4. MS, *m/z* (%): 354, C_21_H_22_O_5_, [M^+^], (2.4); 191 (100); 163 (8.3).

(*E*)-2-methoxyphenyl 3-(3,4-dimethoxyphenyl)acrylate (**5o**)

White solid, obtained in 70% yield, purified by silica gel column chromatography, eluting with hexane/ethyl acetate (3:2 *v*/*v*), m.p. 130.0***–***131.4 °C. TLC R_f_ = 0.39 (hexane/ethyl acetate 3:2 *v*/*v*). IR (ATR) ${\overset{-}{v}}_{max}$ (cm^−1^): 3079, 3001, 2958, 2913, 2834, 2032, 1723, 1633, 1597, 1515, 1464, 1334, 1307, 1252, 1231, 1123, 1014, 981, 845, 803, 751, 594, 540. ^1^H NMR (300 MHz, CDCl_3_) *δ*: 3.84 (s, 3H), 3.919, 3.922 (s, 6H), 6.55 (d, 1H, *J_trans_* = 15.9 Hz), 6.88 (d, 1H, *J* = 8.1 Hz), 6.94***–***7.01 (m, 2H), 7.10***–***7.26 (m, 4H), 7.82 (d, 1H, *J_trans_* = 15.9 Hz). ^13^C NMR (75 MHz, CDCl_3_) *δ*: 55.9, 56.0, 109.7, 111.0, 112.4, 114.6, 120.8, 123.1, 126.8, 127.2, 139.8, 146.5, 149.2, 151.3, 151.4, 165.3.

The compounds **4a** [43], **4k** [44], **4n** [45], and **5a** [46] has been previously reported in the literature. Diamides similar to **4p** and **5p** has been described by Ramazani et al. [47]. The structure of **5o** was also investigated by X-ray diffractometry. Information pertaining to this investigation and the experimental procedures involved [48,49] can be found in the Supplementary Material.

#### 2.1.5. Preparation of (*E*)-3-(3,4-Dimethoxyphenyl)-*N*-Isopropyl-*N*-(Isopropylcarbamoyl)acrylamide (**5p**)

The attempted reaction between bisabol and (*E*)-3,4-cinnamic acid (**2**) resulted in the formation of **5p** in 64% yield. The structure of this compound is supported by the following data.

White solid, purified by silica gel column chromatography, eluting with ethyl acetate (2:1 *v*/*v*), m.p. 119.5***–***120.4 °C. TLC R_f_ = 0.51 (ethyl acetate/hexane 2:1 *v*/*v*). IR (ATR) ${\overset{-}{v}}_{max}$ (cm^−1^): 3251, 3079, 2976, 2928, 2834, 2161, 2038, 1983, 1699, 1642, 1597, 1509, 1452, 1379, 1310, 1265, 1138, 1062, 1020. ^1^H NMR (300 MHz, CDCl_3_) *δ*: 0.84 (d, 6H, *J* = 6.6 Hz), 1.04 (d, 6H, *J* = 6.6 Hz), 3.48 (s, 3H), 3.50 (s, 3H), 3.56***–***3.72 (m, 1H), 4.20 (sept, 1H, *J* = 6.6 Hz), 6.21 (d, 1H, *J_trans_* = 15.3 Hz), 6.44 (d, 1H, *J* = 8.4 Hz), 6.57 (d, 1H, *J* = 1.8 Hz), 6.66 (dd, *J* = 1.8 Hz, *J* = 8.4 Hz, 1H), 7.00 (d, 1H, *J* = 7.5 Hz), 7.19 (d, 1H, *J_trans_* = 15.3 Hz). ^13^C NMR (75 MHz, CDCl_3_) *δ*: 20.6, 22.2, 42.5, 47.7, 55.4, 55.5, 109.3, 110.7, 116.9, 121.9, 127.2, 143.3, 148.8, 150.6, 153.8, 167.2.

### 2.2. Biological Assays

#### 2.2.1. Cell Lines, Culture Cell Conditions and Sample Preparation

Herein, human tumor cells (lung adenocarcinoma—A549, and melanoma cell—SK-MEL-147) and normal cells (primary dermal fibroblast cell—CCD-1059Sk) were used. These cell lines were purchased from the Rio de Janeiro Cell Bank. Cells were maintained in DMEM/F12 (Dulbecco’s Modified Eagle’s Medium plus F12, Sigma Aldrich, Saint Louis, MO, USA) supplemented with 10% fetal bovine serum (FBS, Vitrocell, Campinas, Brazil). Cells were grown in a humidified atmosphere of 95% air and 5% CO_2_ under 37 °C.

The thirty-two synthesized compounds (monomethoxylates, **4a–4p** and dimethoxylates, **5a–5p**) and cinnamic acids (**1** and **2**) were dissolved in dimethyl sulfoxide (DMSO, Sigma Aldrich, Saint Louis, MO, USA), and the stock solution (20 mM) was stored at −20 °C until use. The compounds were solubilized in a fresh culture medium at final concentrations before experiments.

#### 2.2.2. Screening Strategy and Cell Viability Assay

Synthetic compounds (**4a−4p** and **5a−5p**) and cinnamic acids (**1** and **2**) were subjected to a screening assay that tested cell viability to select the most active compound(s) for subsequent assays.

Initially, cell viability was investigated using the sulforhodamine B (SRB) method [50]. For this purpose, A549 and SK-MEL-147 cells were seeded at a density of 1 × 10^4^ per well in 96-well plates. After attachment (24 h), cells were treated for 48 h with various compounds at a concentration of 40 μM (concentration used for preliminary analysis of cell viability). Cell monolayers were fixed with 10% (*w*/*v*) trichloroacetic acid (Sigma Aldrich, Saint Louis, MO, USA) at 4 °C for 1 h and stained with SRB (0.4% in 1% acetic acid) for 30 min. Samples were washed repeatedly with 1% acetic acid to remove unbound SRB. The protein-bound dye was dissolved in 10 mM Tris base solution, and the optical density was determined at 540 nm with a reference of 690 nm using a microplate reader. Subsequently, the concentration capable of inhibiting 50% of cell viability (IC_50_) was determined for the most active compound against A549, SK-MEL-147 and CCD-1059Sk. The concentrations used for dose–response curves were 0, 0.1, 1, 10, 100, and 1000 μM, and cell viability rates were determined using the SRB method, as previously described. DMSO (0.4% *v*/*v*) was used as a negative control and cisplatin as a positive control.

#### 2.2.3. The Colony Formation Assay

A549 lung adenocarcinoma cells were used for the clonogenic assay in 35 mm plates (200 cells/plate). After cell attachment (24 h), cells were treated with compound **4m** (20 and 40 μM) and DMSO (0.4%) for 24 h and then recovered in a drug-free medium for 12 days. Colonies were then fixed with methanol for 30 min and stained with crystal violet (1%) for 20 min. Only colonies with >50 cells were counted via direct visual inspection with a stereomicroscope at 20× magnification [51].

#### 2.2.4. Cell Cycle Analysis

Cell cycle analysis was performed according to Pressete [51]. Briefly, A549 cells (1 × 10^5^ cells/35 mm Petri plates) were treated with 20 or 40 μM **4m** for 24 h. Then, the samples were fixed overnight with 75% ethanol at 4 °C and rinsed twice with ice-cold phosphate-buffered saline (PBS). Cells were then homogenized in a dye solution (PBS containing 90 μg mL^−1^ propidium iodide and 1.5 mg mL^−1^ RNAase). Analysis was performed using a flow cytometer (Guava easyCyte 8HT, Luminex, Austin, TX, USA).

#### 2.2.5. Apoptosis Detection using Annexin V/7-AAD

According to the manufacturer’s instructions, a Guava Nexin^®^ kit (Merck Millipore, Darmstadt, Germany) was used to determine phosphatidylserine externalization. A549 cells were seeded in 24-well plates at a density of 1 × 10^5^ cells/well. Cell cultures were treated with 20 and 40 μM **4m** or 25 μM cisplatin for 24 or 48 h. Cells were then harvested via enzymatic digestion (Trypsin/EDTA, Sigma Aldrich, Saint Louis, MO, USA), and samples were centrifuged under 200× *g* for 5 min at 4 °C, washed with ice-cold PBS, and then 2 × 10^4^ cells were suspended in 100 μL DMEM. Next, 100 μL of a mixed solution of buffered Annexin V-PE (apoptosis detection kit) and 7-aminoactinomycin D (7-AAD, viability staining solution) was added. Samples were read after 20 min of incubation at room temperature in a dark chamber. Analysis was performed via flow cytometry using GuavaSoft 2.7 software.

#### 2.2.6. Immunoblot Analysis

A549 cells were seeded in 100 mm Petri dishes at 1 × 10^6^ cells/dish. After adhesion, the cells were treated with 40 µM **4m** for 24 and 48 h. Afterward, cells were homogenized in a radio-immunoprecipitation assay and urea lysis buffer (150 mM NaCl, 1.0% Nonidet p-40, 0.5% deoxycholate, 0.1% sodium dodecyl-sulfate (SDS), and 50 mM Tris at pH = 8.0) containing both protease and phosphatase inhibitors (#P8340, Sigma Aldrich, Saint Louis, MO, USA). Lysates were centrifuged (10,000× g) for 10 min at 4 °C. Supernatants were recovered, and total proteins were quantified (bicinchoninic acid (BCA) kit, Pierce Biotechnology Inc., Rockford, IL, USA) and resuspended in a Laemmli sample buffer of 62.5 mM Tris–HCl at pH = 6.8, 2% SDS, 10% glycerol, 5% 2-mercaptoethanol, and 0.001% bromophenol blue. An aliquot of 50 μg of protein was separated using sodium dodecyl-sulfate polyacrylamide gel electrophoresis (12%) and transferred (100 V, 250 mA for 2 h) onto a polyvinylidene fluoride membrane (Amersham Bioscience, Slough, Buckinghamshire, United Kingdom) and blocked with a blocking solution (5% nonfat milk in Tris-buffered saline (TBS) + 0.1% (*v*/*v*) Tween 20) at 4 °C for 1 h to prevent nonspecific protein binding. The membrane was probed with primary antibodies overnight at 4 °C: (Tyr 204) phosphorylated ERK (p-ERK) antibody (Santa Cruz Biotechnology, Dallas, Texas, USA; 1:200), ERK 1/2 (Santa Cruz Biotechnology; 1:200), Cyclin D1 and B (Santa-Cruz – 1: 200), phospho-histone H3 (Santa Cruz Biotehcnology; 1:200), and α-tubulin (Sigma Aldrich, Saint Louis, MO, USA; 1:1000). After washing with TBS-tween (0.1%), the membrane was incubated with the appropriate secondary peroxidase-conjugated antibody for 2 h at room temperature. Immunoreactive bands were visualized with an enhanced chemiluminescence western blotting detection kit (Amersham Pharmacia, Amersham, UK). A reprobing protocol was followed for detecting immunoreactive bands for different antibodies. Results were obtained from three independent experiments. Immunoreactive bands were quantified using the public domain ImageJ software (NIH, Bethesda, ML, USA) [52].

#### 2.2.7. Metastatic Behavior Assays

##### Cell Migration Assay

The wound healing assay was performed to evaluate the ability of compound **4m** to inhibit cell migration using a modified previously published methodology [42]. A549 cells were seeded at a concentration of 1.6 × 10^5^ cells/well on 24-well plates and allowed to reach confluence (90%) after overnight incubation at 37 °C and 5% CO_2_ atmosphere. The monolayers were then injured with a sterile 2000 μL pipette tip. Cells were washed twice with PBS to remove detached cells and then treated with 5, 10 and 20 µM of compound **4m**. DMSO (0.4% *v*/*v*) was used as a control. Photomicrographs were taken using a camera connected to an inverted microscope (40× magnification, Zeiss, Oberkochen, Germany). Wound closure rates were then quantitatively calculated as the difference between wound width at 0 h and 36 h. The area of the wound was quantified using the public program ImageJ (NIH, Bethesda, ML, USA). Results are expressed as a percentage of cell migration.

##### Cell Matrix Adhesion Assay

First, trypsinized A549 cells were treated with 5, 10, and 20 μM of compound **4m** or vehicle DMSO (0.4% *v*/*v*) for 30 min. Cells were then plated in 96-well plates (3 × 10^4^ cells/well), coated with matrigel (60 μL/well), and incubated at 37 °C for 4 h for adhesion. Then, the cells were washed with PBS, and the adherent cells were stained with toluidine blue (1% *v*/*v*, Sigma Aldrich, Saint Louis, MO, USA) and solubilized with SDS (1% *w*/*v*) at 37 °C for 30 min. The absorbance was measured at 540 nm. Photomicrographs were taken using a camera coupled to an inverted microscope (40× magnification; Oberkochen, Germany) [42].

#### 2.2.8. Transwell Invasion Assays

This assay was performed using transwell inserts with a pore diameter of 8 μm (Millicell, Ireland) based on protocols previously described [42]. Briefly, 60 μL of matrigel (BD Biosciences, São Paulo, São Paulo State, Brazil) diluted in serum-free medium (1:5) was added to the upper chambers, and incubated at 37 °C for 1 h. Subsequently, A549 cells were resuspended with serum-free DMEM/F12, treated with 5, 10, and 20 μM **4m**, and inoculated into a transwell chamber coated with matrigel (8 × 10^4^ cells, 400 μL/well). DMSO vehicle treatment (0.4% *v*/*v*) was used as a control. The lower chamber was filled with 500 μL of culture medium containing 20% *v*/*v* FBS as the chemoattractant. After 24 h, the chambers were fixed in methanol for 30 min, washed, and stained with toluidine blue (1% *v*/*v*) for 15 min. Cells were counted using an inverted microscope (BEL Photonics, Piracicaba, São Paulo State, Brazil).

#### 2.2.9. Statistical Analysis

The results were tested for significance using a *t*-test or one-way analysis of variance (ANOVA) followed by Dunnett’s posttest using GraphPad Prism^®^ 6.0. *p* values < 0.05 were considered statistically significant. Values are expressed as mean ± standard deviation.

## 3. Results

### 3.1. Preparation of Methoxylated Cinnamic Esters

**Scheme 1:**
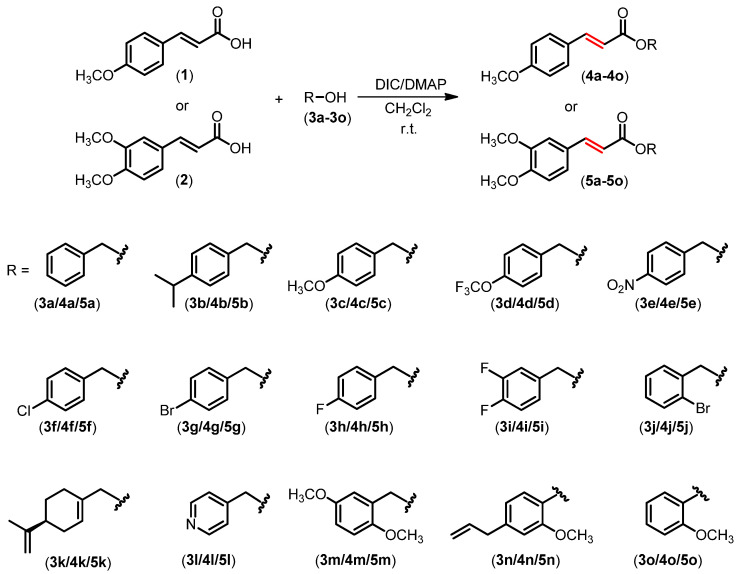
Preparation of cinnamic ester derivatives **4a–4o** and **5a–5o**.

A set of compounds derived from methoxylated cinnamic acids **1** and **2** was prepared, as depicted in Scheme 1, via Steglich esterification [53] of the carboxylic acids with different hydroxylated compounds, following the reported procedures described by Sova et al. [54].

Under the mild reaction conditions, thirty cinnamic acid derivatives were prepared with yields ranging from 57% to 86%. The compounds were characterized using nuclear ^1^H and ^13^C NMR and infrared (IR) spectroscopic techniques. The coupling constants of the hydrogens in the aliphatic double bonds (shown in red in Scheme 1) were 15.3–16.4 Hz, which agree with the *trans* configuration for the aliphatic double bonds. Carbonyl stretchings of the cinnamic acid derivatives shown in Scheme 1 were observed at ~1700 cm^−1^. Besides this, carbonyl functionality was confirmed from the signal observed at δ_C_ ≈ 170 ppm.

**Scheme 2:**
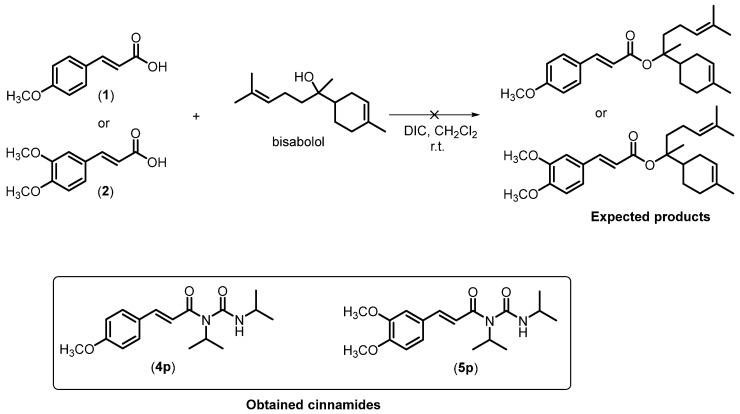
Formation of cinnamides **4p** and **5p** from cinnamic acids **1** and **2**.

We also attempted to conduct the Steglish reaction between the hydroxylated natural product bisabolol and the acids **1** and **2** (Scheme 2). However, the expected esters were not synthesized. Instead, the cinnamides **4p** and **5p** were obtained owing to the steric hindrance around the hydroxy group in bisabolol, which prevented ester formation.

Once synthesized, the compounds **4a–4p** and **5a–5p** were submitted to a screening assay, using cell viability as a parameter to select the most active compound against lung adenocarcinoma cell lines (A549) and melanoma cells (SKMEL-147).

### 3.2. Cytotoxicity of Compound ***4m*** against Lung Cancer Cells and Dermal Fibroblast

**Figure 2:**
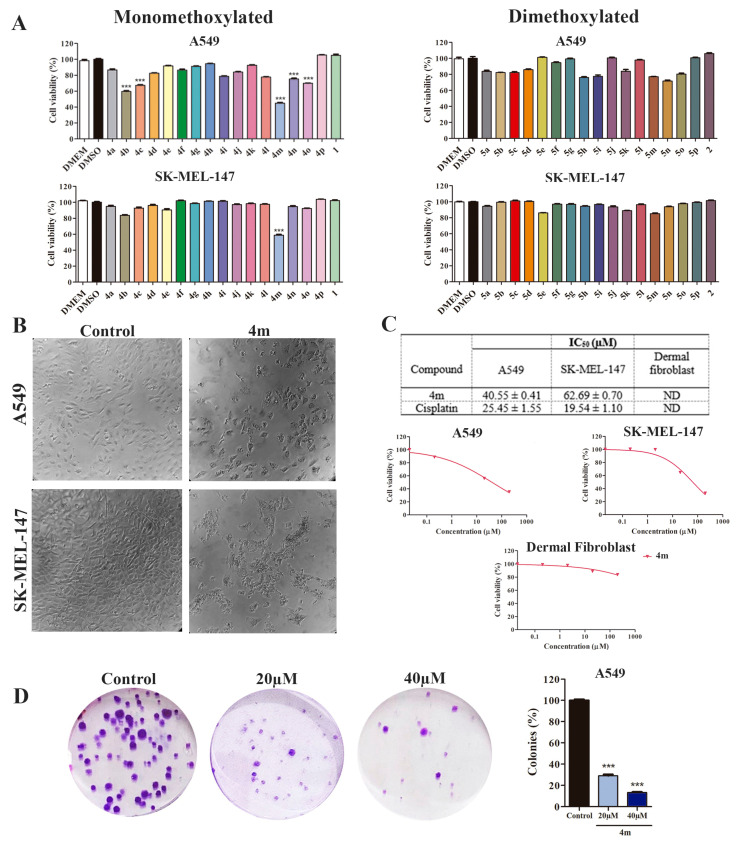
Monomethoxylated compound 4m is cytotoxic to lung adenocarcinoma A549 cells but not to primary dermal fibroblast. (**A**) Cell viability was determined by sulforhodamine B (SRB) after 48 h of treatment; **1** and **2** cinnamic acid. *** *p* < 0.001 compared to negative control DMSO according to ANOVA followed by Dunnet post-test (**B**) Illustrative images of the cell cultures at 48 h treatment with 40 μM **4m** (60× manignification). (**C**) IC_50_ (µM) values were determined from viability assay data. Cell cultures were treated with **4m** or cisplatin for 48 h. Growth curves determined by SRB assay after 48 h of treatment with compound **4m** against A549 cells, SK-MEL-147 cells, and dermal fibroblast cells. DMSO (0.1% *v*/*v*) was used as the negative control and cisplatin as the positive control. Non-tumor cell line: dermal fibroblast (CCD-1059Sk). ND: not determined once cell viability was not sufficiently reduced to determine IC_50_ values. (**D**) Clonogenic assay of A549 cells treated with 20 or 40 µM **4m** for 24 h and recovered in fresh medium for additional 12 days. *** *p* < 0.001 compared to control group according to ANOVA followed by Dunnet post-test.

The cytotoxic activity of compounds (**4a–4p**) and (**5a–5p**) derived from methoxylated cinnamic acids was investigated against two human tumor cell lines, lung cancer (A549) and melanoma (SK-MEL-147). The cell viability assay results show that at 40 μM, the monomethoxylated cinnamic acid esters **4b**, **4c**, **4m**, **4n**, and **4o** decreased the relative viability of A549 cells compared to the DMSO-treated cells (Figure 2A). In addition, compound **4m** also decreased the viability of the SK-MEL-147 cells compared with those treated with DMSO (Figure 2A). As for the dimethoxylated compounds (**5a–5p**), none of them showed remarkable cytotoxicity (Figure 2A). These results show that the monomethoxylated compound **4m** was the most active in decreasing the cell viability in both studied cell lines. However, its effect was more pronounced on the A549 cell line (cell viability = 44.83%) than on the SK-MEL-147 cell line (cell viability = 58.90%).

We constructed the dose–response curves to determine the IC_50_ values of **4m** on A549 and SK-MEL-147 cell lines (Figure 2B,C). The IC_50_ results confirm that **4m** was more effective against the A549 cell line (IC_50_ = 40.55 ± 0.41 µM) than the SK-MEL-147 cell line (IC_50_ = 62.69 ± 0.70 µM), thus showing better cytotoxic activity on lung cancer. However, the compound **4m** did not show an IC_50_ value lower than that of cisplatin on both cells tested. We also investigated the cytotoxicity of compound **4m** against normal cells (dermal fibroblast cells); no relevant results were observed (Figure 2C). These findings support the selection of compound **4m** for future studies against the A549 cell line.

A clonogenic assay was performed to examine the effect of **4m** on the proliferation dynamics of A549 cells. Our results show that **4m** has an antiproliferative effect on this cell line, as the abundance of colonies was lower in the groups treated with 20 and 40 µM compared to the cells treated with vehicle (0.4% *v*/*v* DMSO) (Figure 2D).

### 3.3. Cell Cycle Arrest and Apoptosis Analysis of Compound ***4m*** in A549 Cells

**Figure 3:**
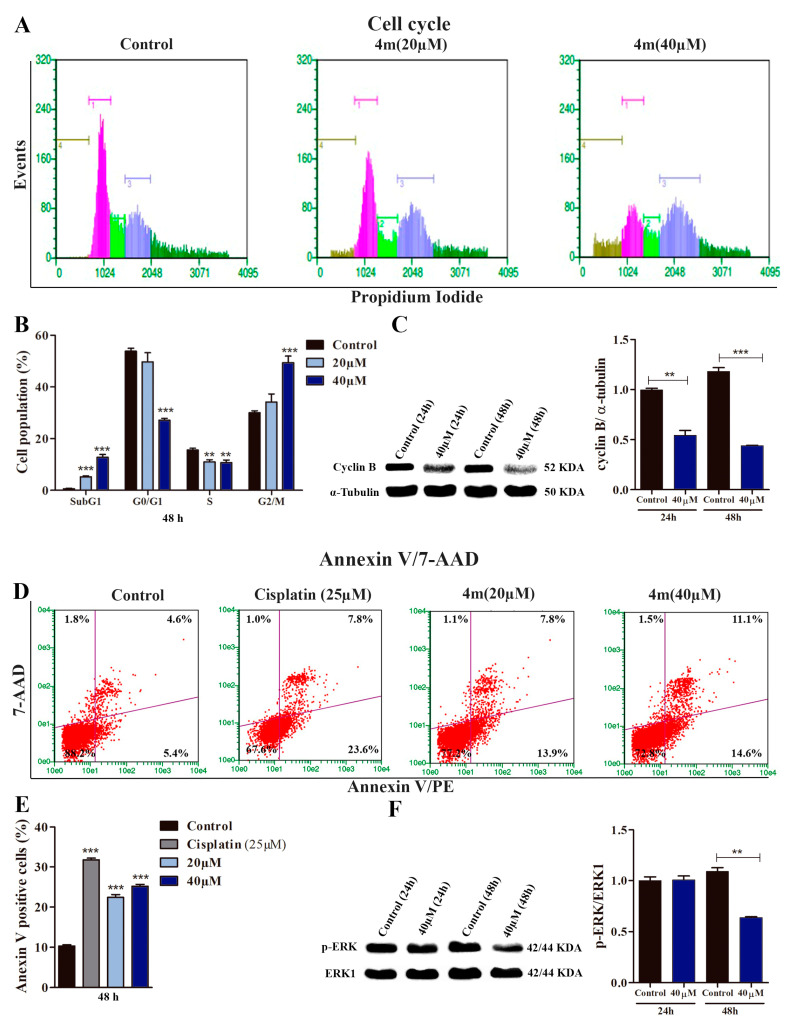
Compound 4m induces cell cycle arrest and apoptosis in A549 cells. (**A**) Illustrative histograms showing cell populations distributed in different phases of the cell cycle after 48 h of treatment with **4m**. Brown, pink, light green, blue, and dark green bars represent, respectively, sub-G1, G0/G1, S, G2/M, and hypertretraploid populations (DNA content higher than 4C). (**B**) Cell cycle analysis. ** *p* < 0.01 and *** *p* < 0.001 compared to control group according to ANOVA followed by Dunnet post-test. The hypertetraploid population was included in the analysis. (**C**) Relative protein levels of Cyclin B assessed by immunoblot in A549 cells treated for 24 and 48 h with 40 µM **4m**. α-tubulin was used as a loading control. ** *p* < 0.01 and *** *p* < 0.001 compared to control groups according to Student’s *t*-test. (**D**,**E**) Representative dot plots and analysis of annexin V/7-AAD assays performed in A549 cultures after 48 h treatment with **4m**. Viable cells (lower left quadrants), early apoptosis (lower right quadrants), late apoptosis (upper right quadrants), and necrotic cells (upper left quadrants). *** *p* < 0.001 compared to control group according to ANOVA followed by Dunnet post-test. (**F**) pERK expression determined by immunoblot in A549 cells treated for 24 or 48 h with 40 µM **4m**. ERK-1 was used as a loading control. ** *p* < 0.01 compared to control group according to Student’s *t*-test.

Cell cycle analysis showed that the progression dynamic was not altered in A549 cells treated with 20 µM **4m**, but an increase in the G2/M population was observed in A549 cells treated with 40 µM **4m**, indicating its ability to induce cell cycle arrest. Concurrent with the increase in the G2/M population treated with 40 µM **4m**, there was a considerable increase in the sub-G1 population, suggesting the cytotoxic activity of compound **4m** at this concentration (Figure 3A,B). Since treatment with 40 µM **4m** showed a considerable result in the cell cycle analysis, this concentration was selected to evaluate the expression profile of cyclin B at protein levels. Thus, it was observed that cell cycle arrest was accompanied by a decrease in cyclin B expression in A549 cells treated with 40 μM **4m** for 24 and 48 h (Figure 3C).

Considering the significant increase in G2/M population in A549 cells treated with 40 µM **4m** previously observed in cell cycle analysis (Figure 3A,B), we investigated the proapoptotic potential of **4m** in A549 cells using the Annexin V assay (Figure 3D,E). In this experimental approach, cisplatin was used as a positive control to validate the data obtained using flow cytometry. We found an increased frequency of Annexin V-positive cells in cells treated with 20 and 40 μM **4m** for 48 h (Figure 3D,E).

### 3.4. Effect of Compound ***4m*** on the Metastatic Behavior of A549 Cells

**Figure 4:**
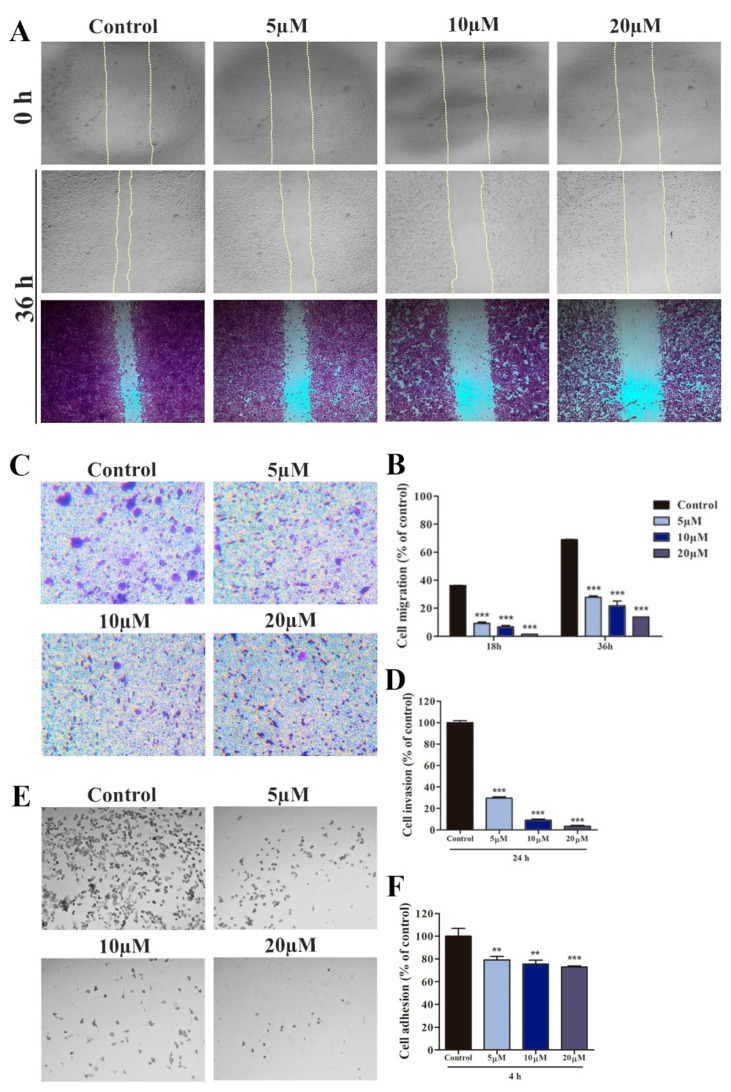
Compound 4m reduces the metastatic behavior of A549 cells. (**A**) Photomicrography represents the wound taken at 0 and 36 h (methanol for 30 min and crystal violet (1%) for 20 min) after treatment with 5, 10, and 20 μM **4m** (40× magnification). (**B**) The bar graphs represent migrative cell number that was treated with 5, 10 and 20 μM of compound **4m** after 18 and 36 h. (**C**) Photomicrography represents the cells invasion through matrigel coating (100× magnification). (**D**) The bar graphs represent invasive cell number that was treated with 5, 10, and 20 μM **4m** after 24 h. (**E**) Photomicrography represents the cells adhered on matrigel coating (40× magnification). (**F**) The bar graph represents the adhered cell number that was treated with with 5, 10, and 20 μM **4m** after 6 h. Statistical significance was determined by ANOVA and Dunnet post-test, ** *p* ˂ 0.01 and *** *p* ˂ 0.001 compared to control groups.

To investigate the effect of compound **4m** on A549 cell migration, we performed a wound-healing assay with 5, 10, and 20 µM **4m,** and evaluated cell motility. We found that compound **4m** at concentrations of 10 and 20 μM could inhibit cell migration compared to DMSO-treated cells (Figure 4A,B). In addition, we investigated the effect of compound **4m** on the invasion and adhesion of A549 cells. The invasion of A549 cells decreased after 24 h of treatment with 5 (70.4%), 10 (91%), and 20 (96.6%) μM of compound **4m** compared with DMSO-treated cells (Figure 4B,C). Similarly, cell adhesion was significantly decreased at 5 (20.8%), 10 (24.3%), and 20 μM (27%) **4m** compared to DMSO-treated cells (Figure 4E,F).

## 4. Discussion

The antitumor potential of cinnamic acid and its derivates have been previously reported [14,55,56]; however, few studies explore methoxylated cinnamic esters as antitumor prototypes. Thus, this study aimed to synthesize two series of compounds (monomethoxylated and dimethoxylated cinnamic esters) to evaluate their antitumor potential. Thirty cinnamate esters (compounds **4a–4o** obtained from 4-methoxy cinnamic acid and **5a–5o** derived from 3,4-dimethoxy acid, Figure 1) were obtained. Two cinnamides (compounds **4p** and **5p**) were also prepared when attempting to synthesize corresponding esters by reacting the methoxylated cinnamic acids and bisabolol. These substances were submitted to in vitro screening tests [57], and compound **4m** was eligible for further investigations based on its cytotoxic profiles on tumor and normal cells. Herein, the IC_50_ values obtained for **4m** in this study were much lower than other di- or trimethoxylated cinnamic acid derivatives previously reported [58,59].

The cancer cells used as a study model in this investigation harbor mutations in the RAS oncogene, responsible for the hyperactivation of RAS signaling [60,61] and downstream signaling pathways, including MAPK/ERK. The SK-MEL-147 cells have a mutation in NRAS (Q61R) [60], whereas the A549 cells cause a mutation in KRAS (G12S) [61]. The A549 cells also harbor an inactivation mutation in the tumor suppressor gene CDKN2A, which encoded p16^INK4^ and p14^ARF^ proteins by alternative splicing. These proteins negatively regulate cell cycle progression via retinoblastoma protein (pRB) and p53 gene activity, respectively. Considering that A549 cells were more responsive to **4m** treatment than SK-MEL-147 cells, they were used for further investigations.

The antiproliferative activity of **4m** on A549 cells was demonstrated using clonogenic assay and cell cycle analysis. There was a considerable reduction in colony frequencies in samples treated with **4m**, suggesting that the treatment drastically affected the clonogenic capacity of A549 cells. Apparently, the size of the colonies in **4m**-treated samples was smaller than the colonies in the control group, suggesting that the cells exposed to the tested compound had fewer cycles of division than the non-exposed samples. In fact, **4m** inhibited the cell cycle progression in A549 cells, as demonstrated by a considerable increase in the G2/M population in A549-treated cultures. The effects of cinnamic acid or its derivatives on the cell cycle of lung adenocarcinoma cells have not been studied yet; however, our results are in accordance with Anantharaju et al. (2017)*,* [57] who showed the inhibitory effect of dihydroxy cinnamic acid (caffeic acid) on the cell cycle progression of human colon cancer cells (HCT116 and HCT-15). They showed that cancer colon cells were arrested in S and G2/M phases in response to treatment with caffeic acid. The pharmacological agents that can interrupt the cell cycle progression are recognized as potential anticancer agents [55].

The G2/M transition is highly regulated by the orchestrated activity of kinase and phosphatase proteins. Importantly, the activation of cyclin-dependent kinase 1 (CDK1)–cyclin B complexes represents a critical event in the transition from G2 to the M phase [62,63]. Thus, the expression levels of cyclin B were assessed using Western blotting. Compound **4m** at 40 µM considerably reduced the cyclin B expression in A549 cells. This finding is very promising and indicates that the inhibitory effect of **4m** on the proliferation of A549 is associated with cyclin B downregulation. Recently, it was reported that cyclin B is highly expressed in patients with lung adenocarcinoma, which was associated with a poor prognosis [64]. In addition, Wang et al. (2019) [65] demonstrated that cyclin B is not involved in the apoptosis induction, migration, and invasion of NSCLC, but is directly associated with proliferation cycles. The reduction in cyclin B levels promoted cell cycle arrest at G2/M, diminishing the dimension of xenograft tumors.

Cell cycle analysis also revealed an accumulation of cells in the SubG1 phase in A549 cultures treated for 48 h with **4m,** suggesting that this compound can induce cell death. Cell cycle arrest may trigger signals culminating in apoptosis [66]. Thus, the proapoptotic activity of **4m** on A549 cells was investigated. The results show an increased frequency of cells positive for annexin V in treated cultures when compared to controls, suggesting that the cytotoxic activity of **4m** on A549 cells, at least in part, involves apoptosis induction. The eduction of cyclin B, cell cycle arrest at G2/M, and apoptosis induction were reported in A549 cells treated with natural diterpenoids [67,68].

Considering that MAPK signaling pathway is associated with cell proliferation and survival [69] and that A549 cell harbor activating mutation in KRAS, the influence of **4m** on the MAPK/ERK signaling pathway was investigated. The Western blotting analysis revealed that **4m** considerably reduced p-ERK expression, suggesting that RAS/RAF/MEK/ERK signaling was attenuated in A549 cells in response to treatment, reinforcing the antitumor activity of **4m**. Cell cycle arrest at G2/M, apoptosis induction, and reduced ERK activation levels were also observed in NSCLC cell lines (H2122, H358, H460) containing KRAS mutation in response to small molecule 0375–0604 treatment, a KRAS inhibitor according to docking studies [70]. Similar results were found by Li et al. (2021) [71], who treated NSCLC (H358) with stapled peptide-based RAS inhibitors. In addition, Tsai et al. (2013) [21] reported that cinnamic acid (cis-CA and trans-CA) could inhibit the MAPK/ERK signaling in A549 cells. Therefore, the findings obtained in this work suggest that the antiproliferative activity of **4m** on A549 cells is related to its ability to modulate the MAPK/ERK signaling pathway and regulate the cell cycle.

Since metastasis is the general cause of cancer deaths, including lung cancer [72], some in vitro parameters such as adhesion, migration, and invasion were evaluated to predict the ability of **4m** to inhibit lung cancer metastasis. The results show that **4m** could inhibit the adhesion, migration, and invasion capacity of A549 cells. Furthermore, in vivo studies should be performed to validate these in vitro findings; however, our data are in accordance with those of Tsai et al. (2013) [56], who described the antimetastatic potential of cinnamic acid derivatives using A549 cell line as study model. We have previously reported that phenyl 2,3-dibromo-3-phenylpropanoate, a cinnamic acid derivative compound, has antimetastatic potential against melanoma cells [42].

The synthesis of cinnamate esters was planned to assess the effects of different R groups (benzyl, phenyl and alicyclic groups) against the aforementioned tumor cell lines. Compound **4m** was derived from 4-methoxy cinnamic acid, in which R corresponds to the 4-methoxy benzyl. Thus, herein, combining the 4-methoxy cinnamoyl portion with the 4-methoxy-benzyloxy group resulted in the most effective compound, **4m**, on the tumor cell lines.

## 5. Conclusions

Herein, thirty-two cinnamic acid derivatives were synthesized and characterized using spectroscopic methods. Compound 4m, a monomethoxylated cinnamate ester derivative, was identified as the lead compound based on cytotoxicity studies against SK-MEL-147 (melanoma) and A549 (lung adenocarcinoma) cells. It effectively inhibited the proliferation, migration, and invasion of A549 cells. The antiproliferative activity of 4m on A549 cells was associated with its ability to reduce cyclin B expression and ERK activation, leading to cell cycle arrest at G2/M and, ultimately, apoptosis. Our findings indicate that combining a 4-methoxy cinnamoyl portion with the 4-methoxy-benzyloxy group may improve the antitumor activity of methoxylated cinnamic acid derivatives.

## Data Availability

Not applicable.

## Supplementary Materials

The following supporting information can be downloaded at: https://www.mdpi.com/article/10.3390/life13071428/s1, Figures S1–S32: Infrared (IR) spectra of compounds **4a–4p** and **5a–5p**; Figures S33–S64: ^1^H Nuclear Magnetic Resonance (NMR) spectra of compounds **4a–4p** and **5a–5p**; Figures S65–S86: ^13^C Nuclear Magnetic Resonance (NMR) spectra of compounds **4a–4p** and **5a–5p**; Figures S87–S111: Massa Spectra of selected compounds; Table S1: Crystallographic data and refinements for compound **5o**; Figure S112: Asymetric unit of compound **5o**.
