# Supplementary material for: Methoxylated Cinnamic Esters with Antiproliferative and Antimetastatic Effects on Human Lung Adenocarcinoma Cells
Source: Life (Basel). 2023 Jun 22;13(7):1428. doi: 10.3390/life13071428 (PMC10381754; doi:10.3390/life13071428)
Supplement: Supplementary file 1 [file life-13-01428-s001.zip › life-2323055-supplementary.pdf]

Supplementary material

Methoxylated Cinnamic Esters with Antiproliferative and Antimetastatic Effects on Human Lung Adenocarcinoma Cells

João Graciano Sampaio [1], Carolina Girotto Pressete [2], Adilson Vidal Costa [3], Felipe Terra Martins [4], Graziela Domingues de Almeida Lima [2], Marisa Ionta [2,*] and Róbson Ricardo Teixeira [1,*]

[1] Grupo de Síntese e Pesquisa de Compostos Bioativos (GSPCB), Departamento de Química, Universidade Federal de Viçosa, Viçosa-MG, 36570-900 Brazil; joaogracianojg@hotmail.com
[2] Programa de Pós-Graduação em Biociências Aplicadas à Saúde, Instituto de Ciências Biomédicas, Universidade Federal de Alfenas, Alfenas-MG, 37130–000 Brazil; carolina.pressete@sou.unifal-mg.edu.br (C.G.p.); graziela.lima@unifal-mg.edu.br (G.D.d.A.L.)
[3] Departamento de Química e Física, Universidade Federal do Espírito Santo, Alto Universitário, s/n, Guararema, Alegre-ES, 29500–000 Brazil; avcosta@hotmail.com
[4] Departamento de Química, Universidade Federal de Goiás, Goiânia-GO, 74690-900 Brazil; felipe@ufg.br
*Correspondence: marisa.ionta@unifal-mg.edu.br (M.I.); robsonr.teixeira@ufv.br (R.R.T.)

INFRARED (IR) SPECTRA OF COMPOUNDS

4a–4p AND 5a–5p

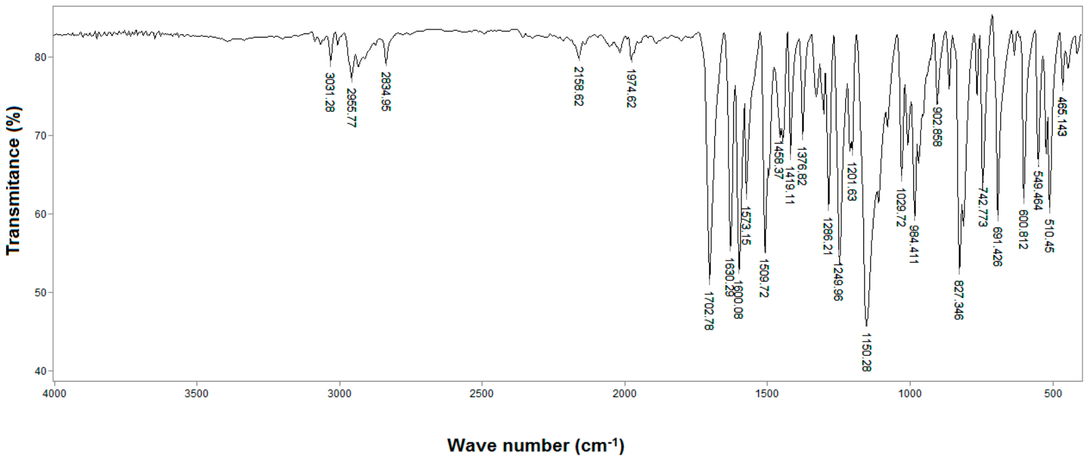

Figure S1 – Infrared spectrum (ATR) of **4a**.

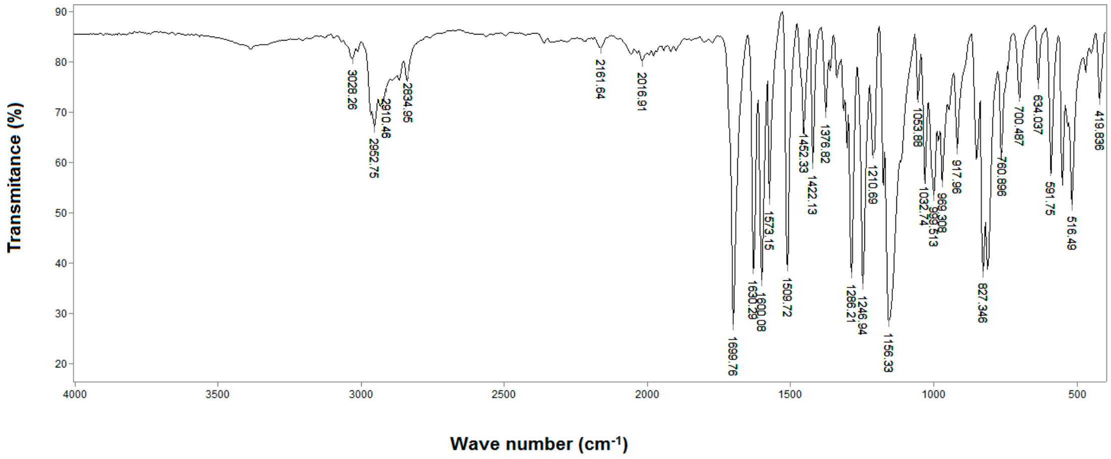

Figure S2 – Infrared spectrum (ATR) of **4b**.

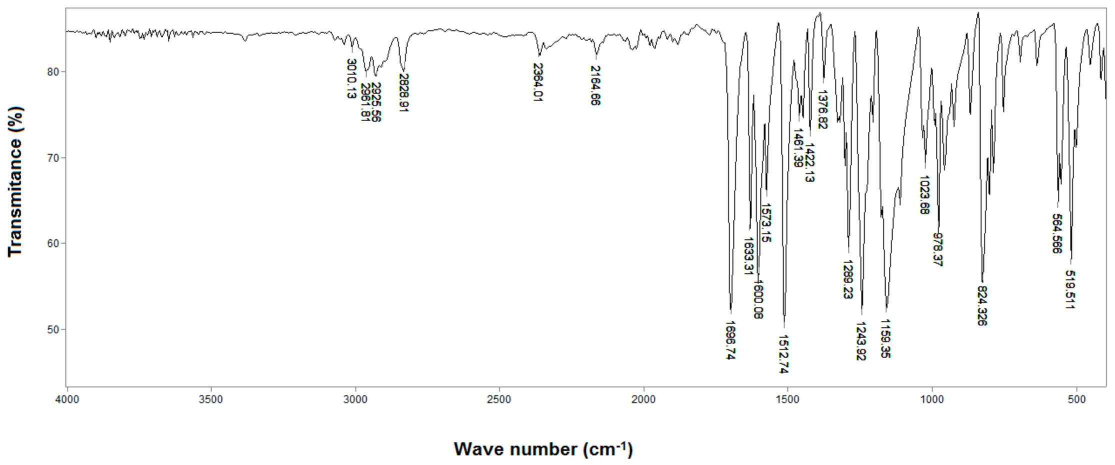

Figure S3 – Infrared spectrum (ATR) of **4c**.

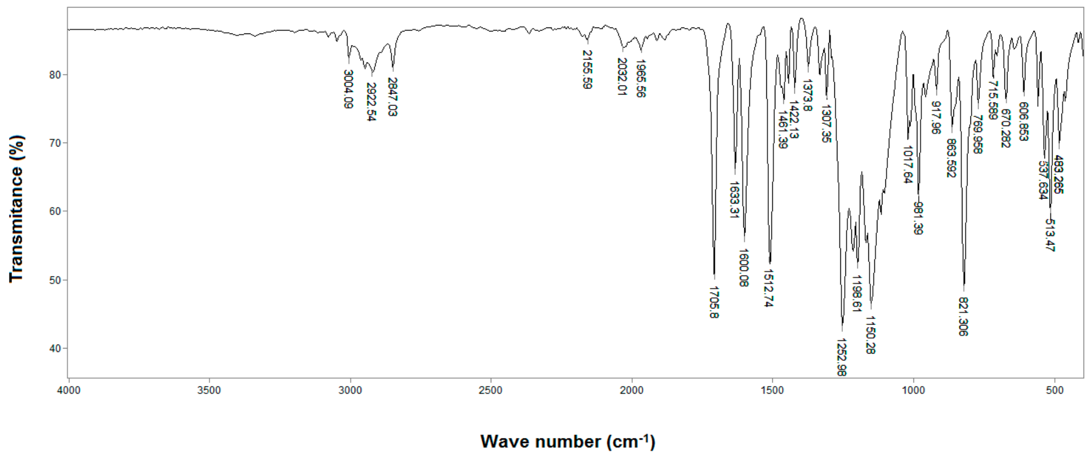

Figure S4 – Infrared spectrum (ATR) of **4d**.

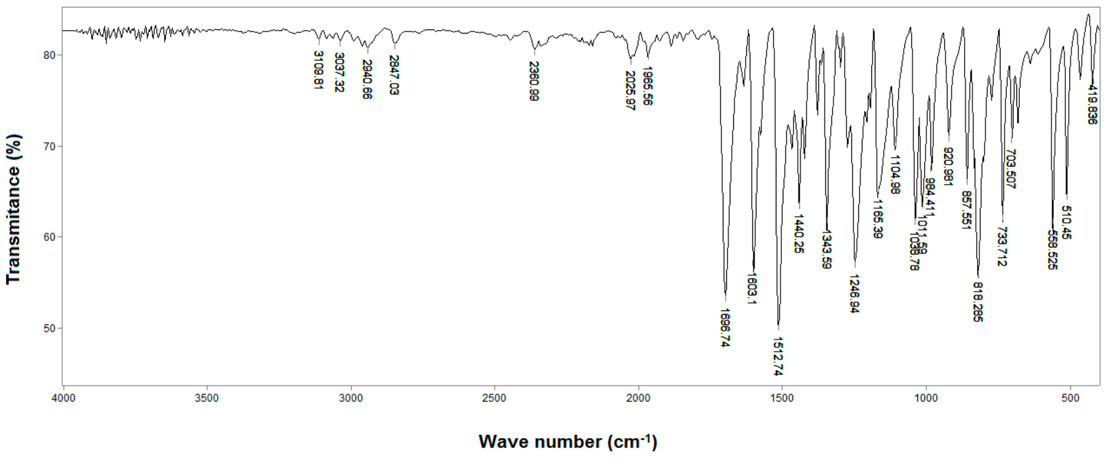

Figure S5 – Infrared spectrum (ATR) of **4e**.

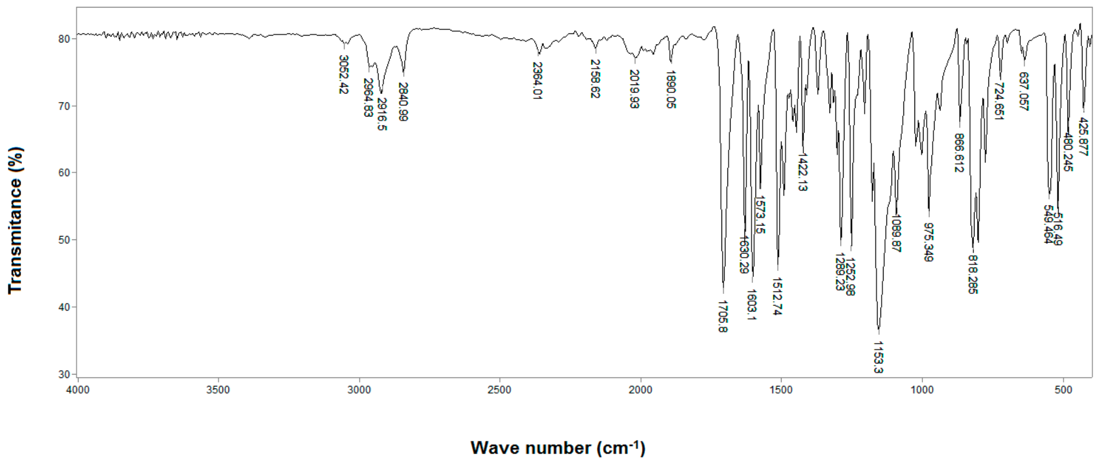

Figure S6 – Infrared spectrum (ATR) of **4f**.

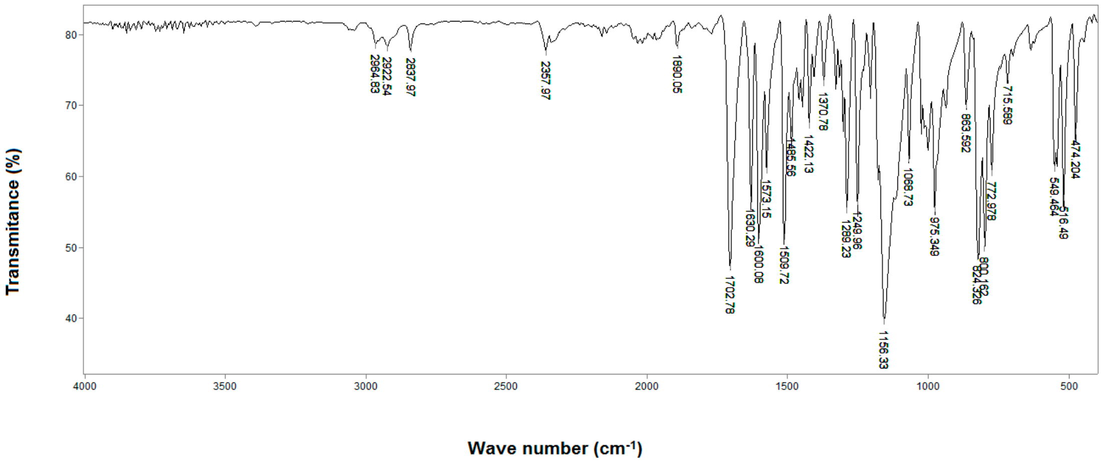

Figure S7 – Infrared spectrum (ATR) of **4g**.

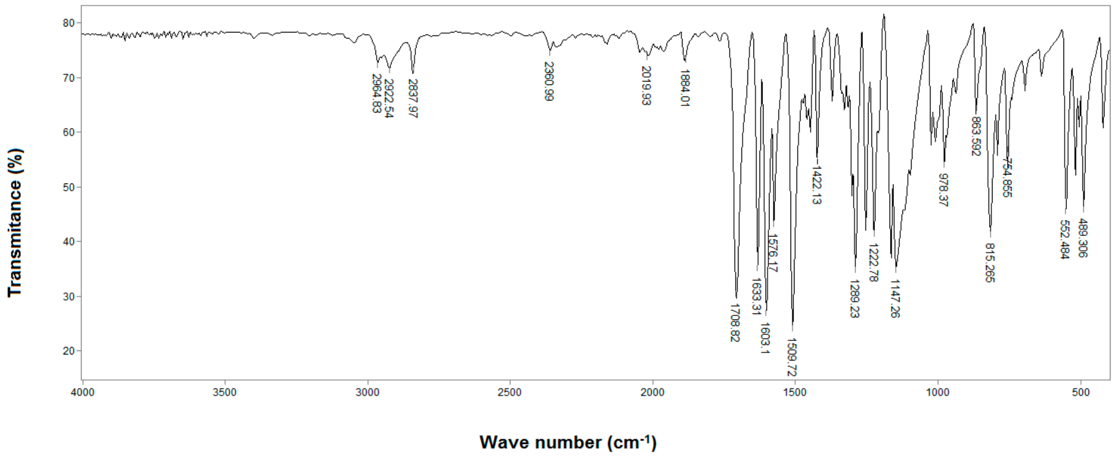

Figure S8 – Infrared spectrum (ATR) of **4h**.

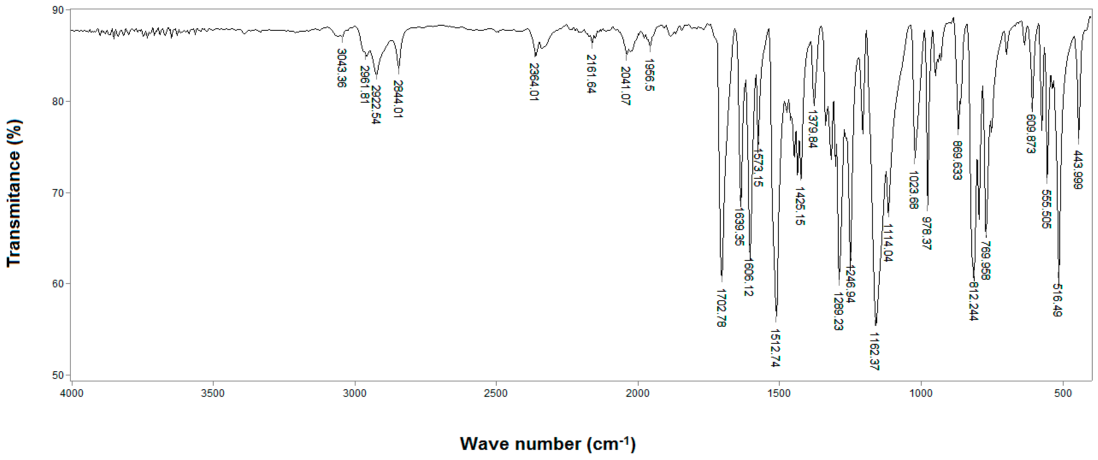

Figure S9 – Infrared spectrum (ATR) of **4i**.

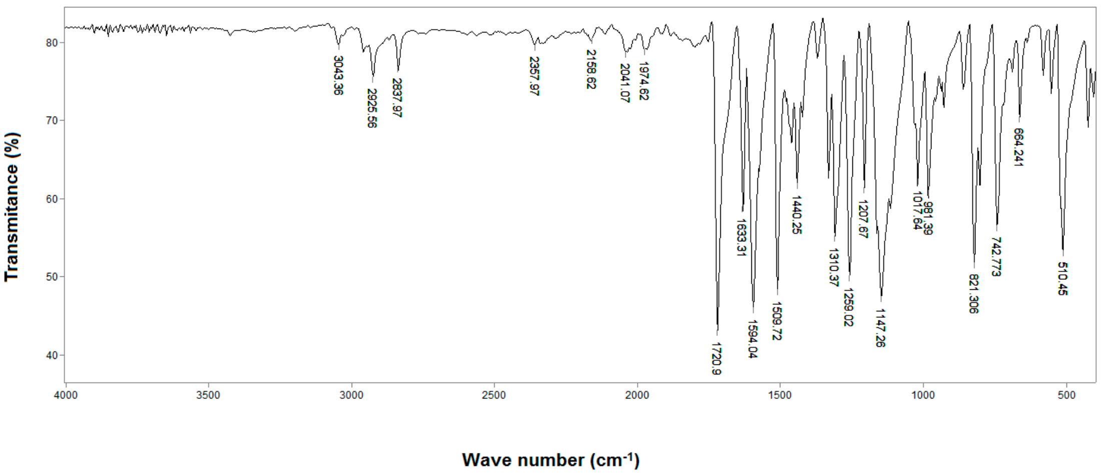

Figure S10 – Infrared spectrum (ATR) of **4j**.

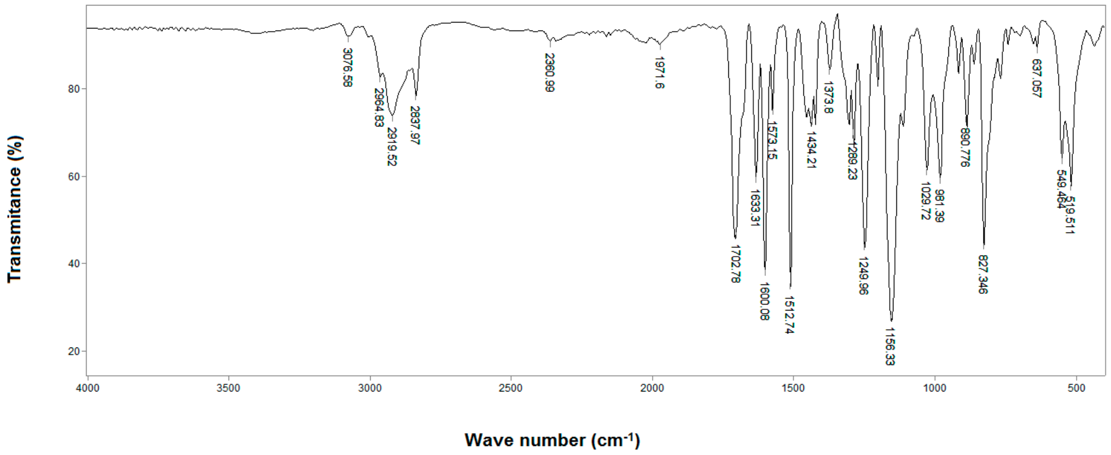

Figure S11 – Infrared spectrum (ATR) of **4k**.

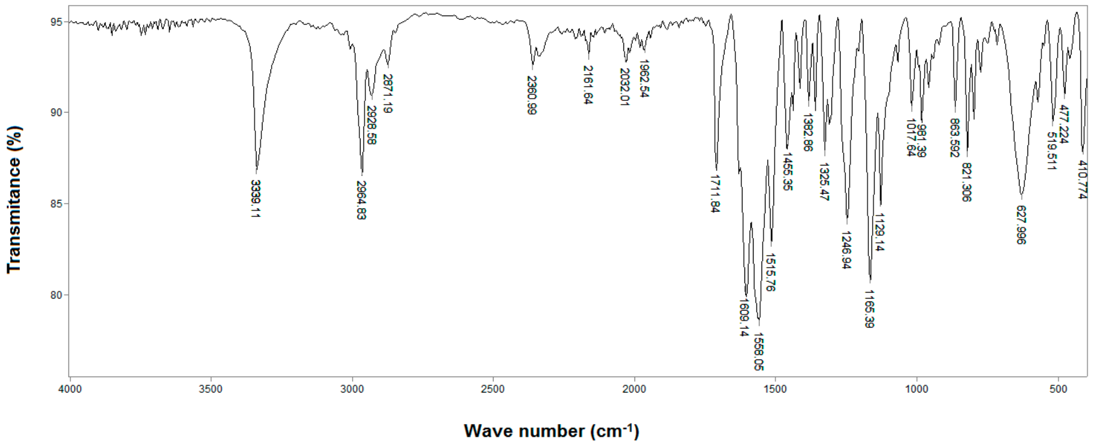

Figure S12 – Infrared spectrum (ATR) of **4l**.

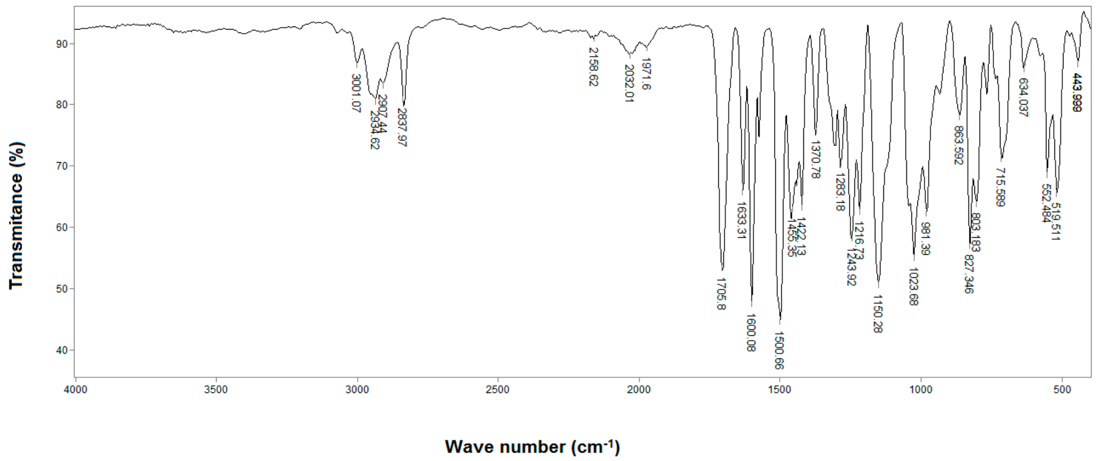

Figure S13 – Infrared spectrum (ATR) of **4m**.

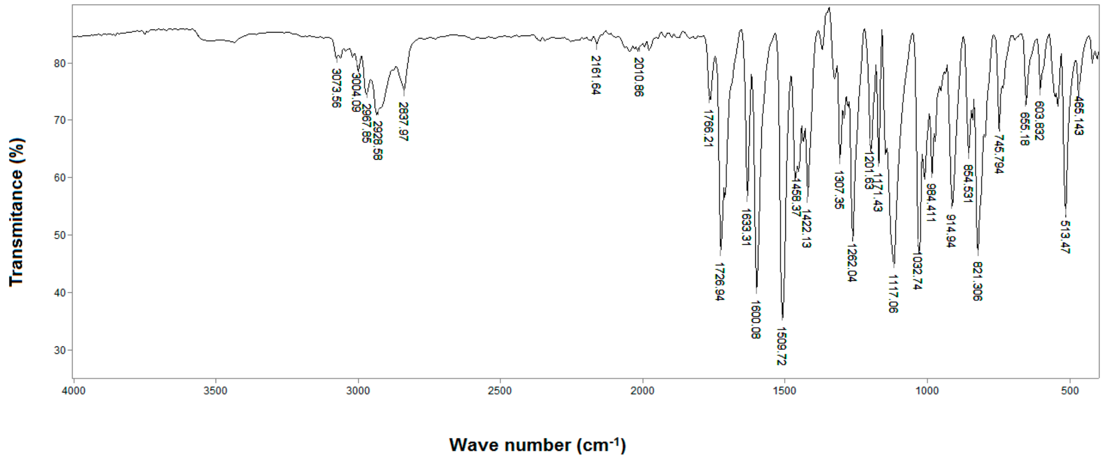

Figure S14 – Infrared spectrum (ATR) of **4n**.

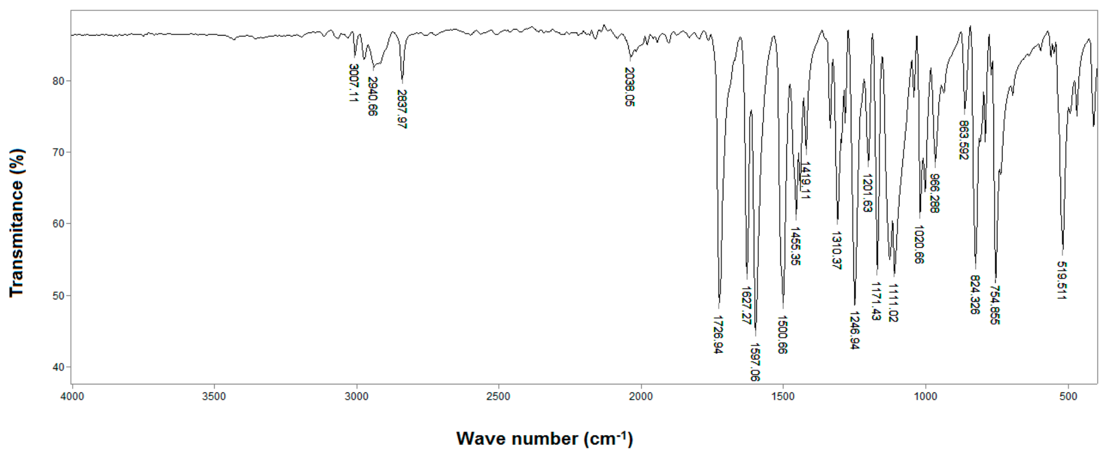

Figure S15 – Infrared spectrum (ATR) of **4o**.

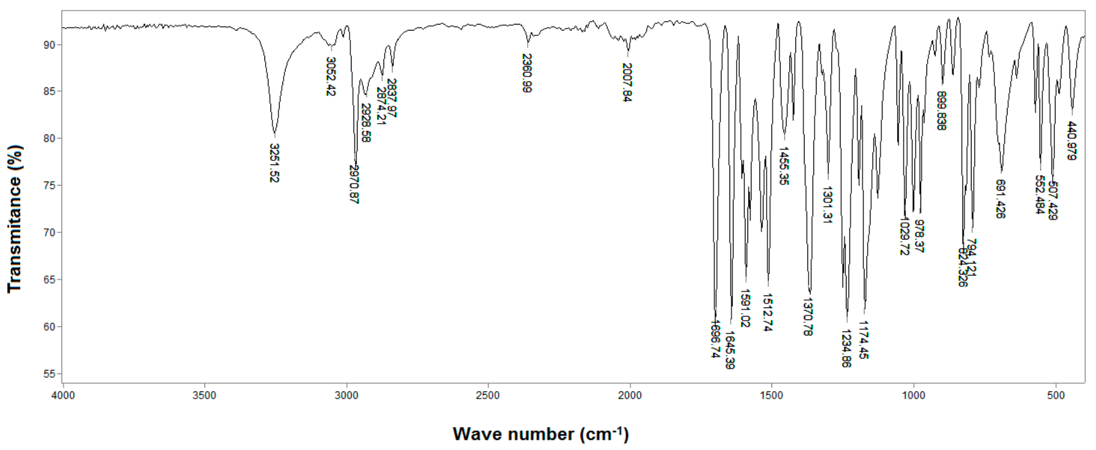

Figure S16 – Infrared spectrum (ATR) of **4p**.

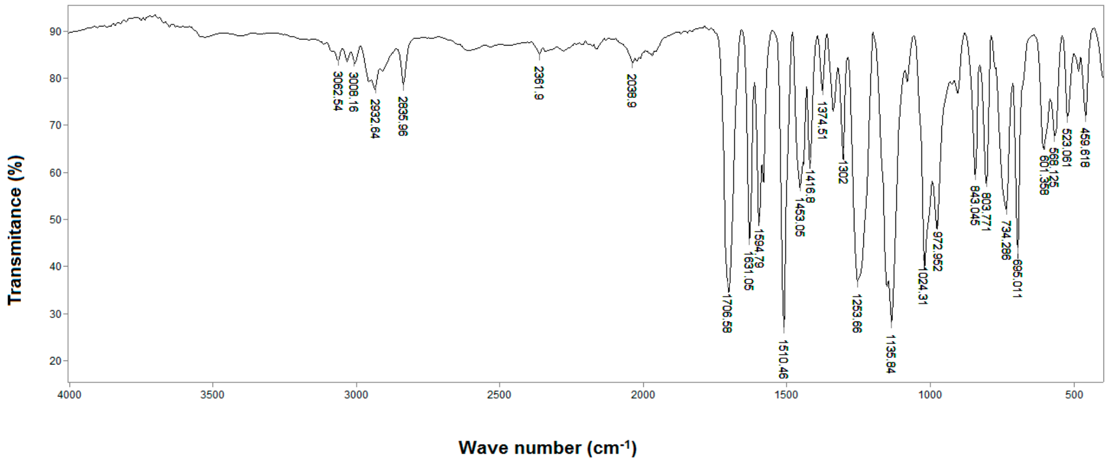

Figure S17 – Infrared spectrum (ATR) of **5a**.

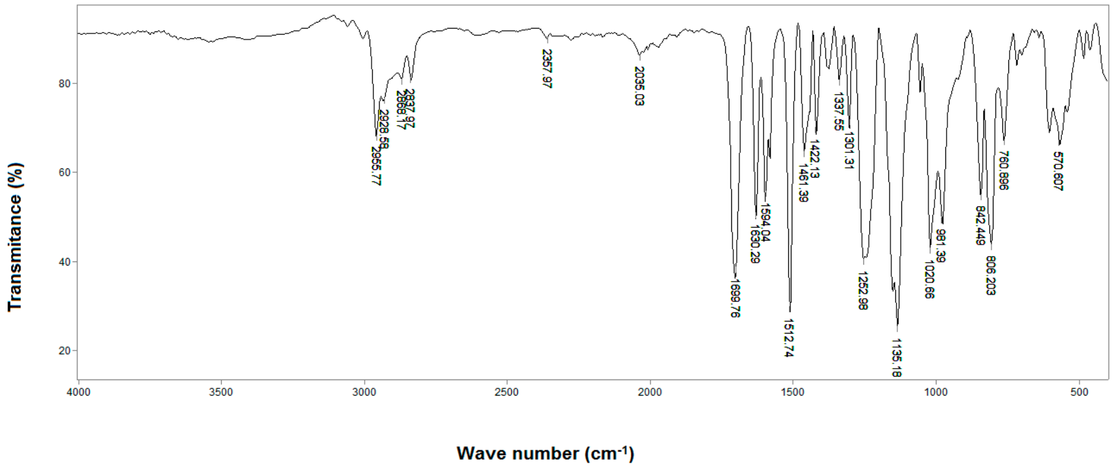

Figure S18 – Infrared spectrum (ATR) of **5b**.

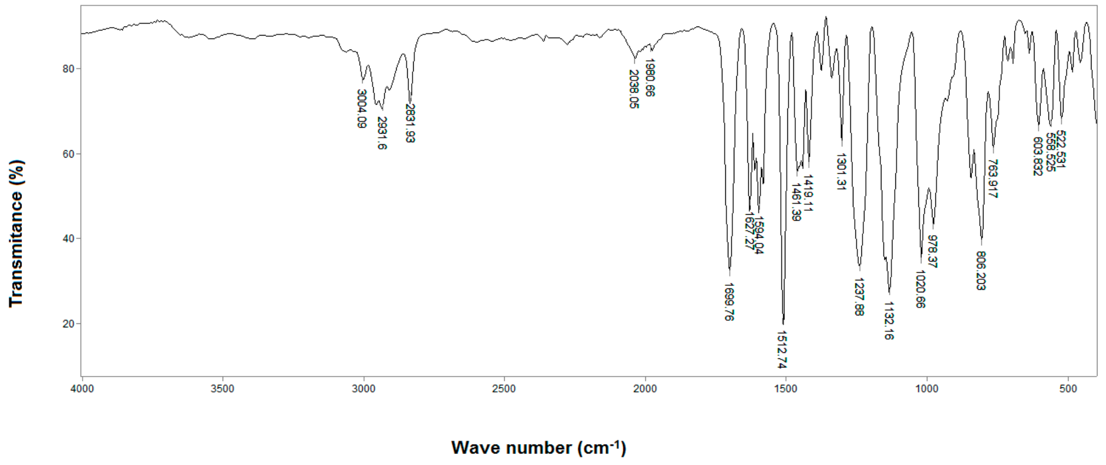

Figure S19 – Infrared spectrum (ATR) of **5c**.

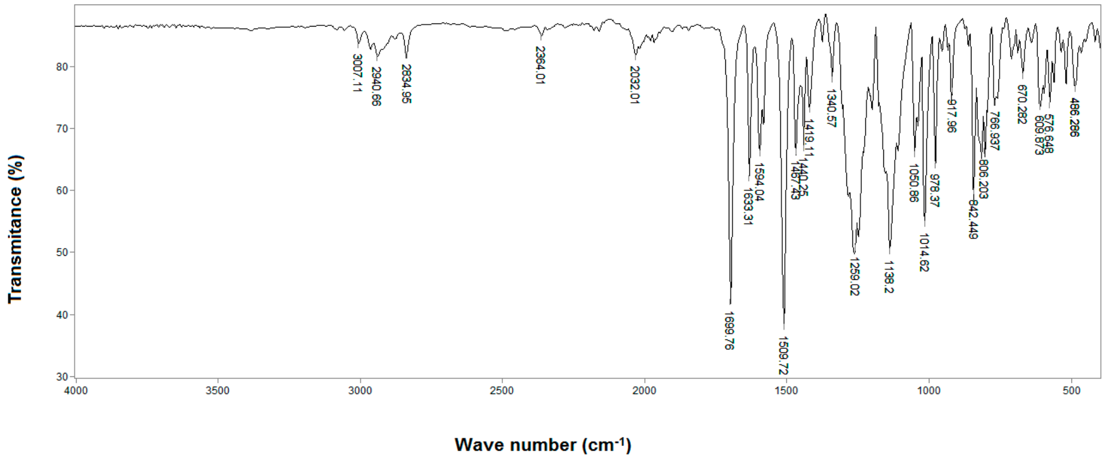

Figure S20 – Infrared spectrum (ATR) of **5d**.

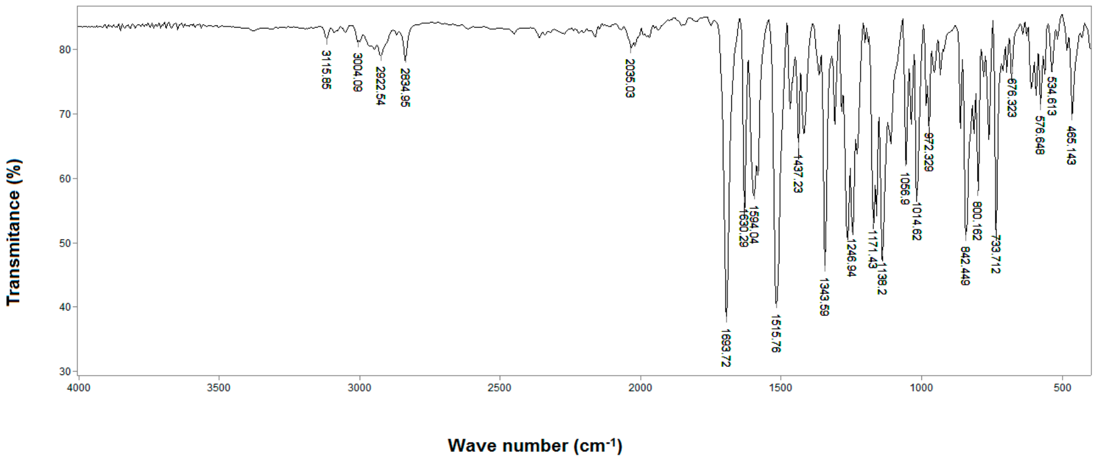

Figure S21 – Infrared spectrum (ATR) of **5e**.

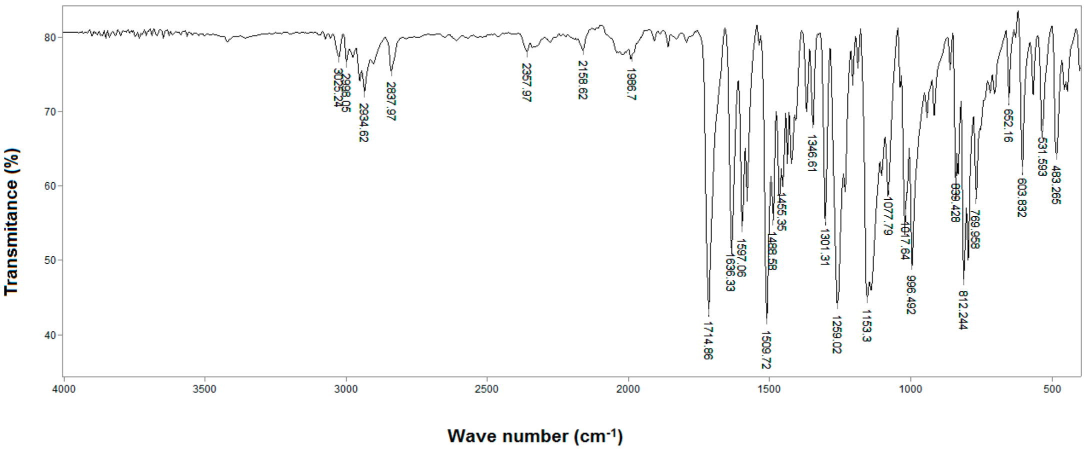

Figure S22 – Infrared spectrum (ATR) of **5f**.

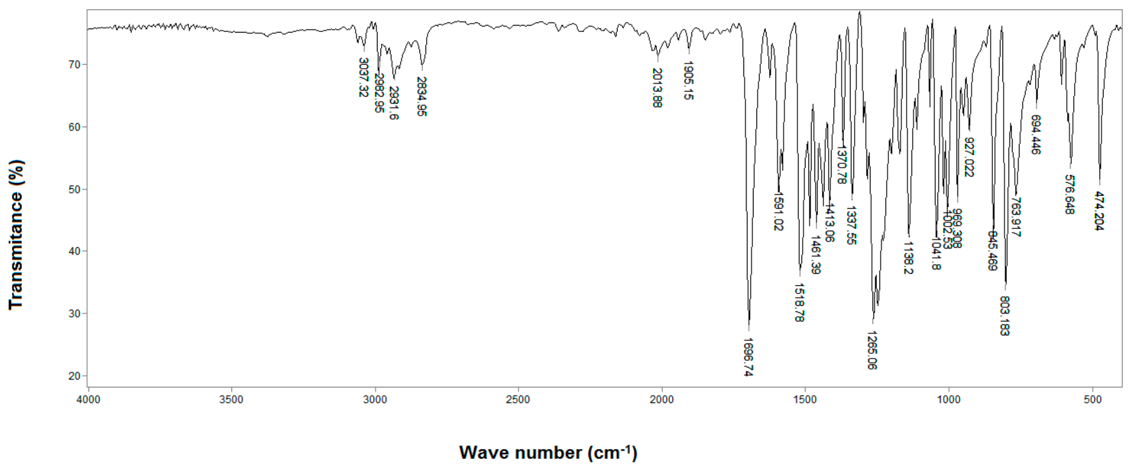

Figure S23 – Infrared spectrum (ATR) of **5g**.

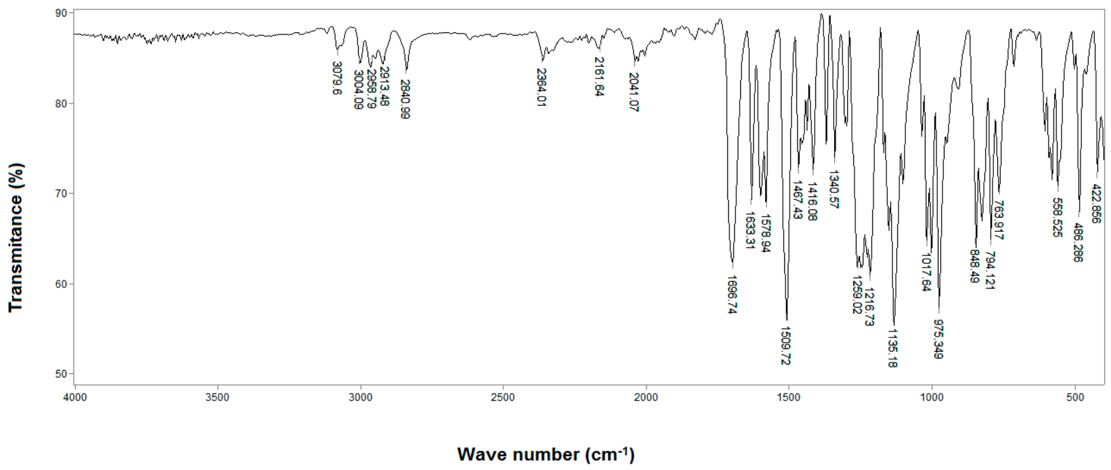

Figure S24 – Infrared spectrum (ATR) of **5h**.

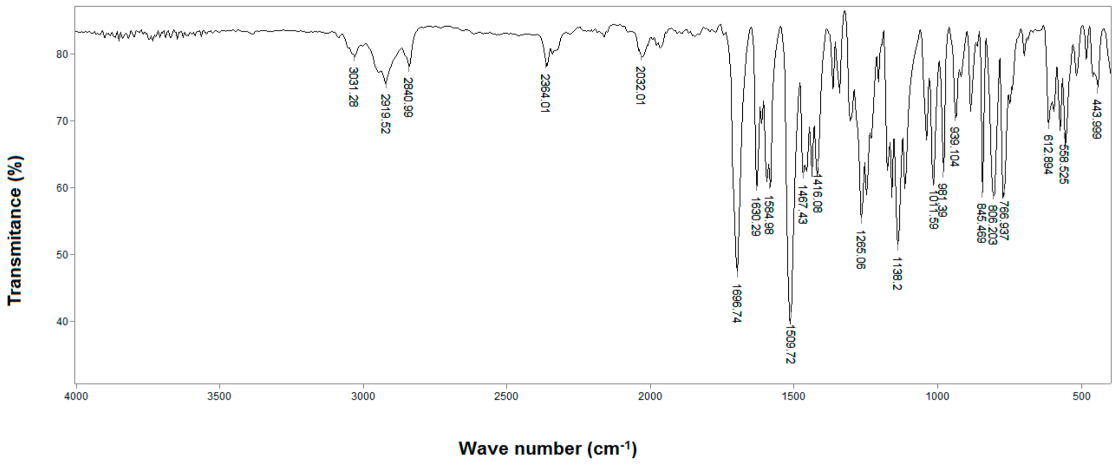

Figure S25 – Infrared spectrum (ATR) of **5i**.

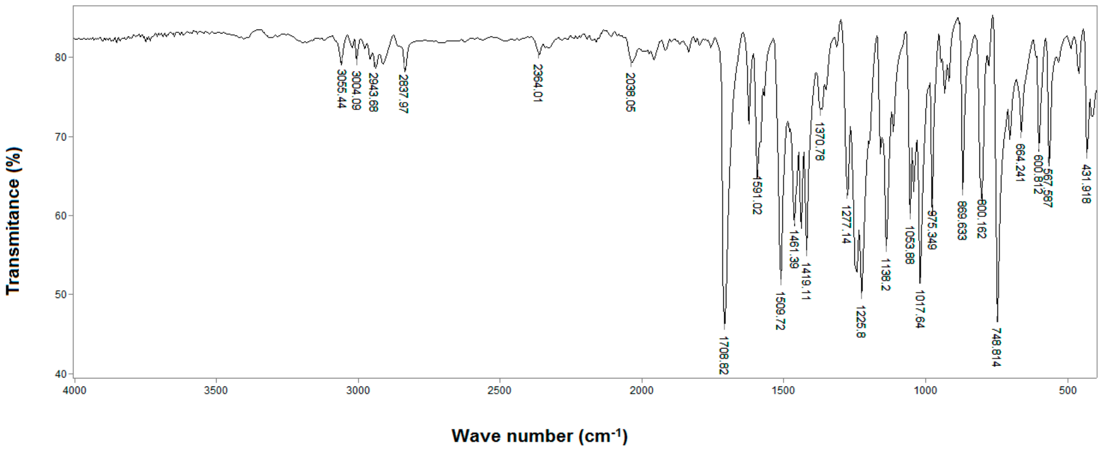

Figure S26 – Infrared spectrum (ATR) of **5j**.

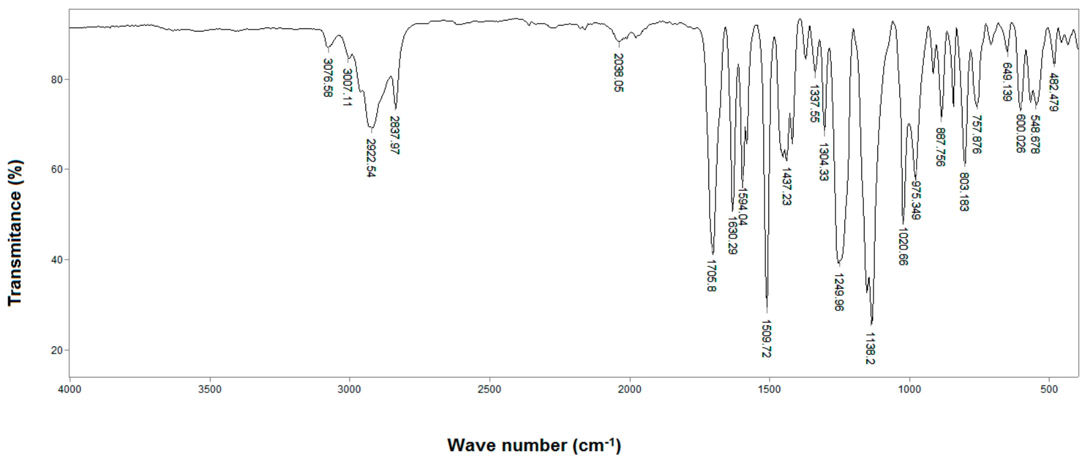

Figure S27 – Infrared spectrum (ATR) of **5k**.

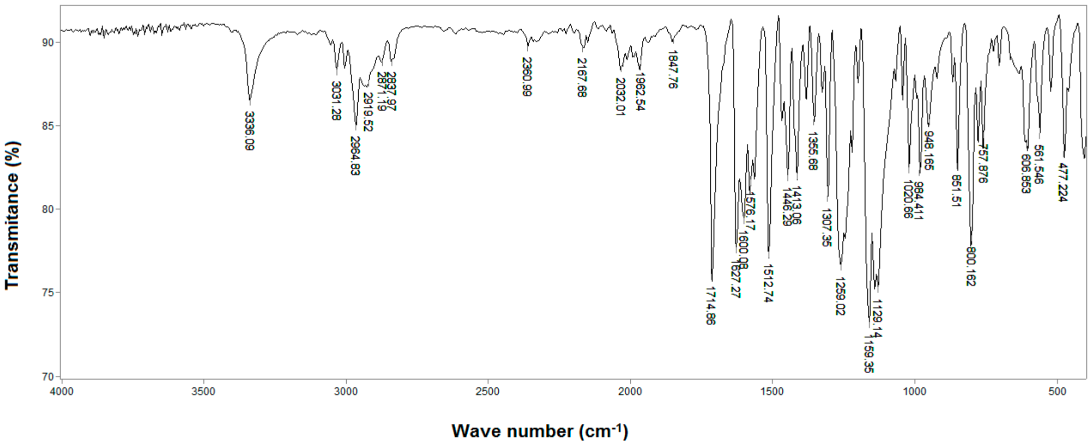

Figure S28 – Infrared spectrum (ATR) of **5l**.

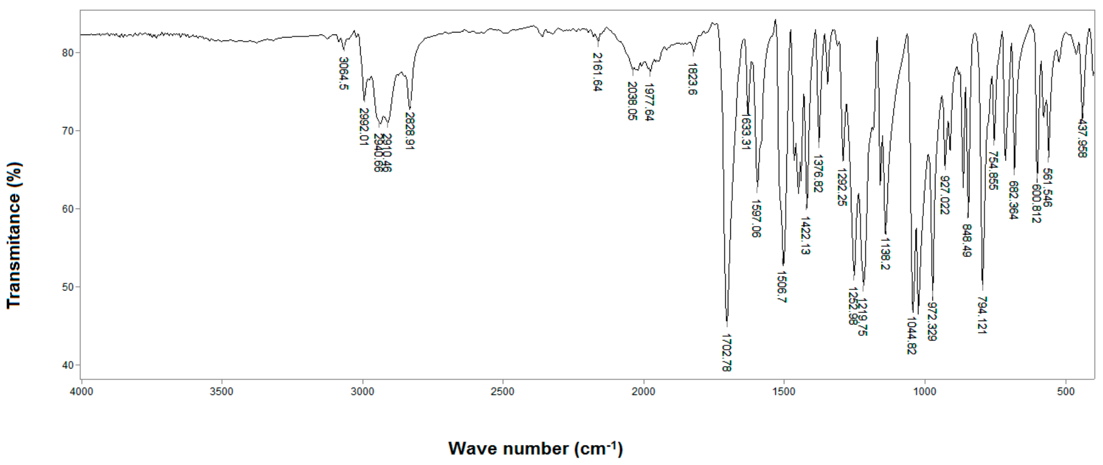

Figure S29 – Infrared spectrum (ATR) of **5m**.

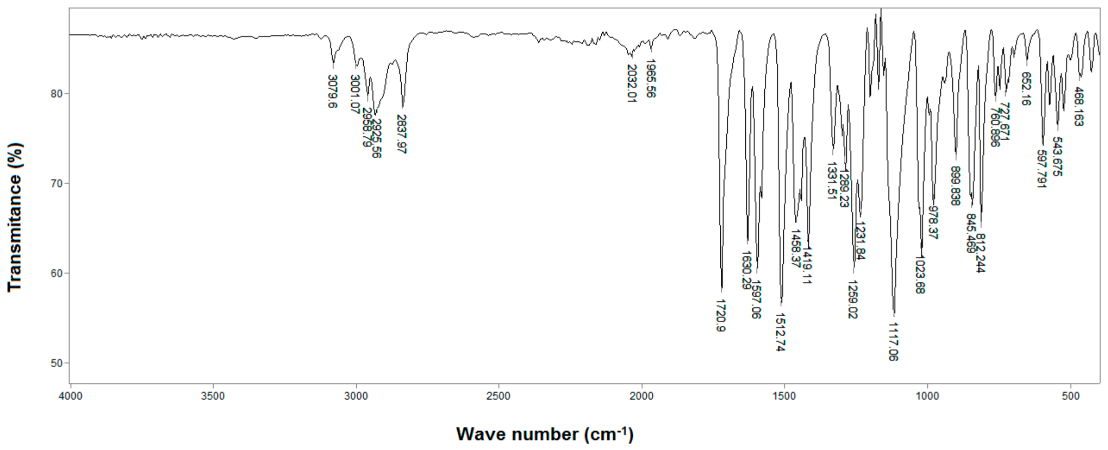

Figure S30 – Infrared spectrum (ATR) of **5n**.

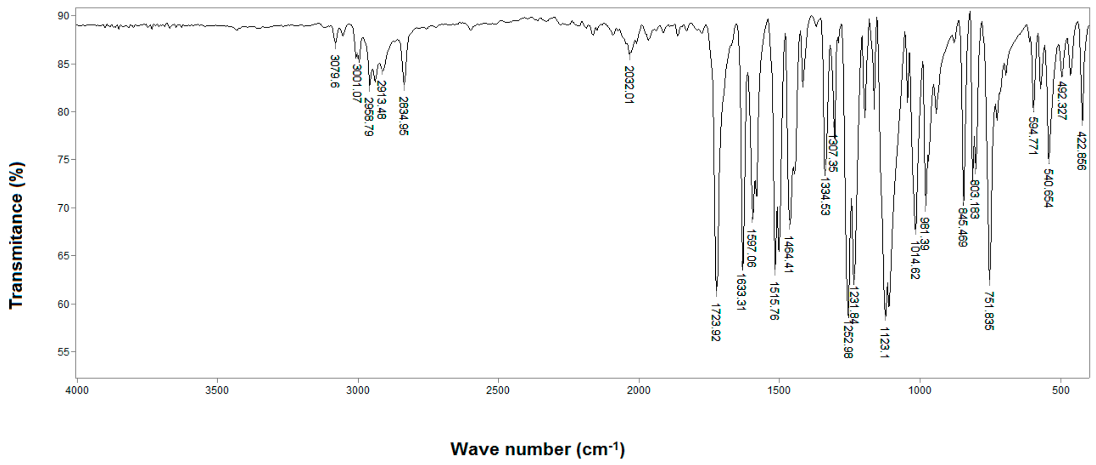

Figure S31 – Infrared spectrum (ATR) of **5o**.

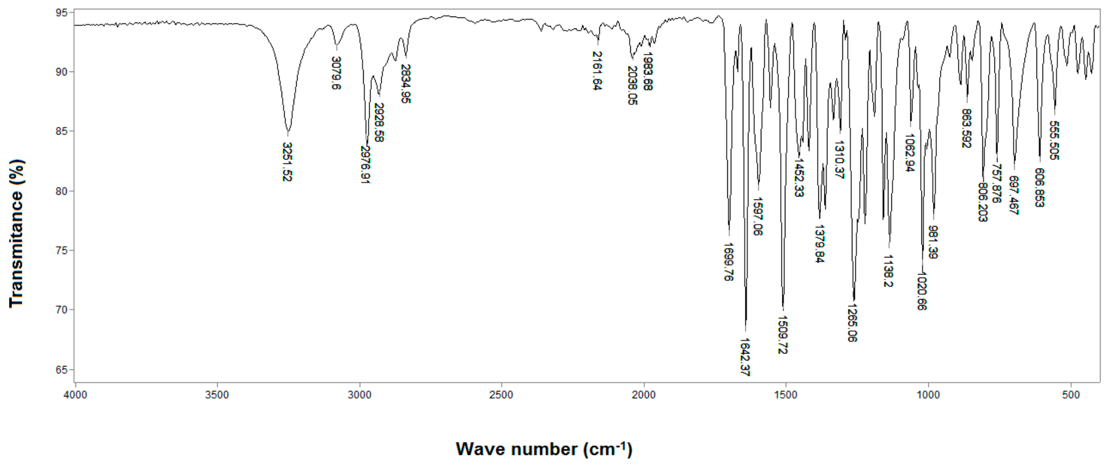

Figure S32 – Infrared spectrum (ATR) of **5p**.

^{1}H NUCLEAR MAGNETIC RESONANCE (NMR) SPECTRA OF COMPOUNDS 4a–4p AND 5a–5p

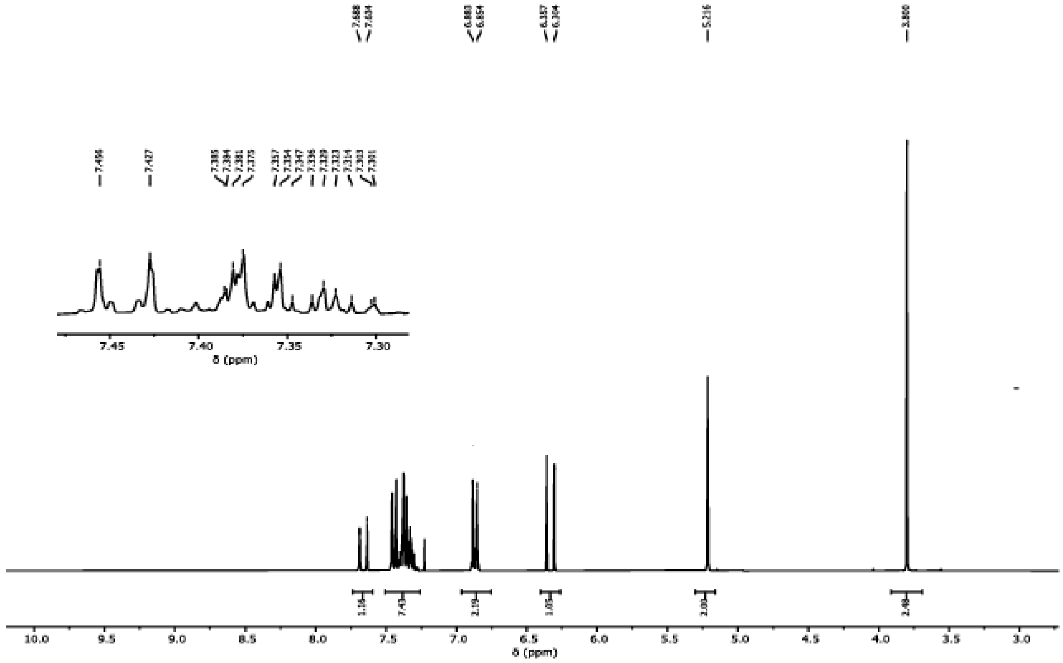

Figure S33 – ^{1}H NMR spectrum (300 MHz, $CDCl_3$) of **4a**.

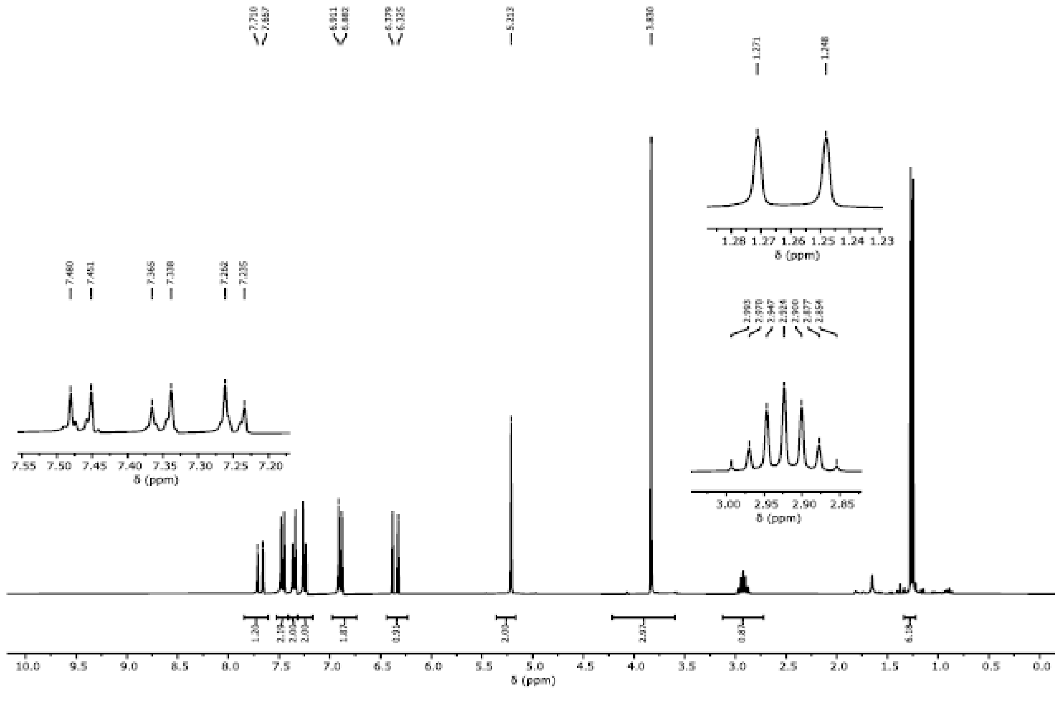

Figure S34 – ^{1}H NMR spectrum (300 MHz, $CDCl_3$) of **4b**.

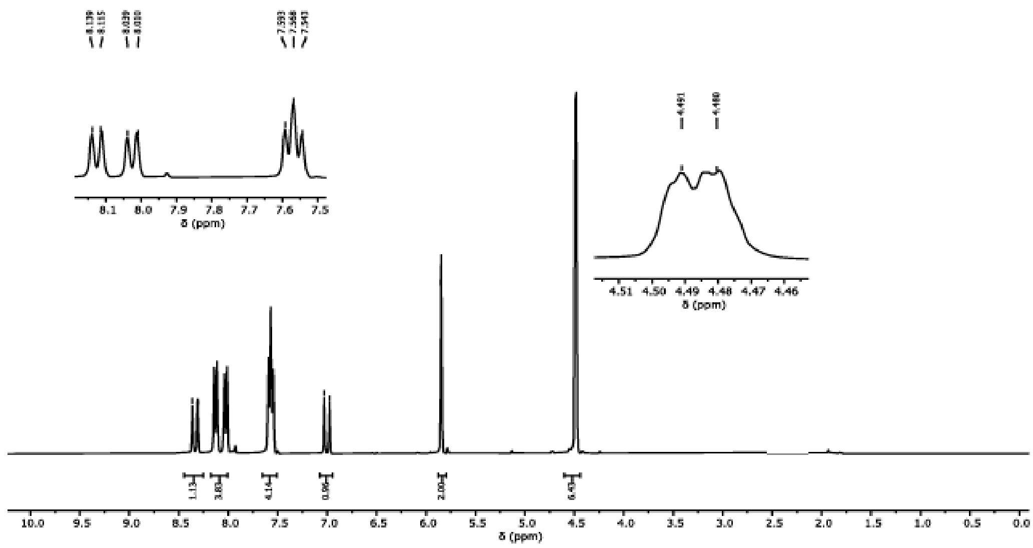

Figure S35 – ^{1}H NMR spectrum (300 MHz, $CDCl_3$) of **4c**.

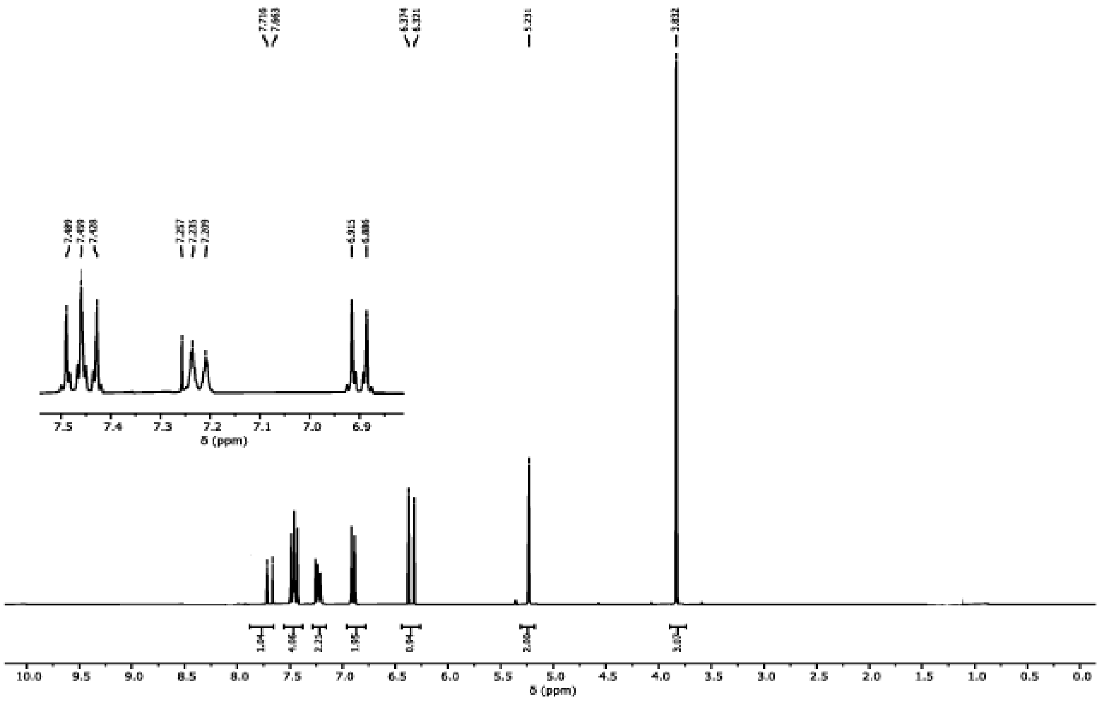

Figure S36 – ^{1}H NMR spectrum (300 MHz, $CDCl_3$) of **4d**.

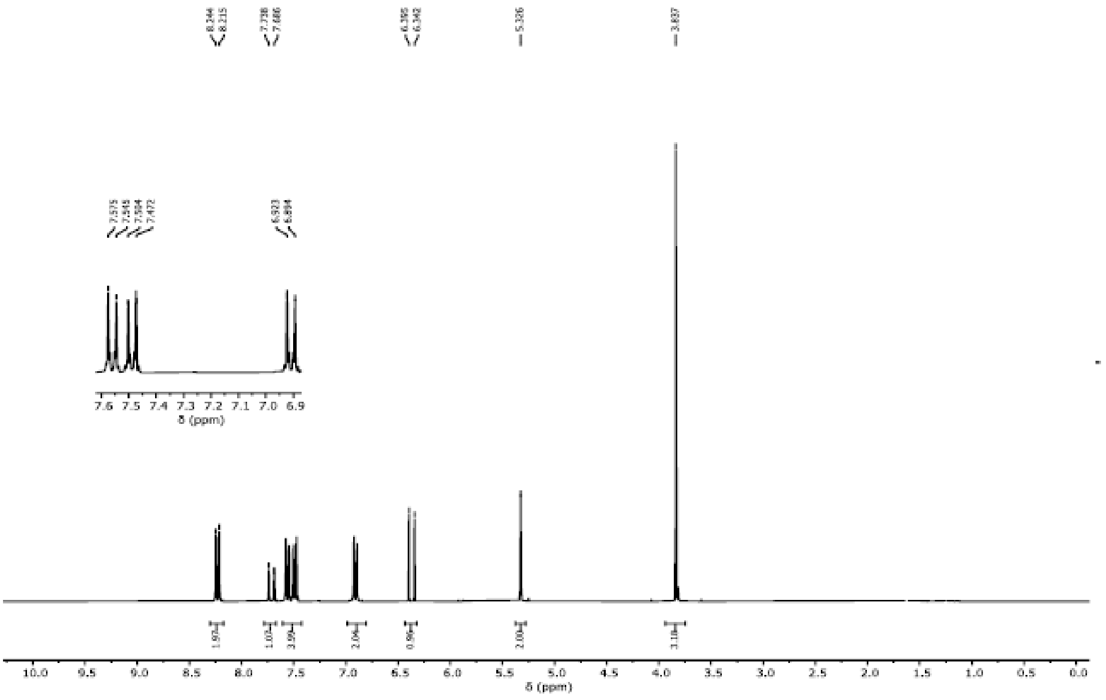

Figure S37 – ^{1}H NMR spectrum (300 MHz, $CDCl_3$) of **4e**.

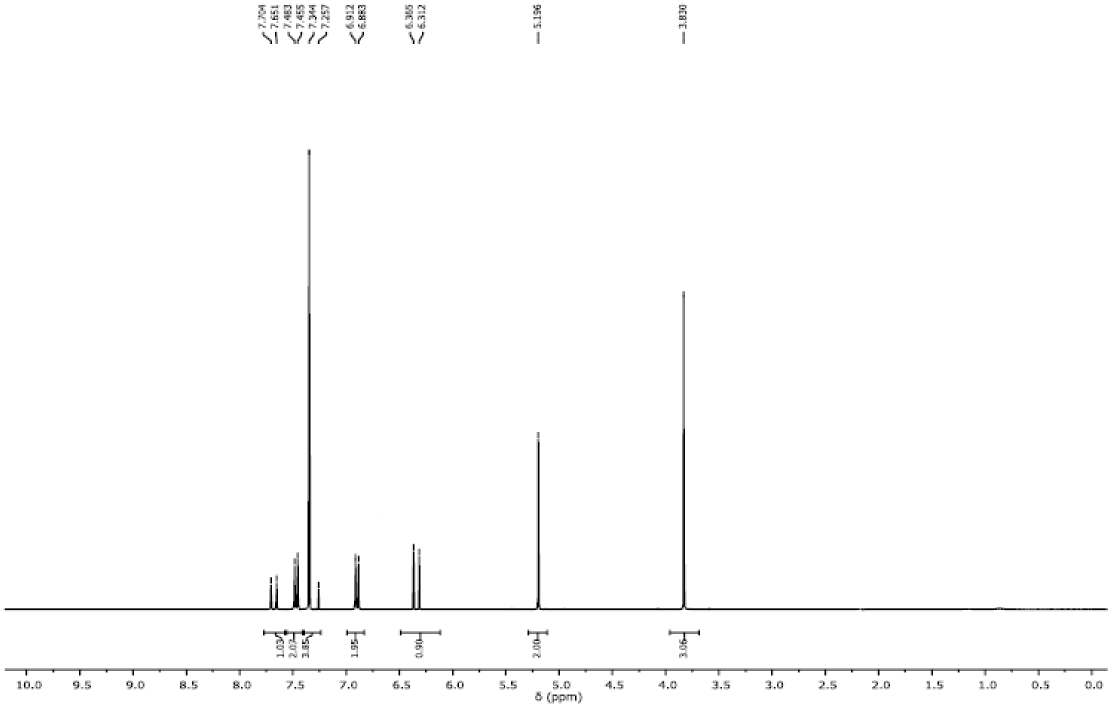

Figure S38 – ^{1}H NMR spectrum (300 MHz, $CDCl_3$) of **4f**.

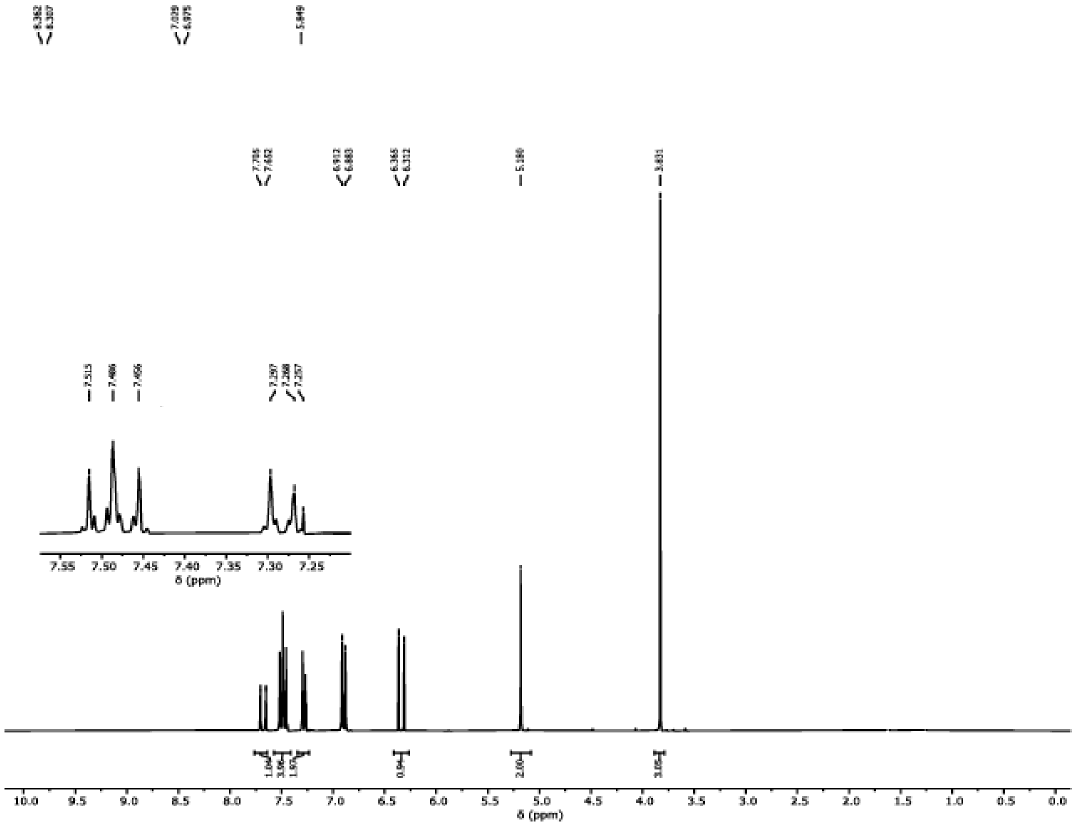

Figure S39 – ^{1}H NMR spectrum (300 MHz, $CDCl_3$) of **4g**.

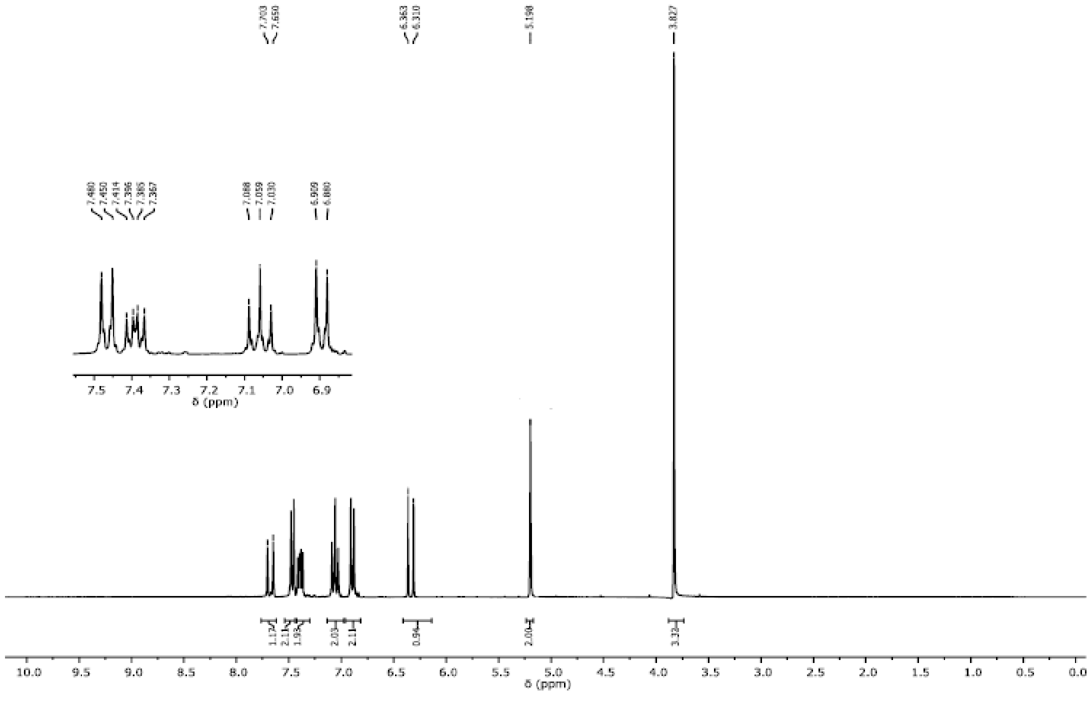

Figure S40 – ^{1}H NMR spectrum (300 MHz, $CDCl_3$) of **4h**.

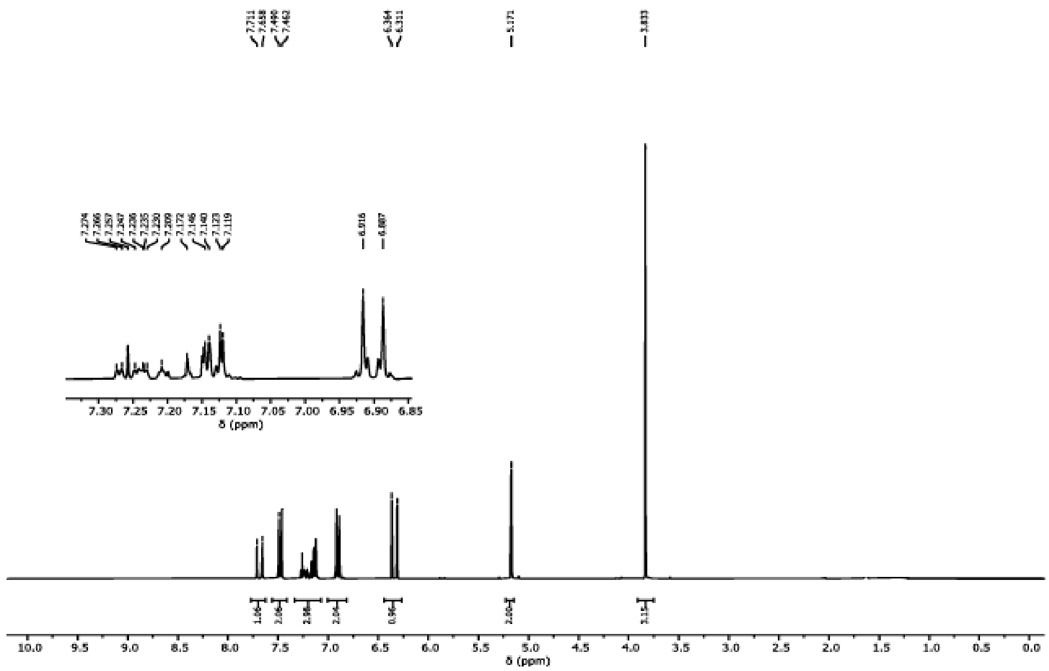

Figure S41 – ^{1}H NMR spectrum (300 MHz, $CDCl_3$) of **4i**.

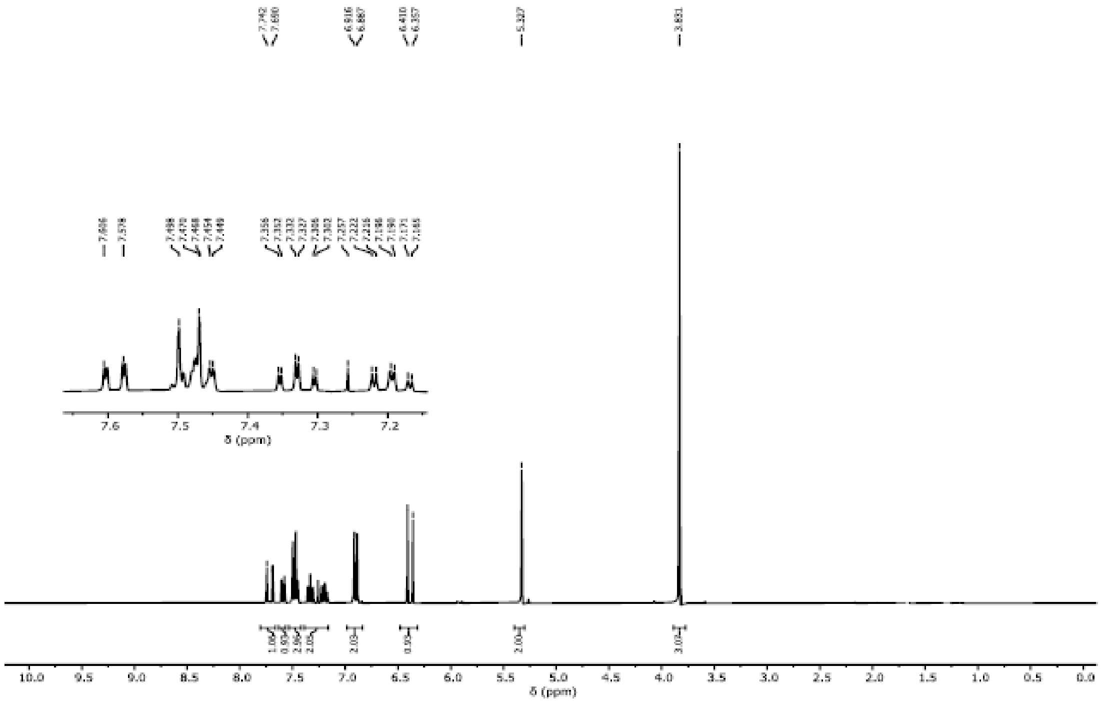

Figure S42 – ^{1}H NMR spectrum (300 MHz, $CDCl_3$) of **4j**.

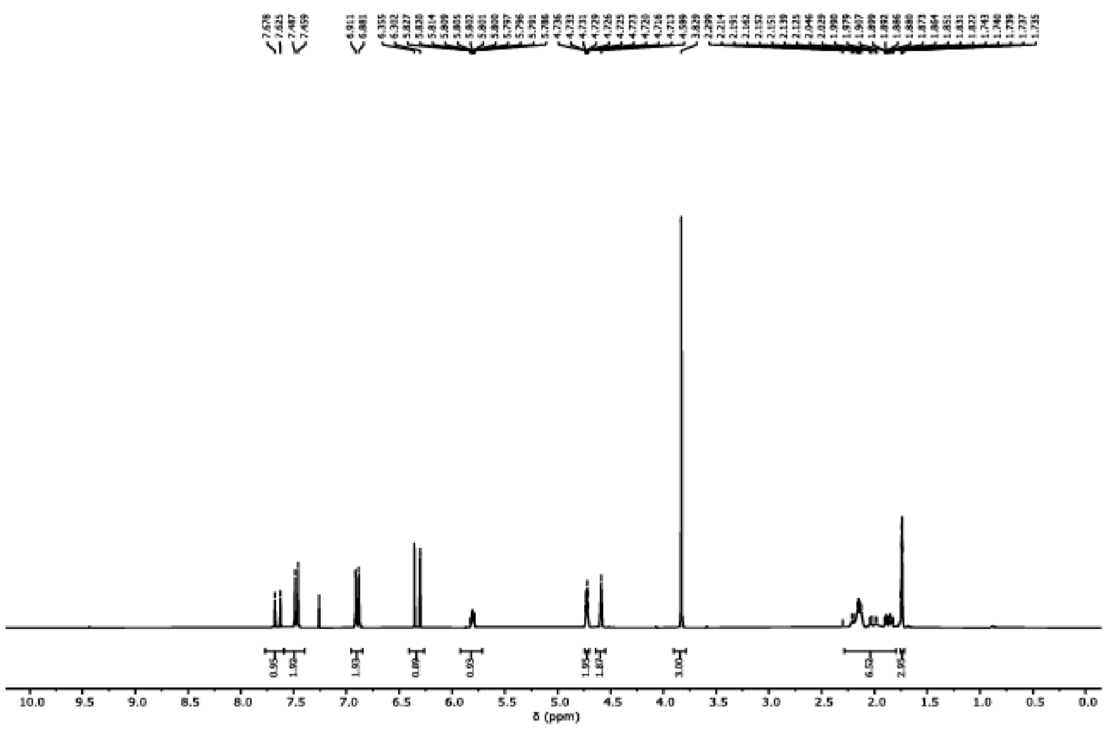

Figure S43 – ^{1}H NMR spectrum (300 MHz, $CDCl_3$) of **4k**.

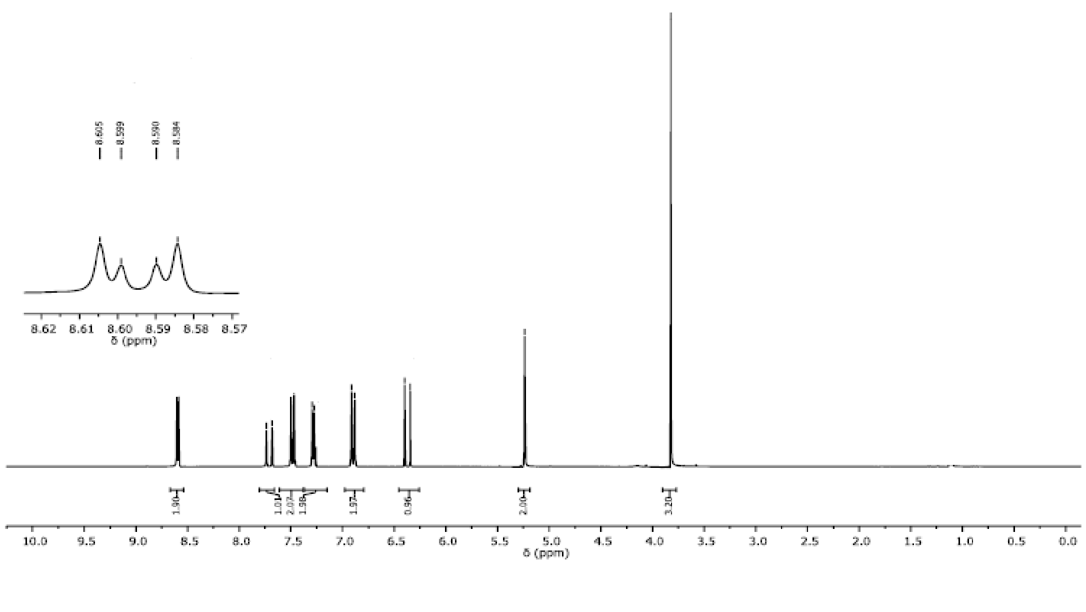

Figure S44 – ^{1}H NMR spectrum (300 MHz, $CDCl_3$) of **4l**.

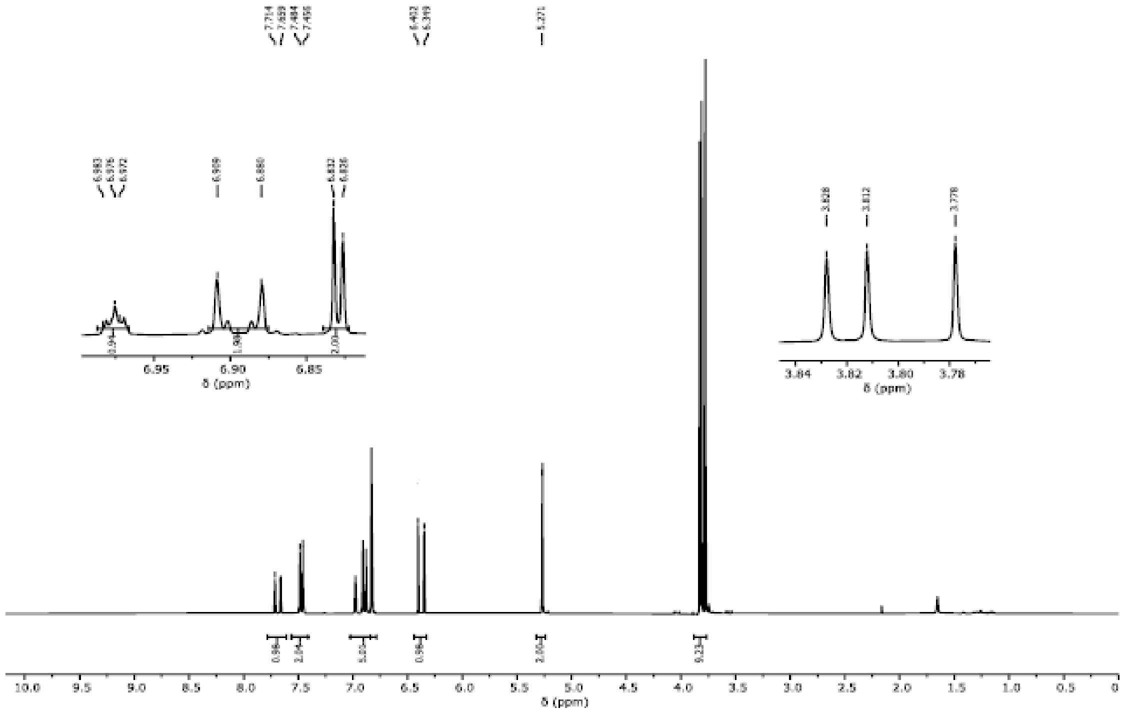

Figure S45 – ^{1}H NMR spectrum (300 MHz, $CDCl_3$) of **4m**.

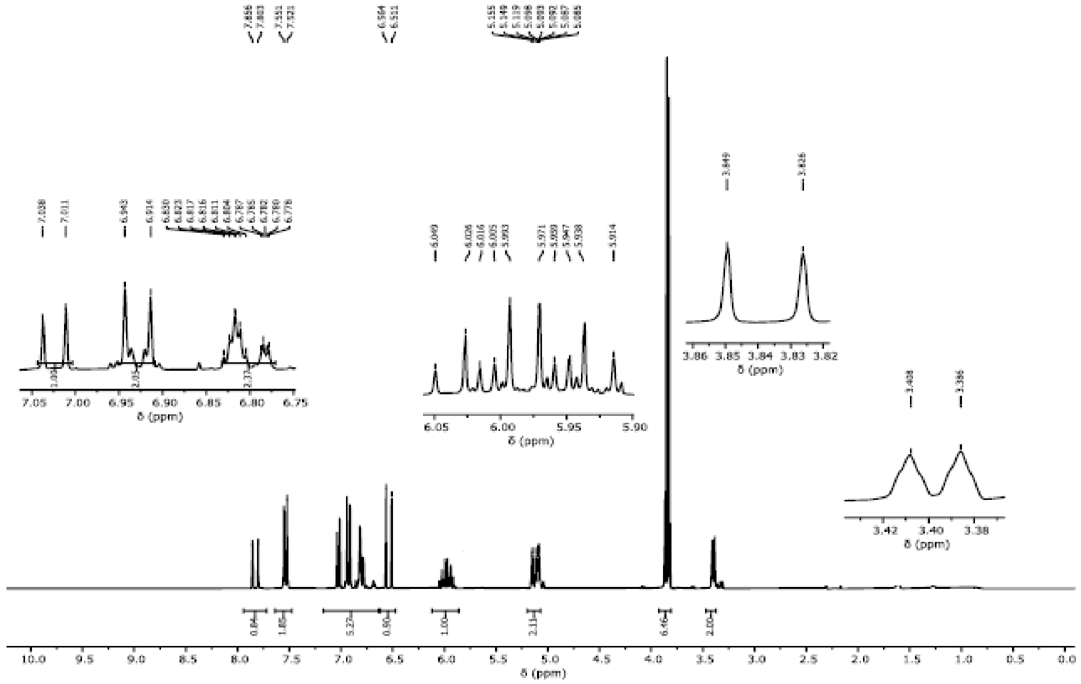

Figure S46 – ^{1}H NMR spectrum (300 MHz, $CDCl_3$) of **4n**.

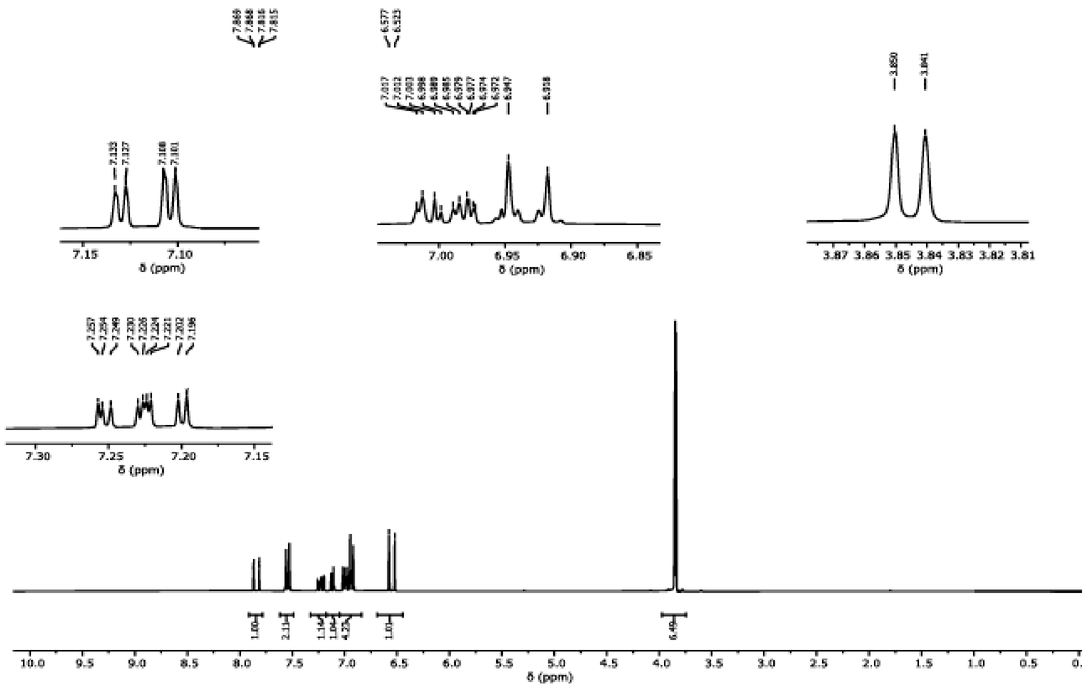

Figure S47 – ^{1}H NMR spectrum (300 MHz, $CDCl_3$) of **4o**.

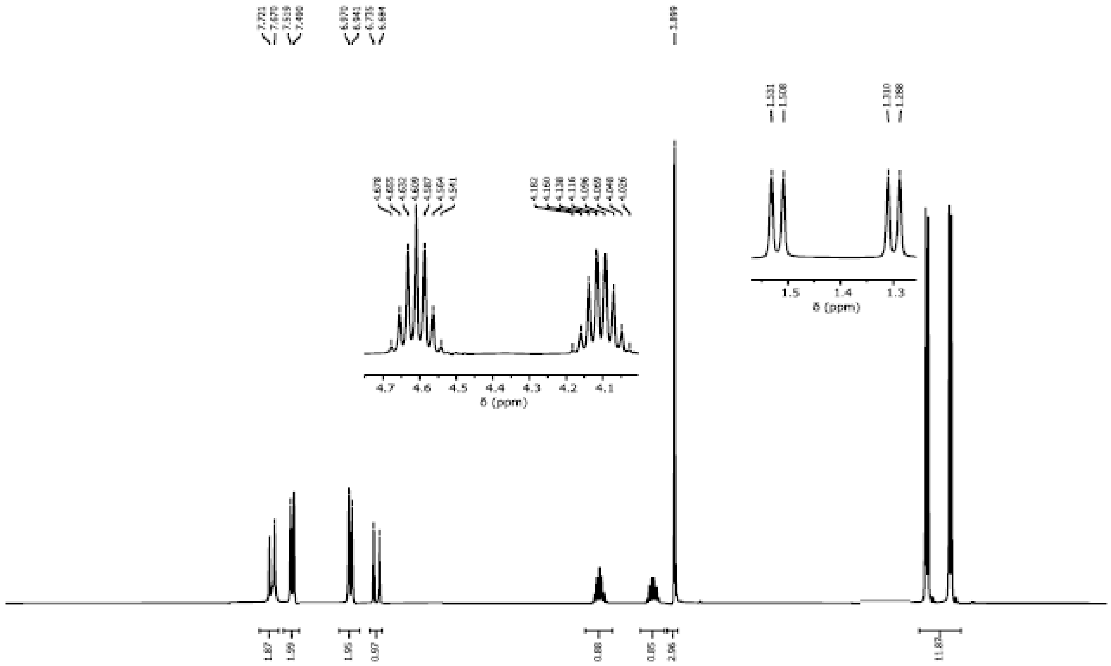

Figure S48 – ^{1}H NMR spectrum (300 MHz, $CDCl_3$) of **4p**.

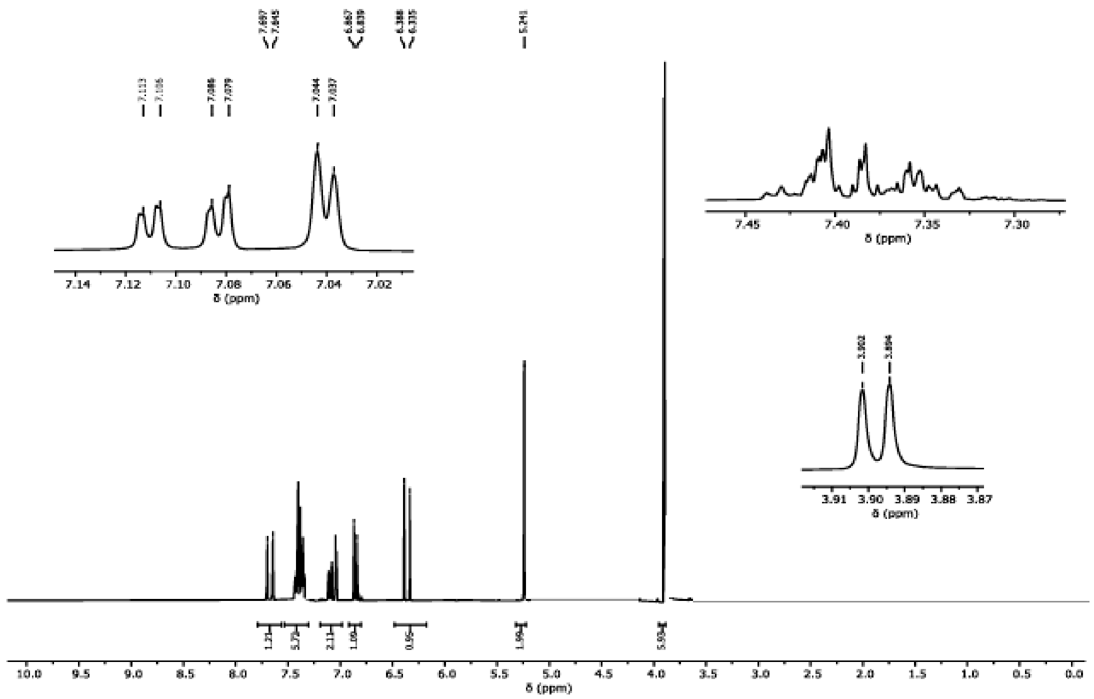

Figure S49 – ^{1}H NMR spectrum (300 MHz, $CDCl_3$) of **5a**.

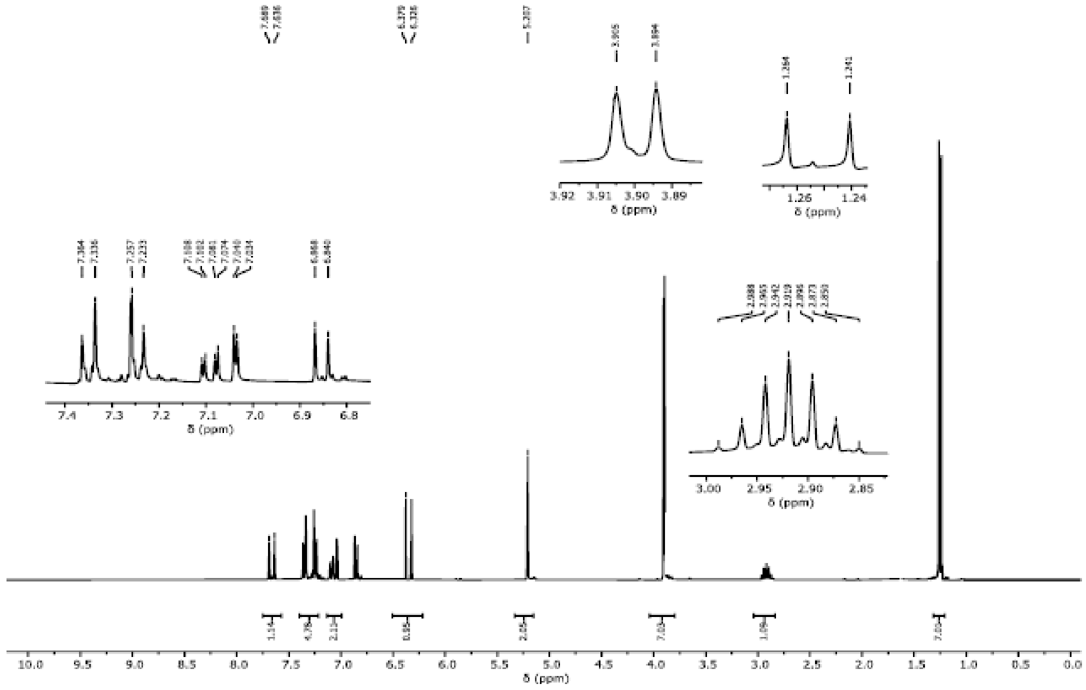

Figure S50 – ^{1}H NMR spectrum (300 MHz, $CDCl_3$) of **5b**.

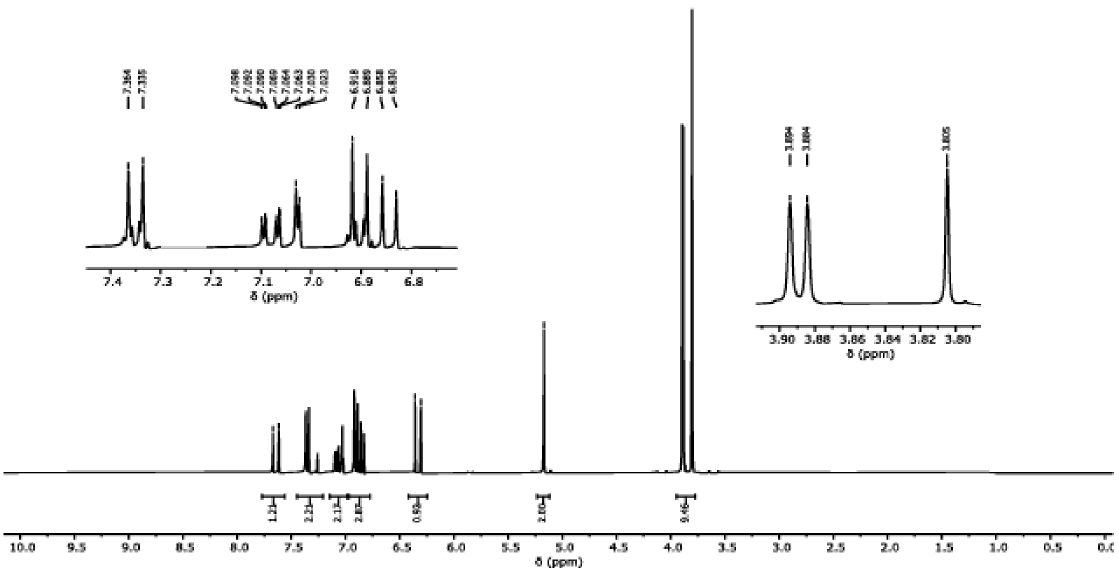

Figure S51 – ^{1}H NMR spectrum (300 MHz, $CDCl_3$) of **5c**.

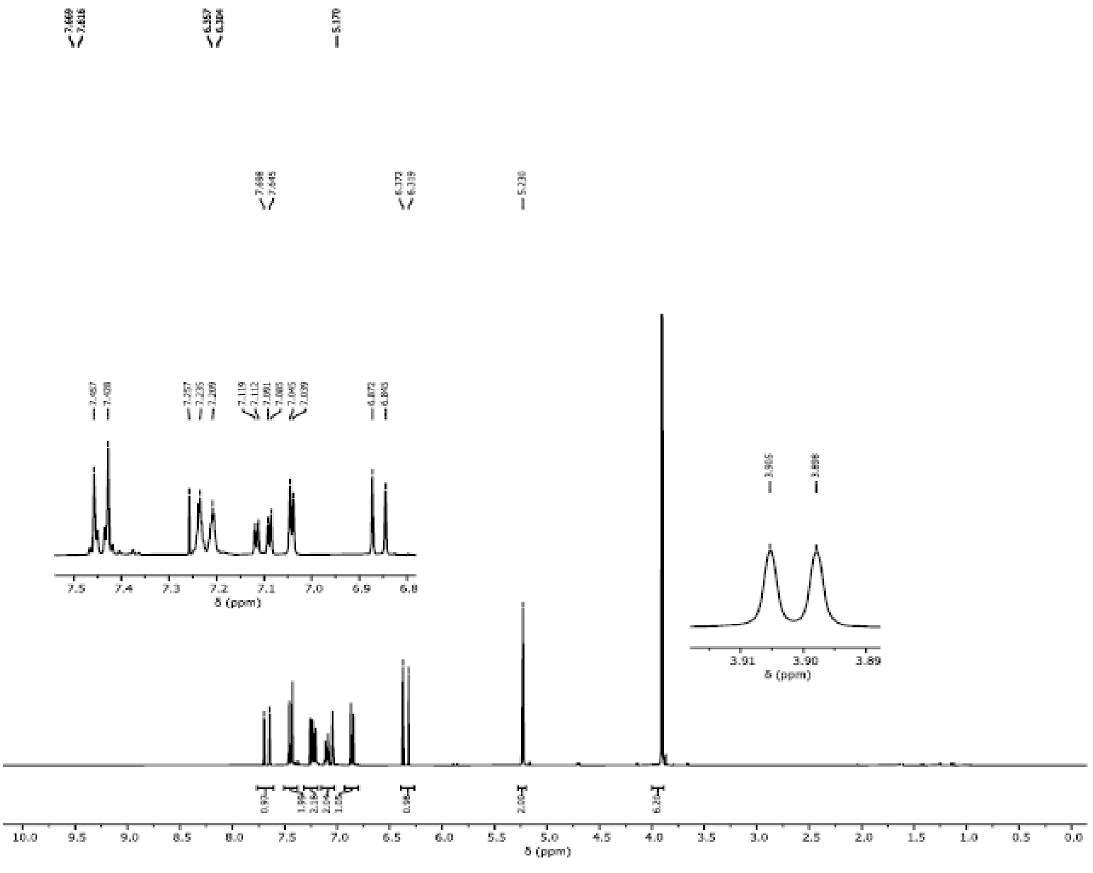

Figure S52 – ^{1}H NMR spectrum (300 MHz, $CDCl_3$) of **5d**.

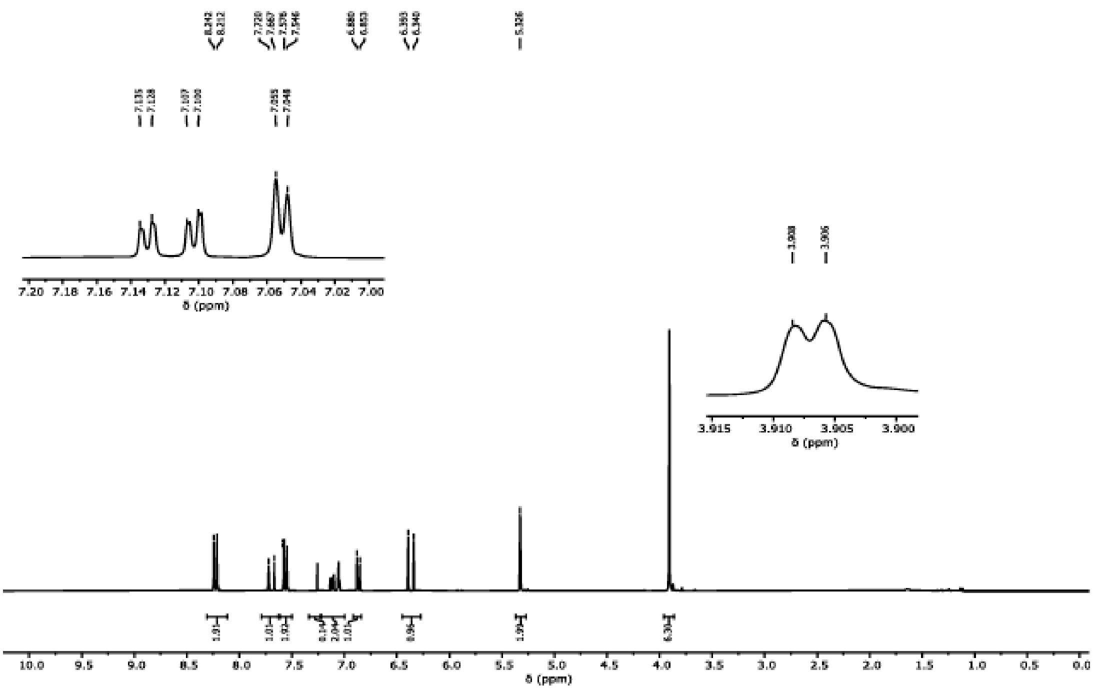

Figure S53 – ^{1}H NMR spectrum (300 MHz, $CDCl_3$) of **5e**.

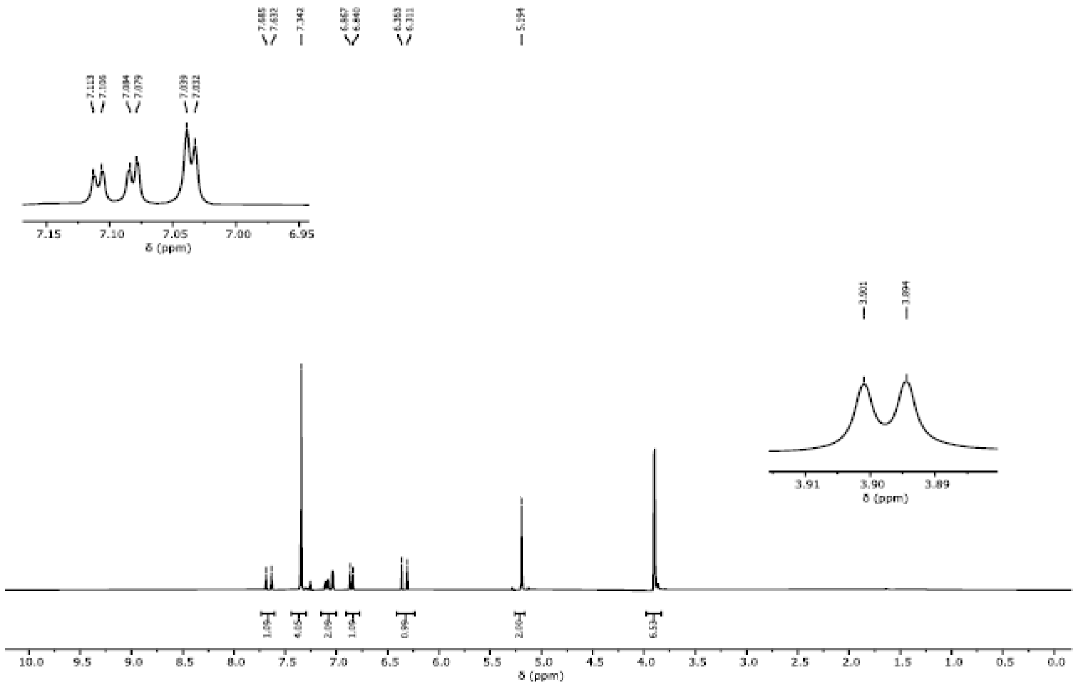

Figure S54 – ^{1}H NMR spectrum (300 MHz, $CDCl_3$) of **5f**.

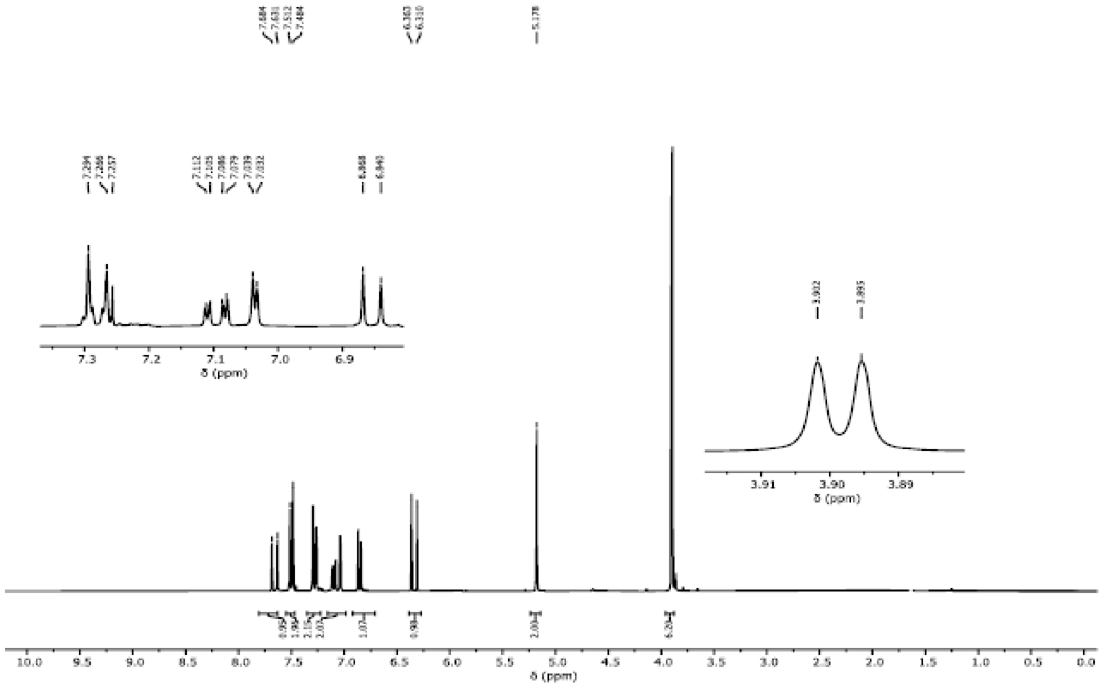

Figure S55 – ^{1}H NMR spectrum (300 MHz, $CDCl_3$) of **5g**.

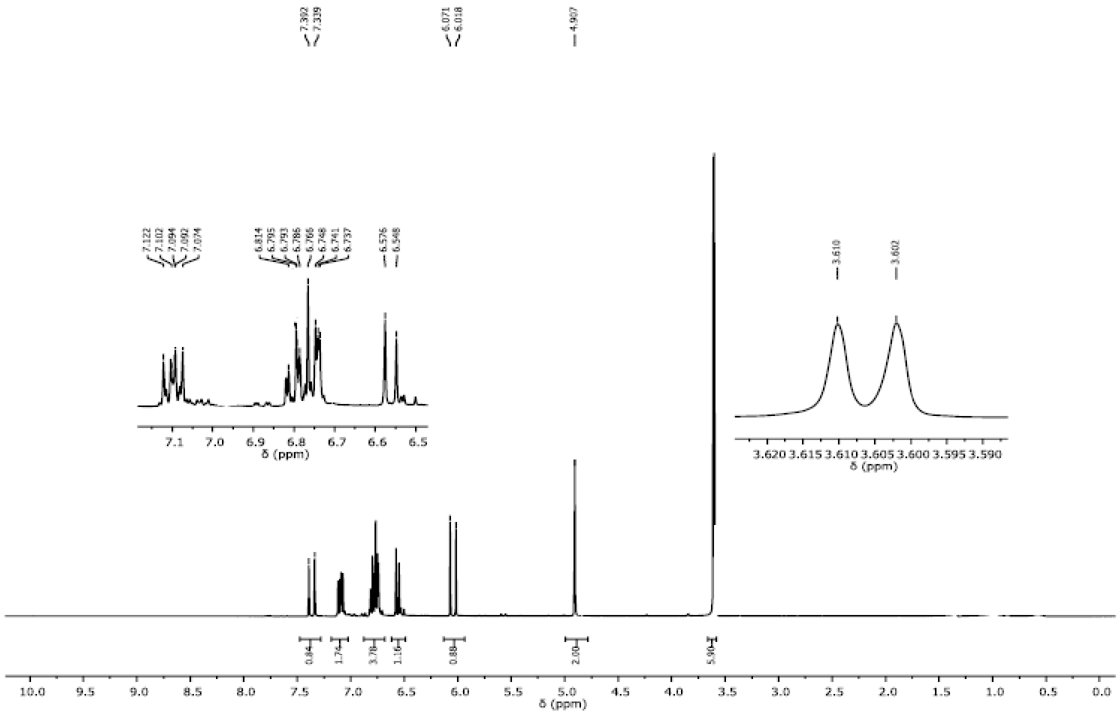

Figure S55 – ^{1}H NMR spectrum (300 MHz, $CDCl_3$) of **5h**.

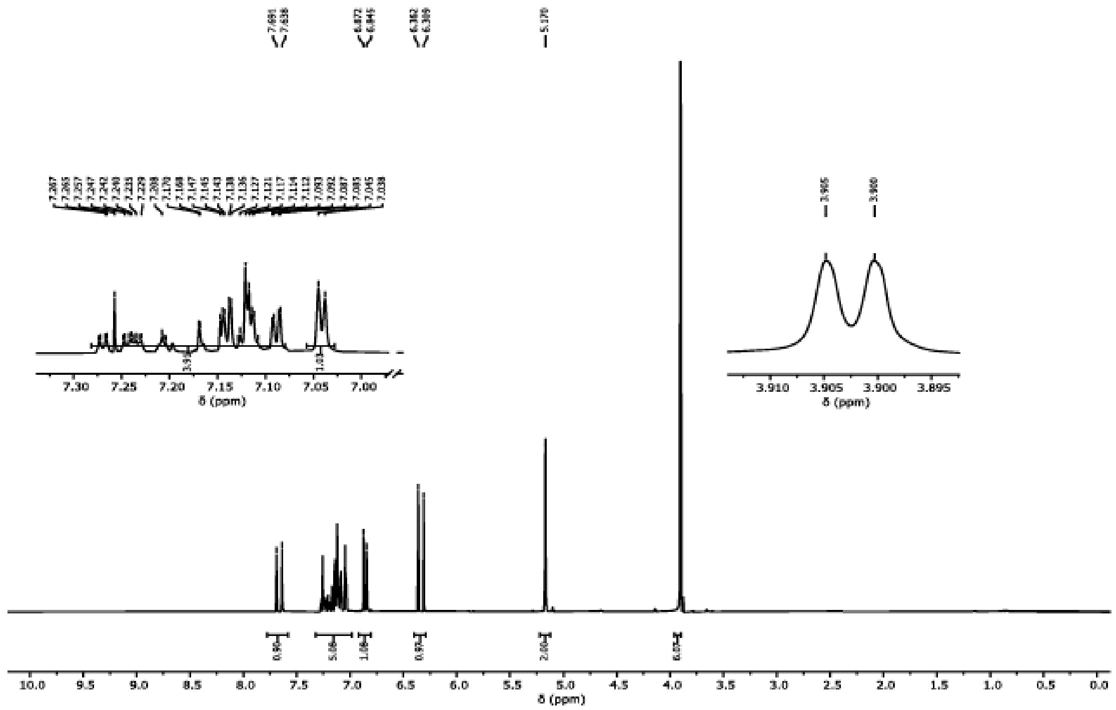

Figure S57 – ^{1}H NMR spectrum (300 MHz, $CDCl_3$) of **5i**.

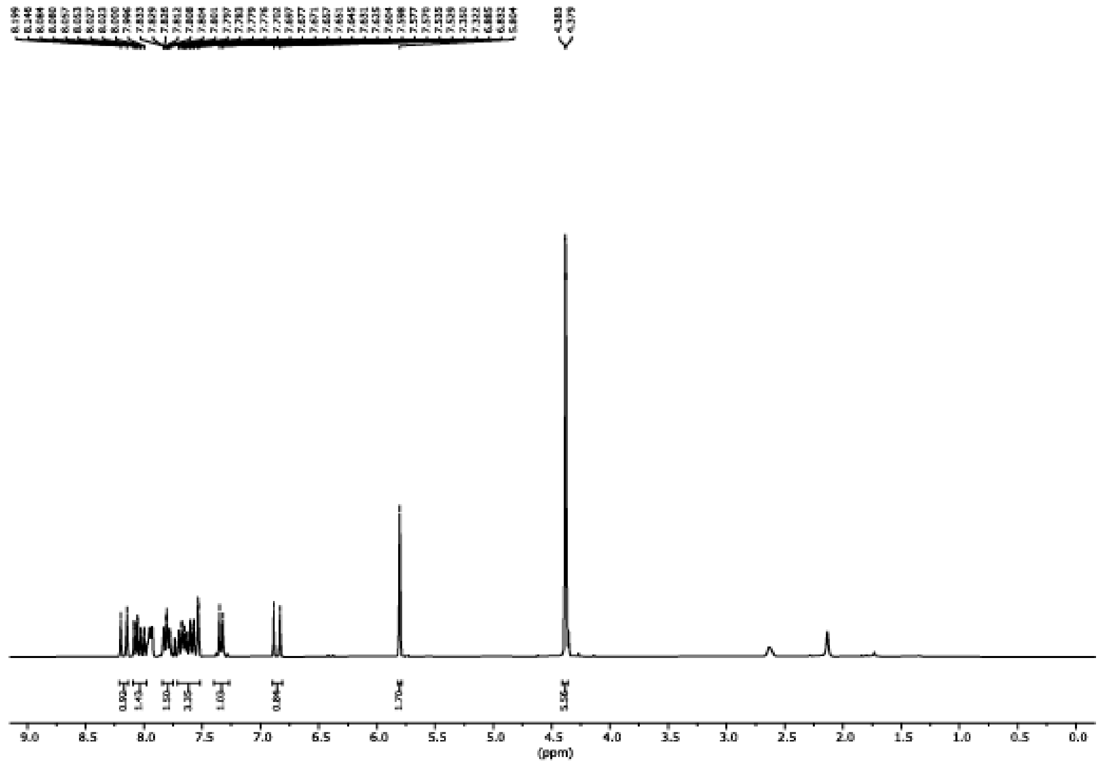

Figure S58 – ^{1}H NMR spectrum (300 MHz, $CDCl_3$) of **5j**.

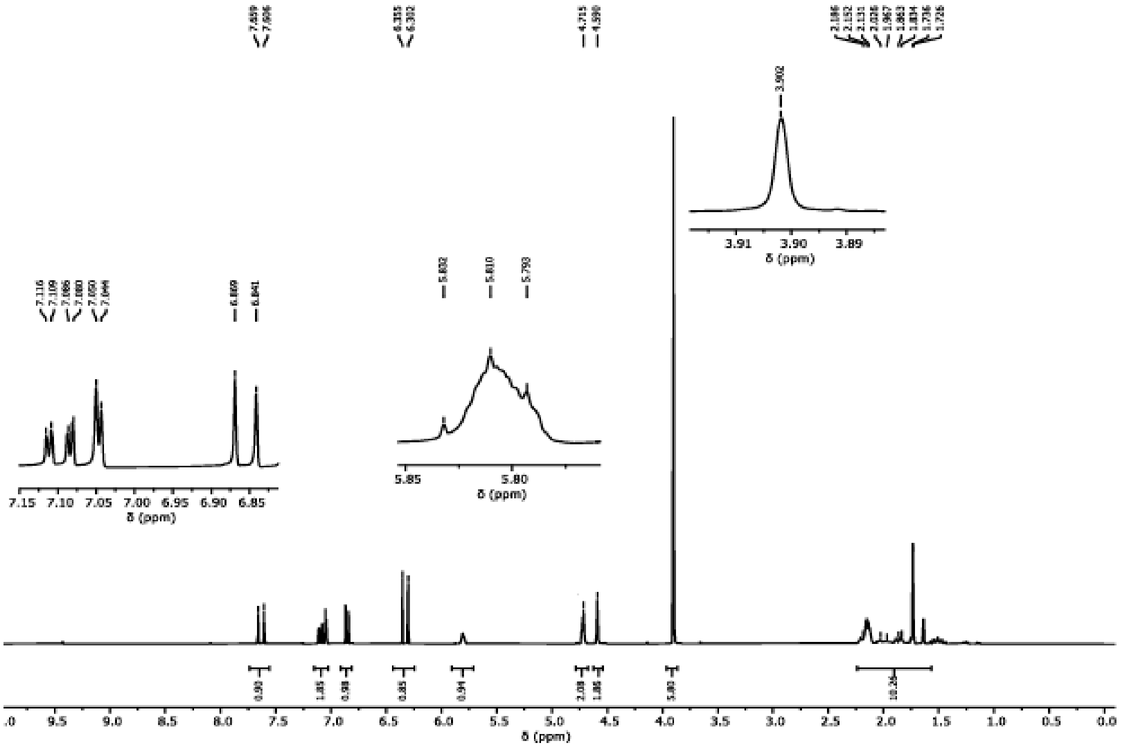

Figure S59 – ^{1}H NMR spectrum (300 MHz, $CDCl_3$) of **5k**.

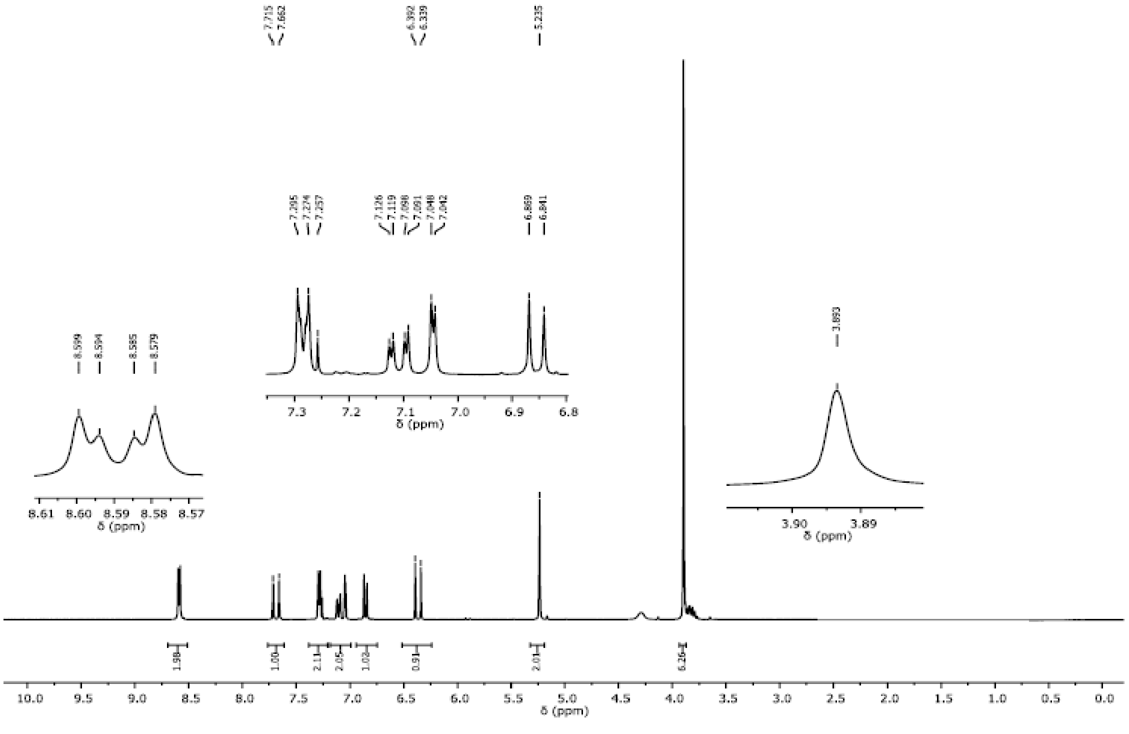

Figure S60 – ^{1}H NMR spectrum (300 MHz, $CDCl_3$) of **5l**.

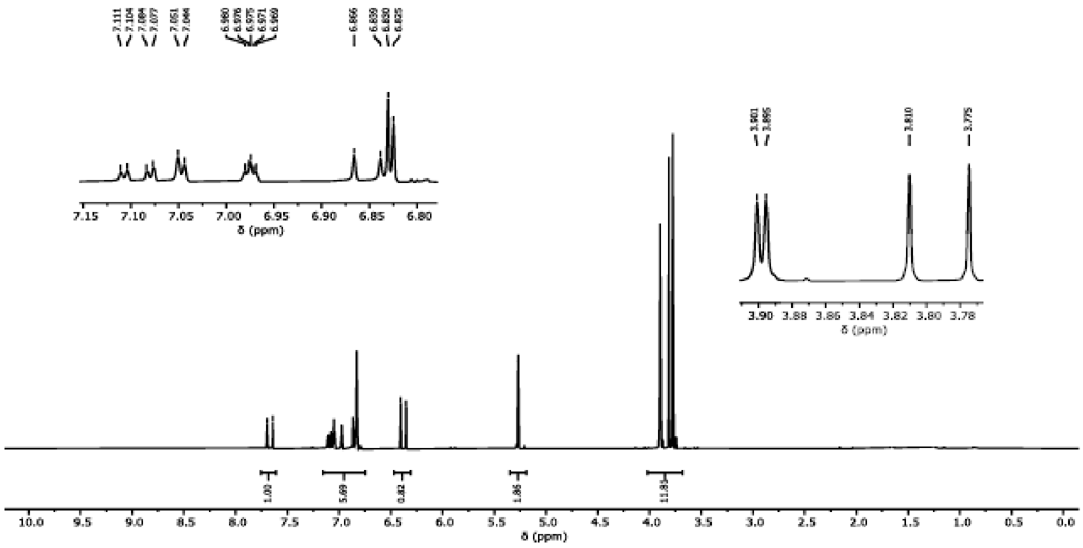

Figure S61 – ^{1}H NMR spectrum (300 MHz, $CDCl_3$) of **5m**.

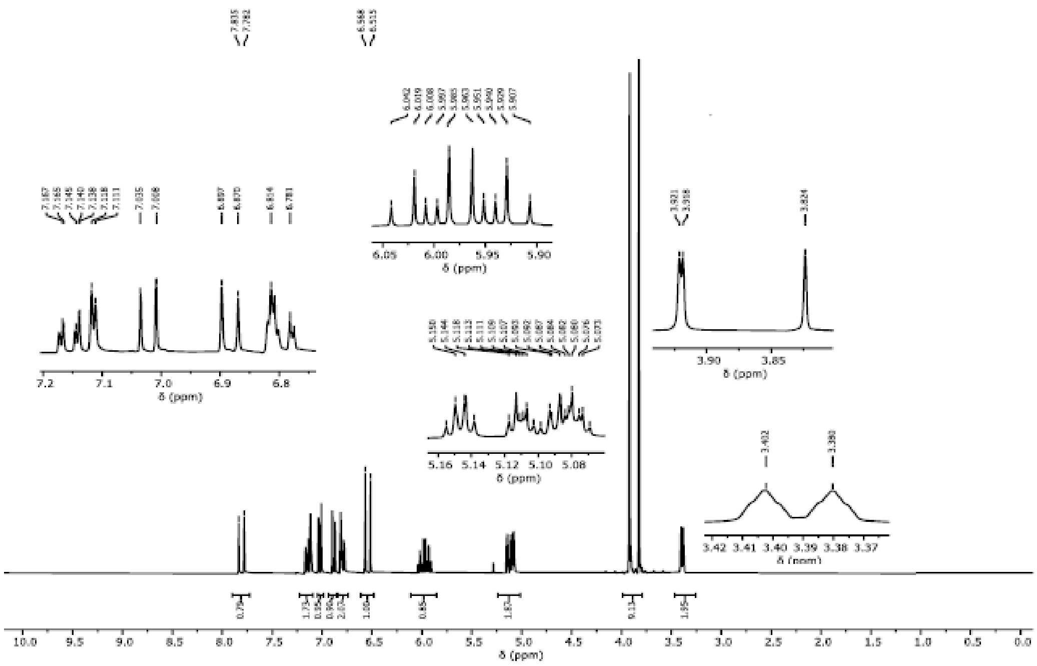

Figure S62 – ^{1}H NMR spectrum (300 MHz, $CDCl_3$) of **5n**.

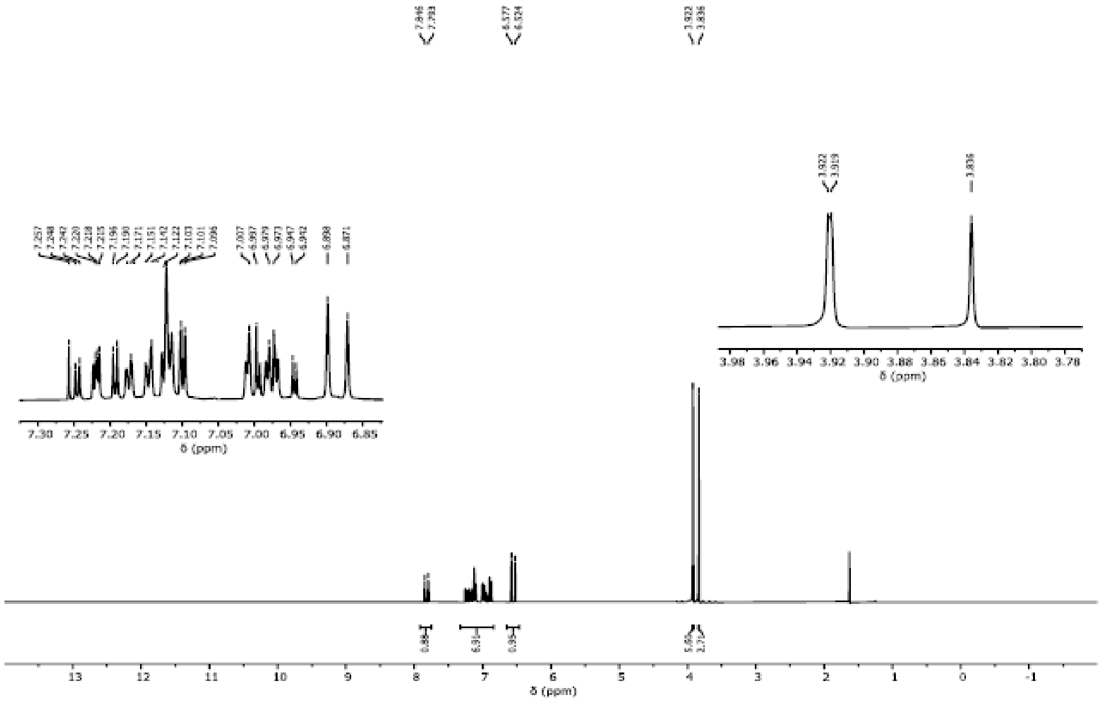

Figure S63 – ^{1}H NMR spectrum (300 MHz, $CDCl_3$) of **5o**.

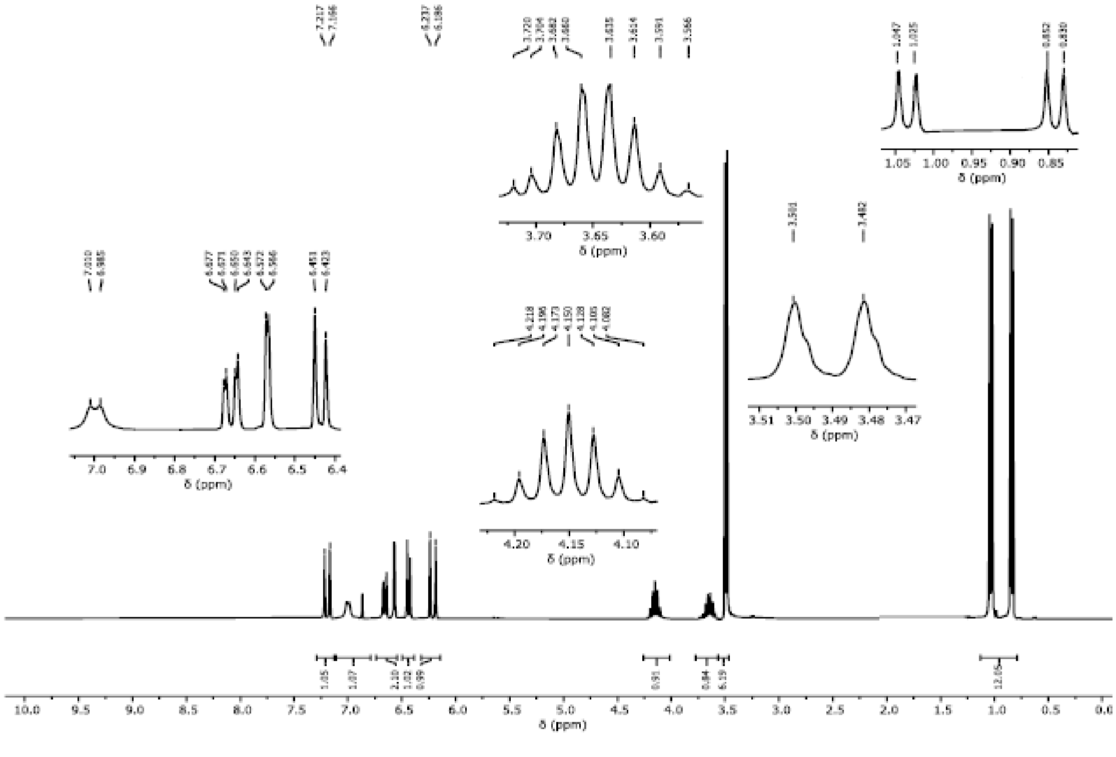

Figure S64 – ^{1}H NMR spectrum (300 MHz, $CDCl_3$) of **5p**.

^{13}C NUCLEAR MAGNETIC RESONANCE (NMR) SPECTRA OF COMPOUNDS 4a–4p AND 5a–5p

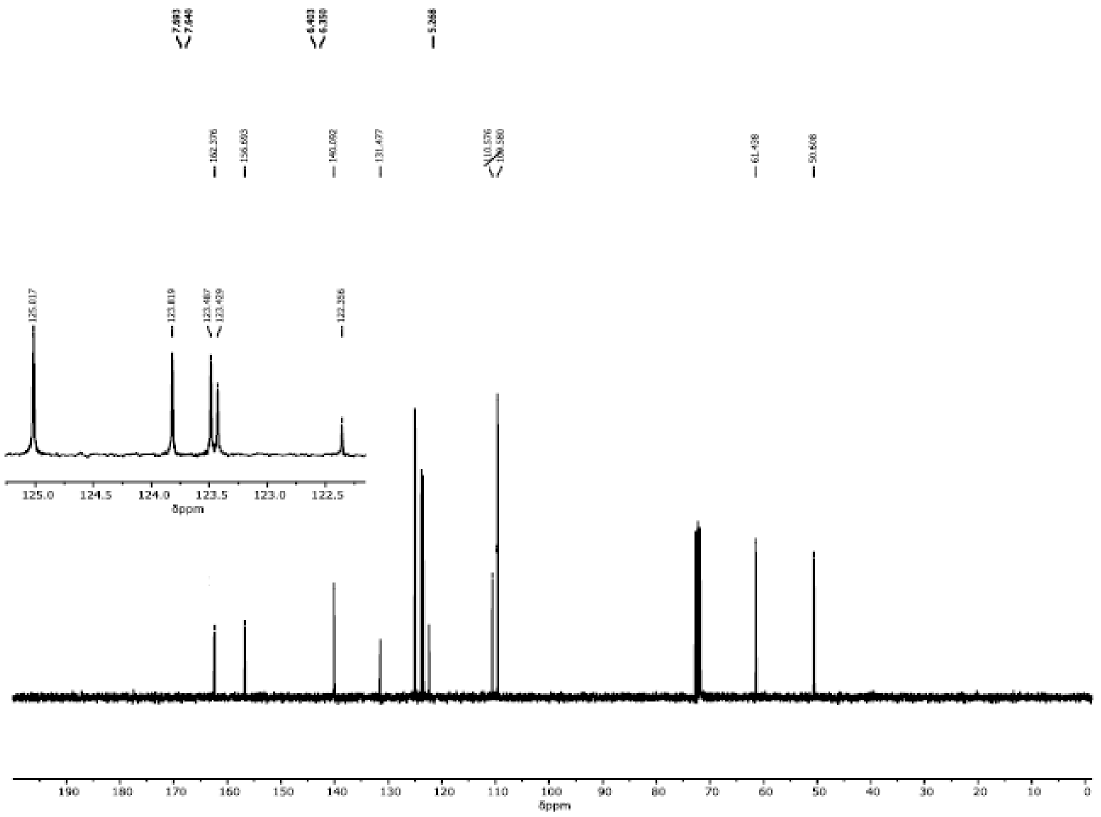

Figure S65 – ^{13}C NMR spectrum (75 MHz, $CDCl_3$) of **4a**.

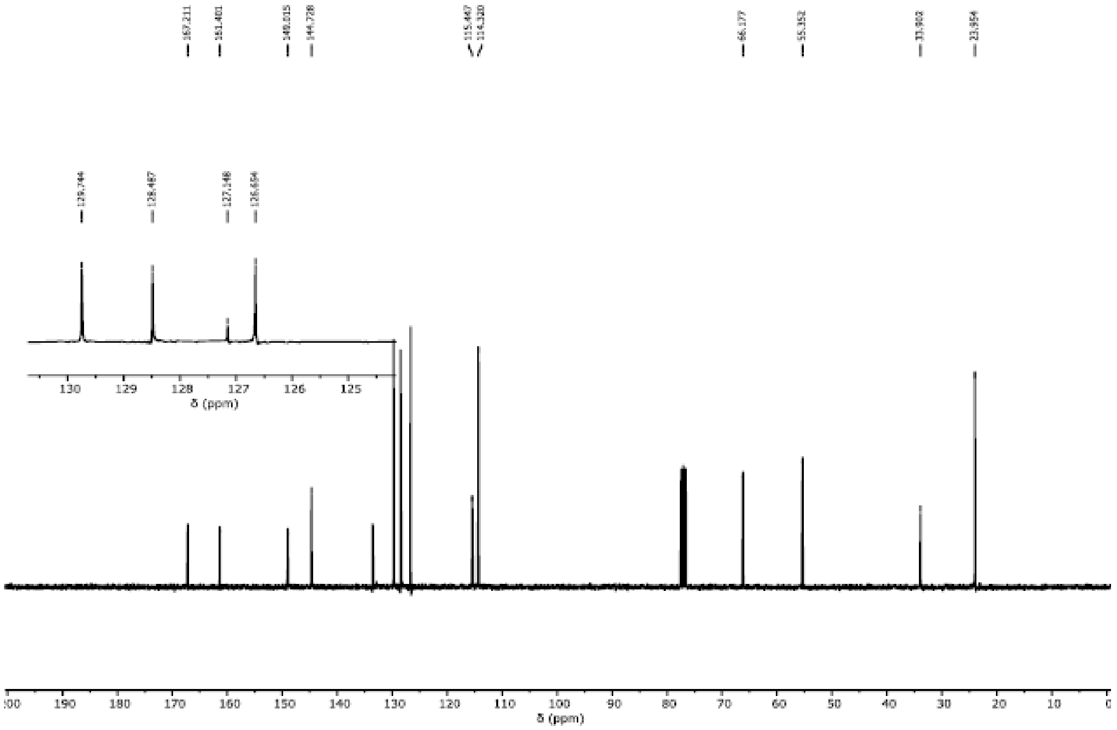

Figure S66 – ^{13}C NMR spectrum (75 MHz, $CDCl_3$) of **4b**.

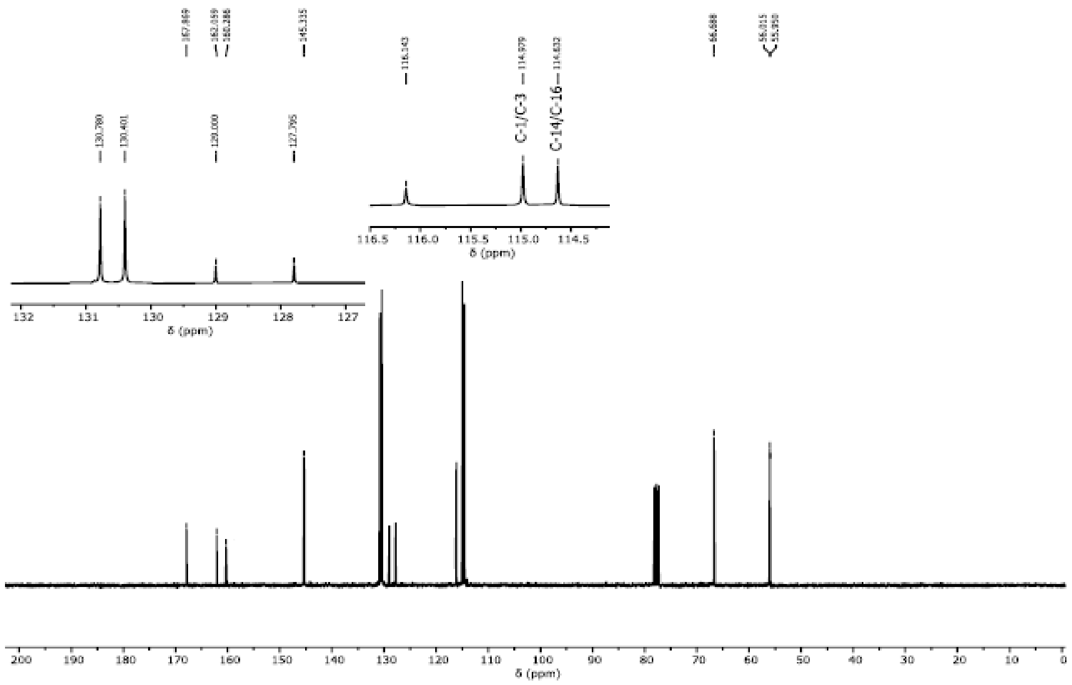

Figure S67 – ^{13}C NMR spectrum (75 MHz, $CDCl_3$) of **4c**.

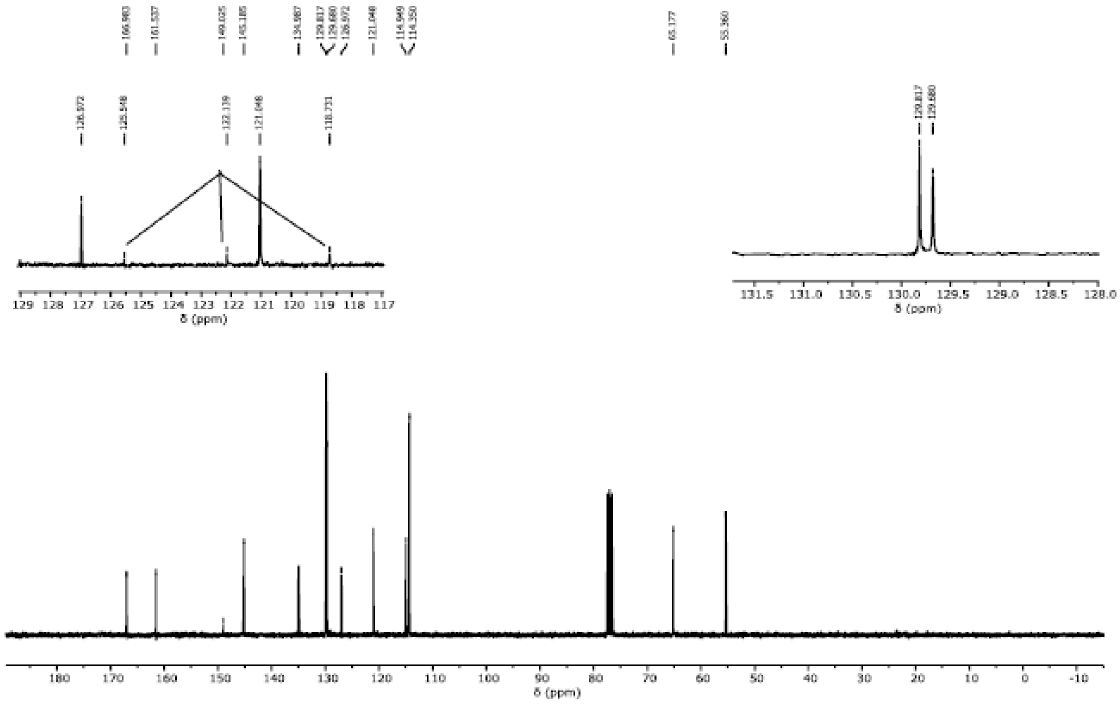

Figure S68 – ^{13}C NMR spectrum (75 MHz, $CDCl_3$) of **4d**.

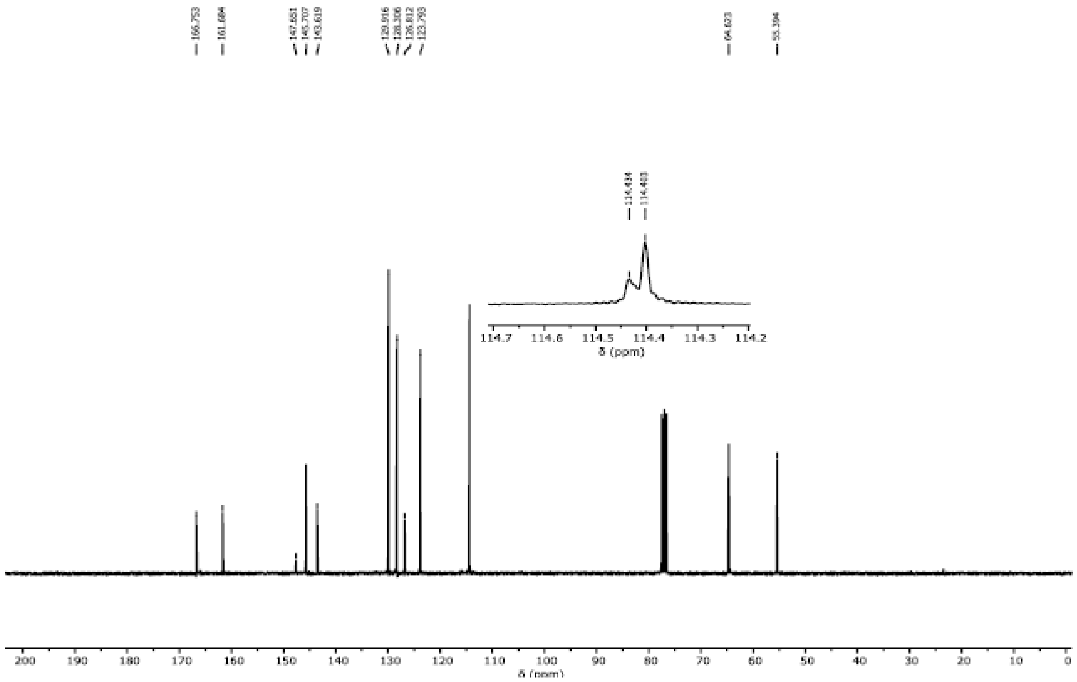

Figure S69 – ^{13}C NMR spectrum (75 MHz, $CDCl_3$) of **4e**.

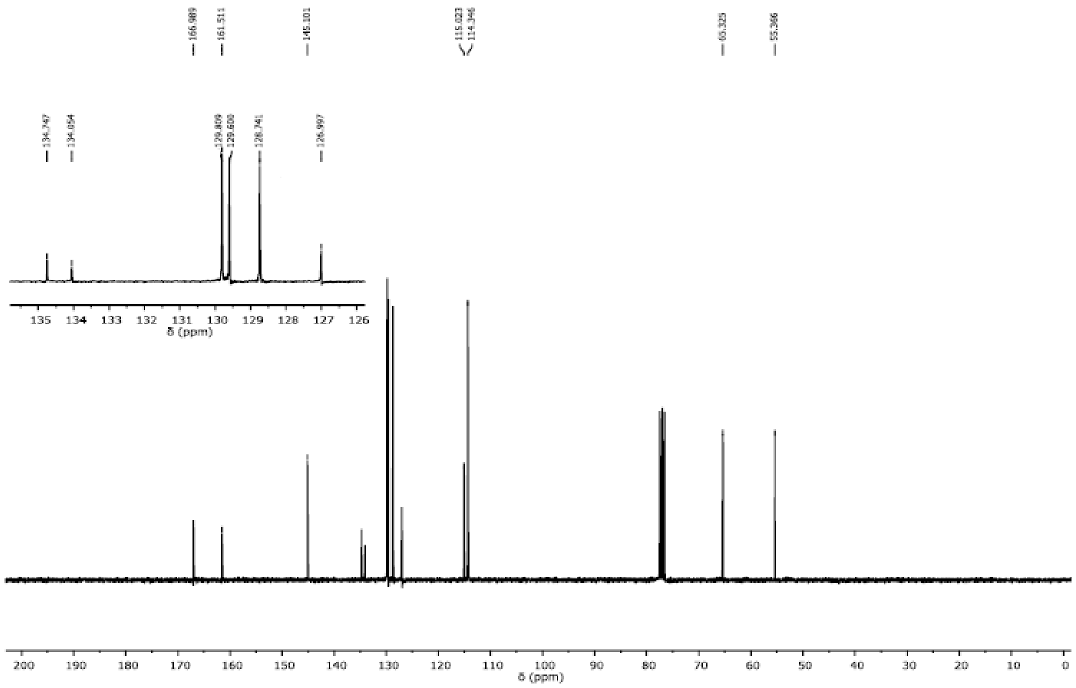

Figure S70 – ^{13}C NMR spectrum (75 MHz, $CDCl_3$) of **4f**.

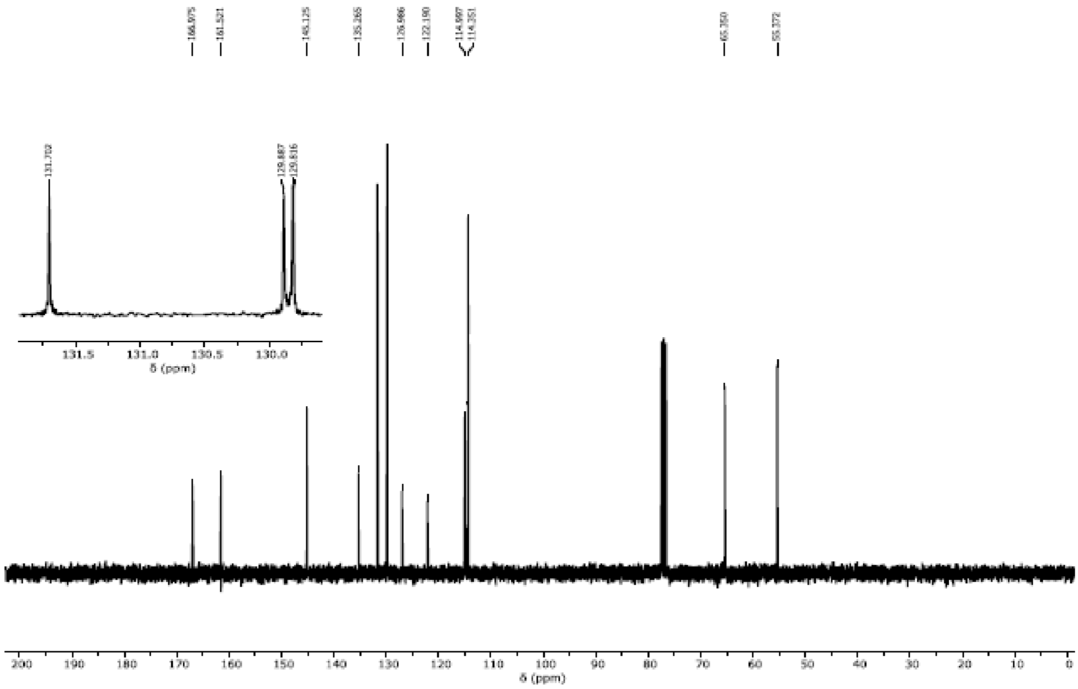

Figure S71 – ^{13}C NMR spectrum (75 MHz, $CDCl_3$) of **4g**.

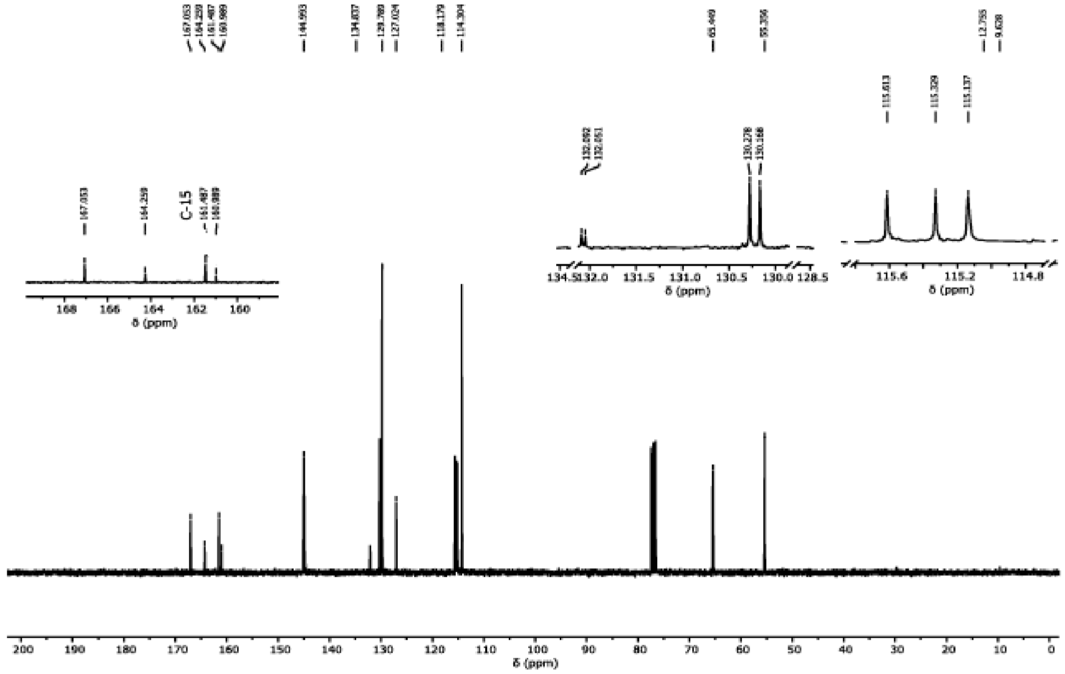

Figure S72 – ^{13}C NMR spectrum (75 MHz, $CDCl_3$) of **4h**.

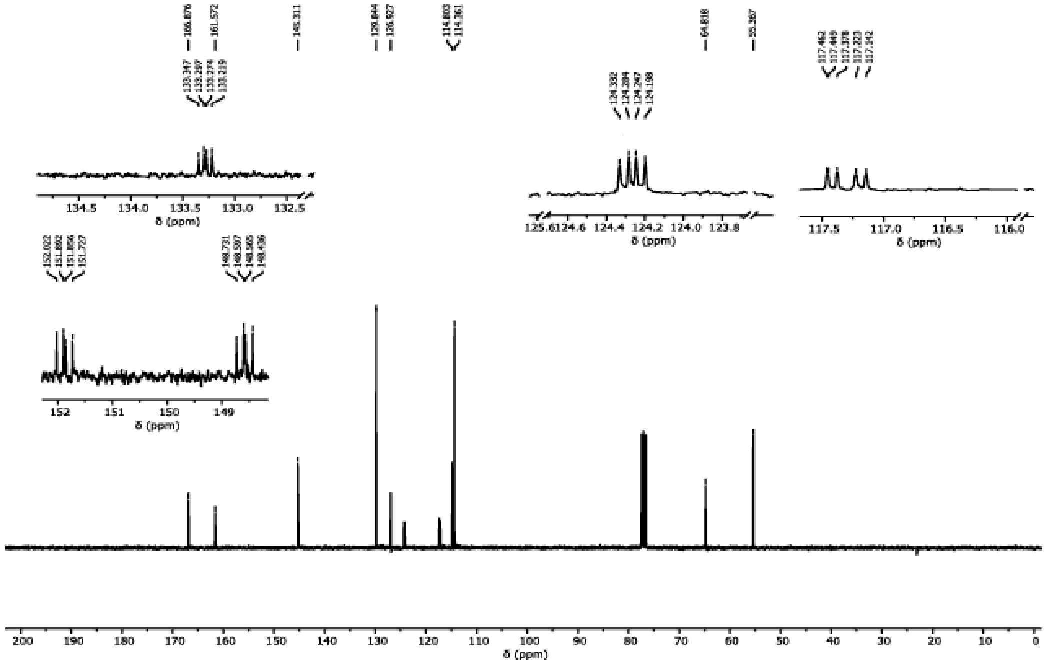

Figure S73 – ^{13}C NMR spectrum (75 MHz, $CDCl_3$) of **4i**.

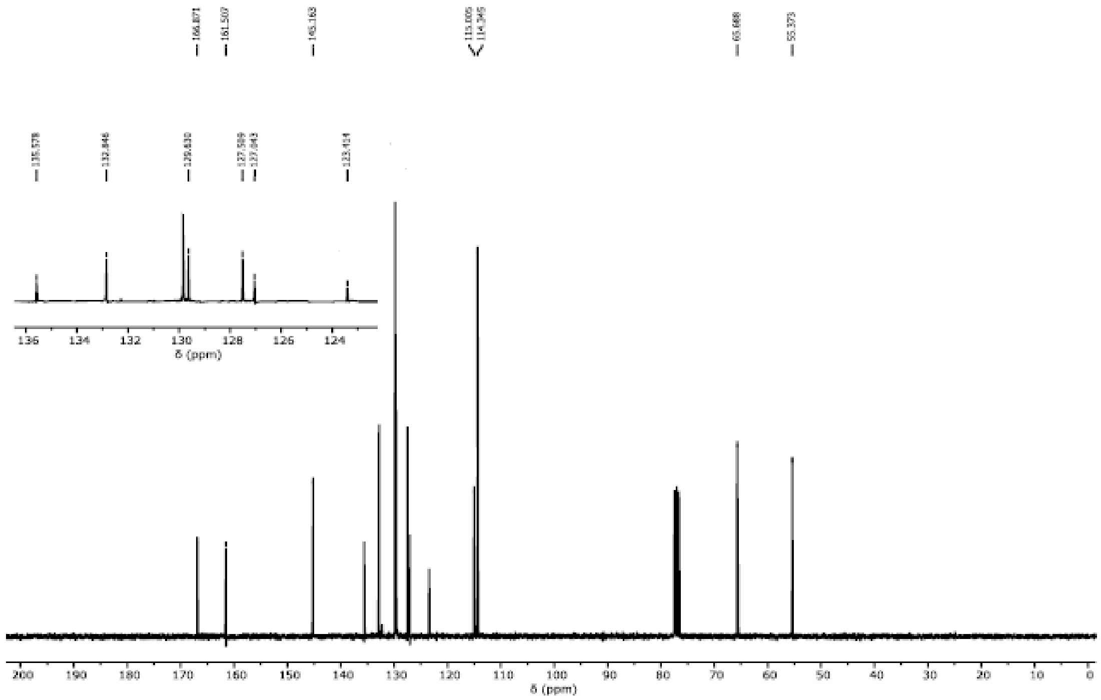

Figure S74 – ^{13}C NMR spectrum (75 MHz, $CDCl_3$) of **4j**.

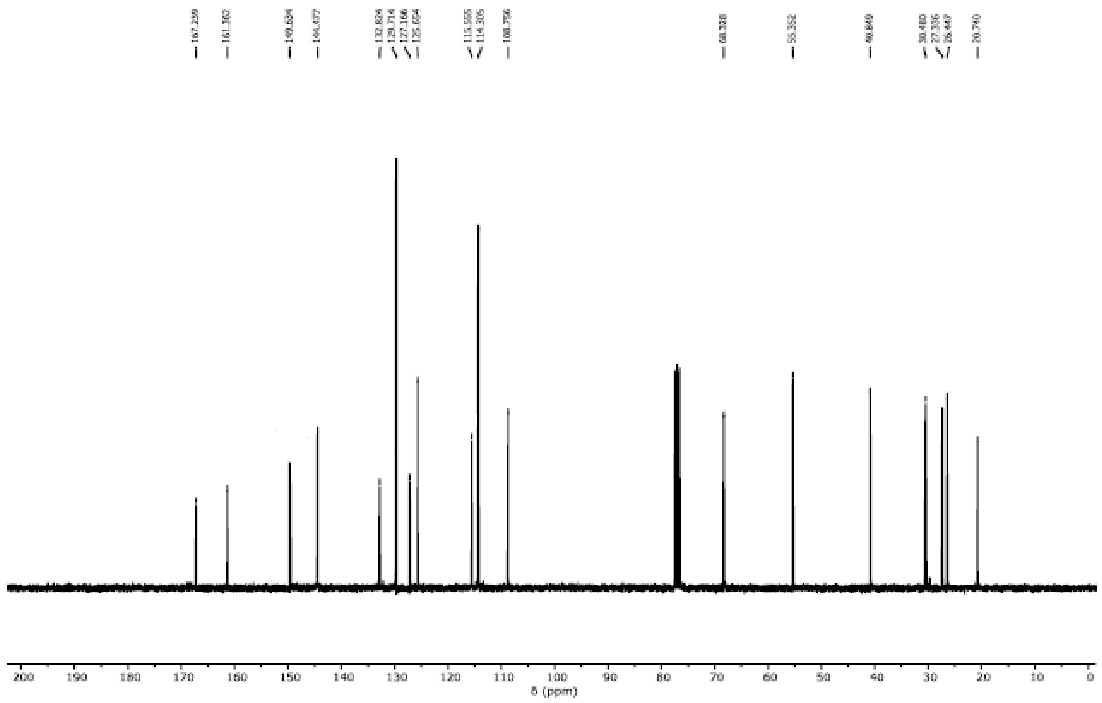

Figure S75 – ^{13}C NMR spectrum (75 MHz, $CDCl_3$) of **4k**.

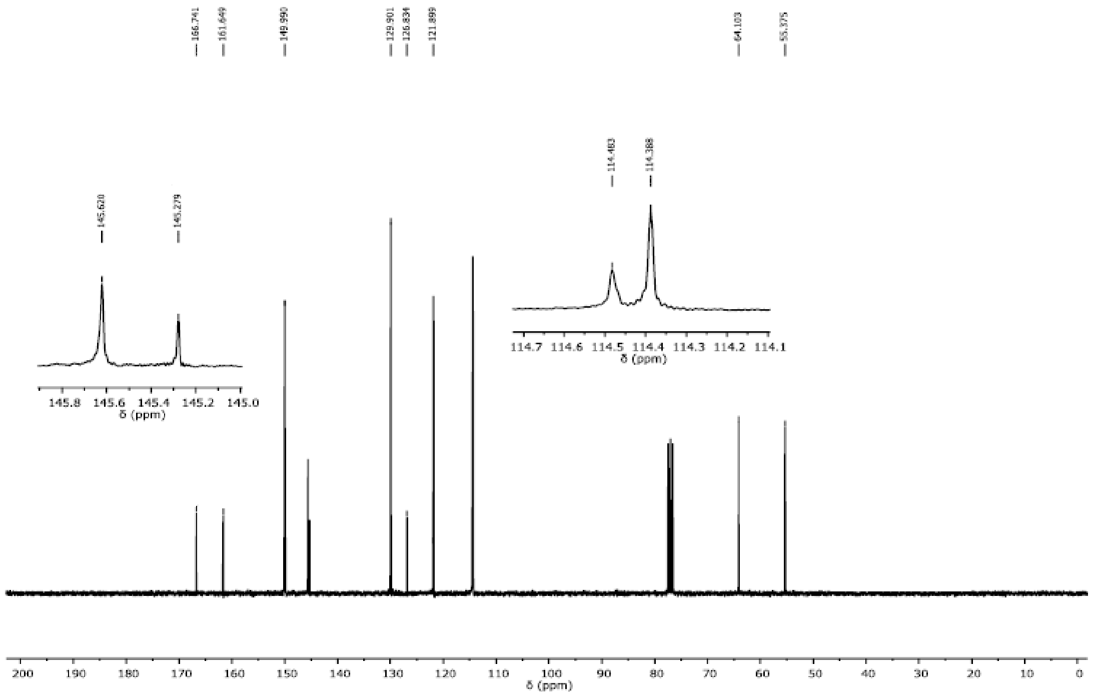

Figure S76 – ^{13}C NMR spectrum (75 MHz, $CDCl_3$) of **4l**.

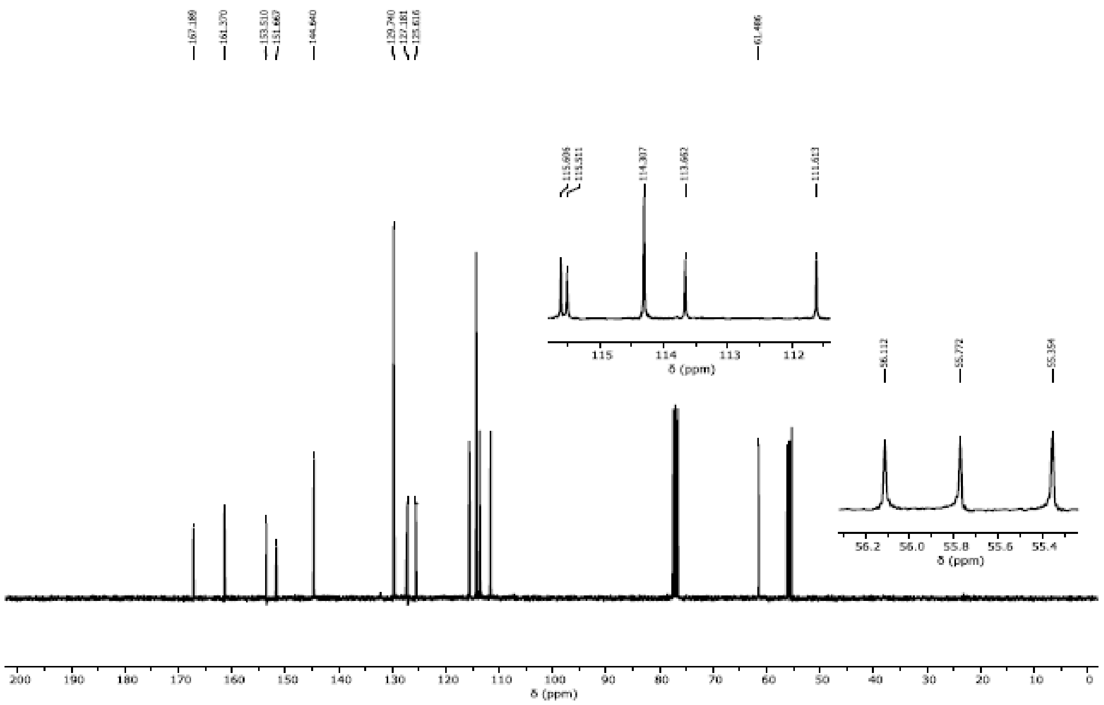

Figure S77 – ^{13}C NMR spectrum (75 MHz, $CDCl_3$) of **4m**.

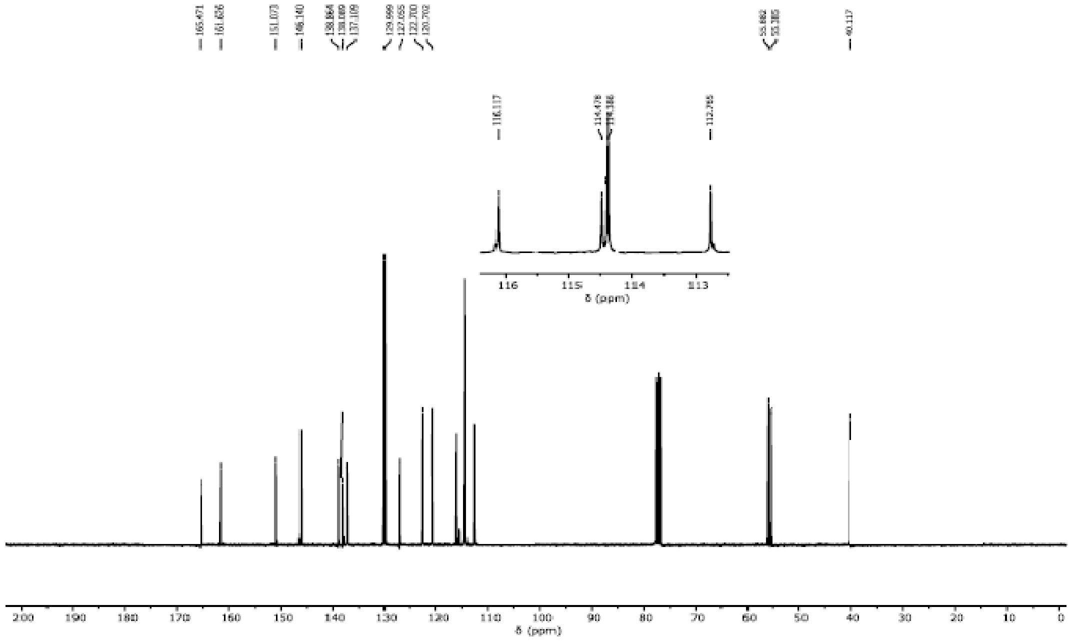

Figure S78 – ^{13}C NMR spectrum (75 MHz, $CDCl_3$) of **4n**.

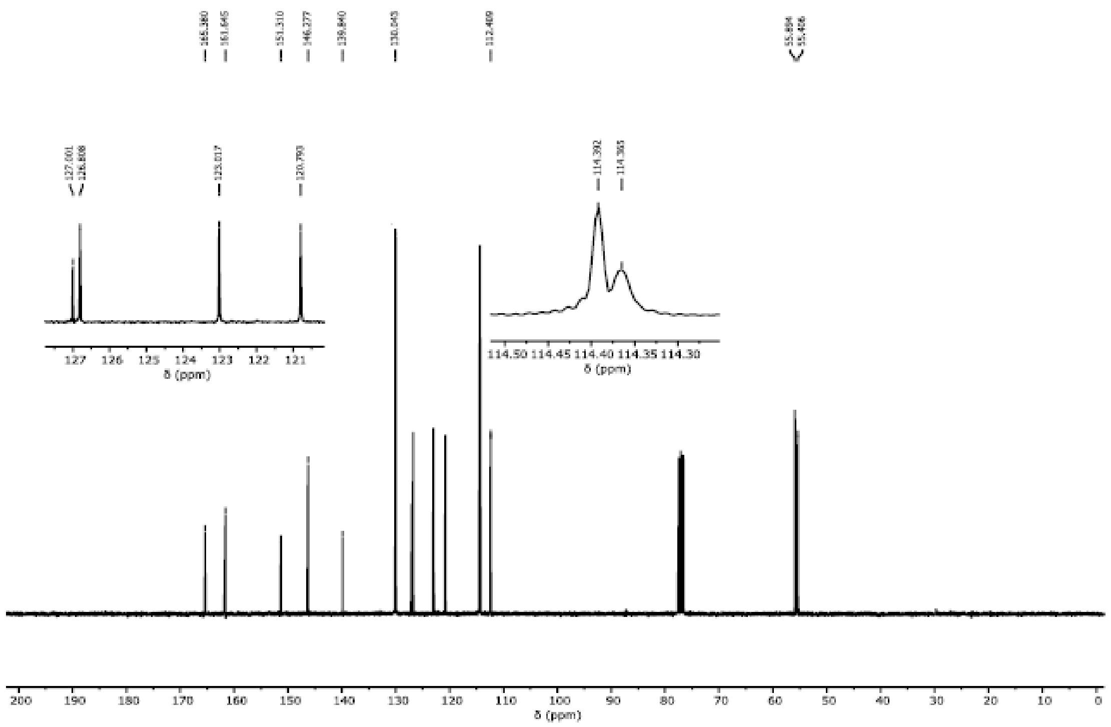

Figure S79 – ^{13}C NMR spectrum (75 MHz, $CDCl_3$) of **4o**.

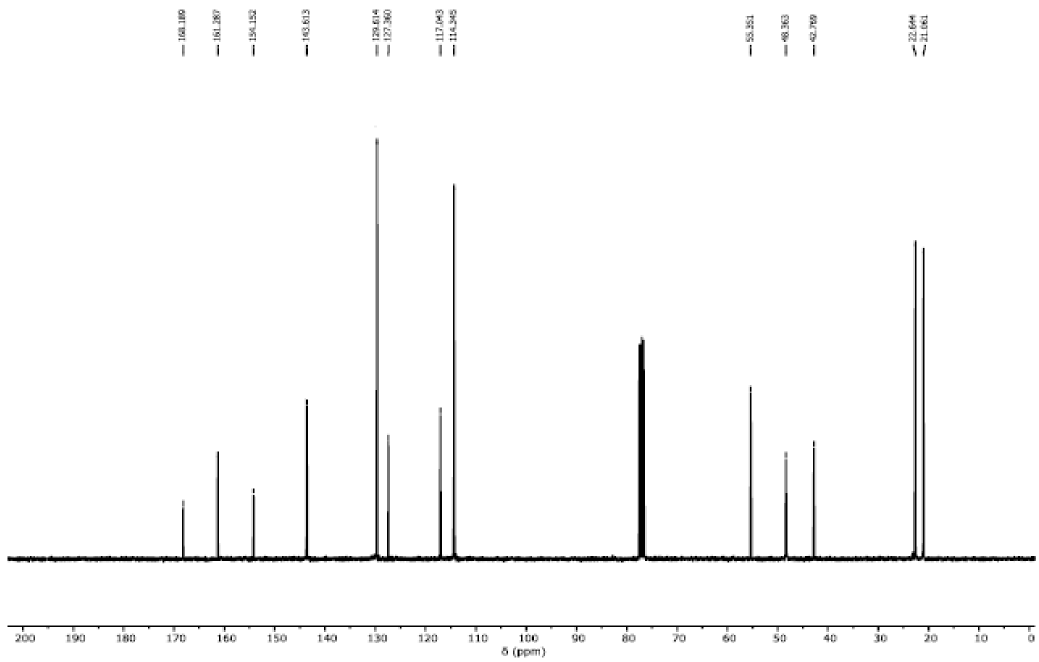

Figure S70 – ^{13}C NMR spectrum (75 MHz, $CDCl_3$) of **4p**.

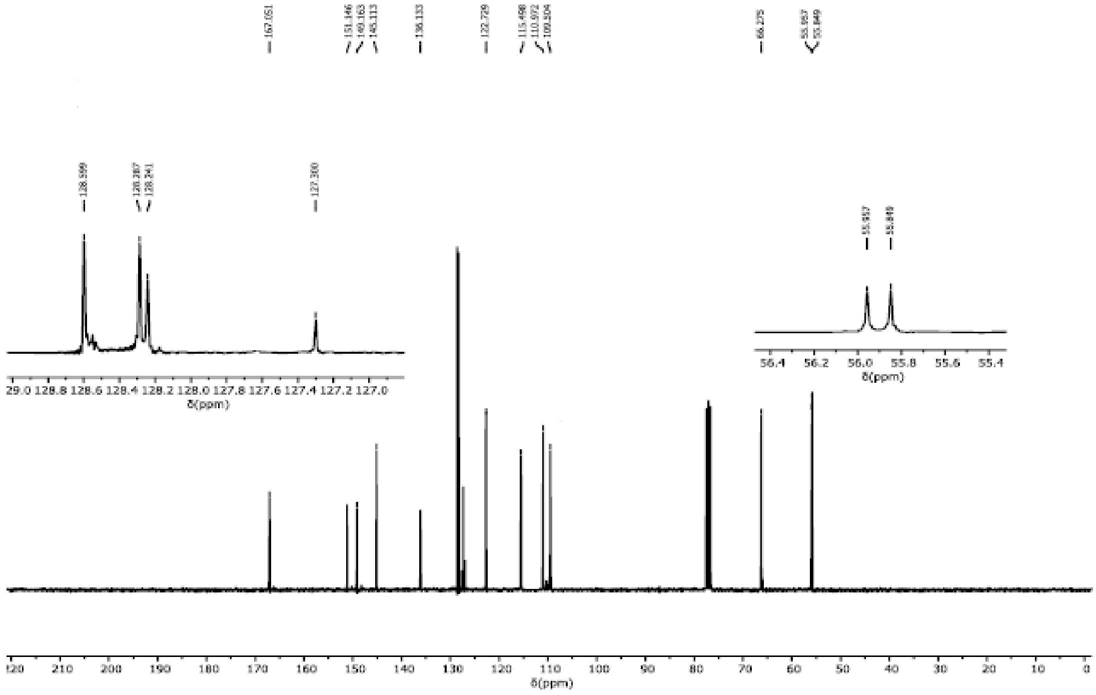

Figure S71 – ^{13}C NMR spectrum (75 MHz, $CDCl_3$) of **5a**.

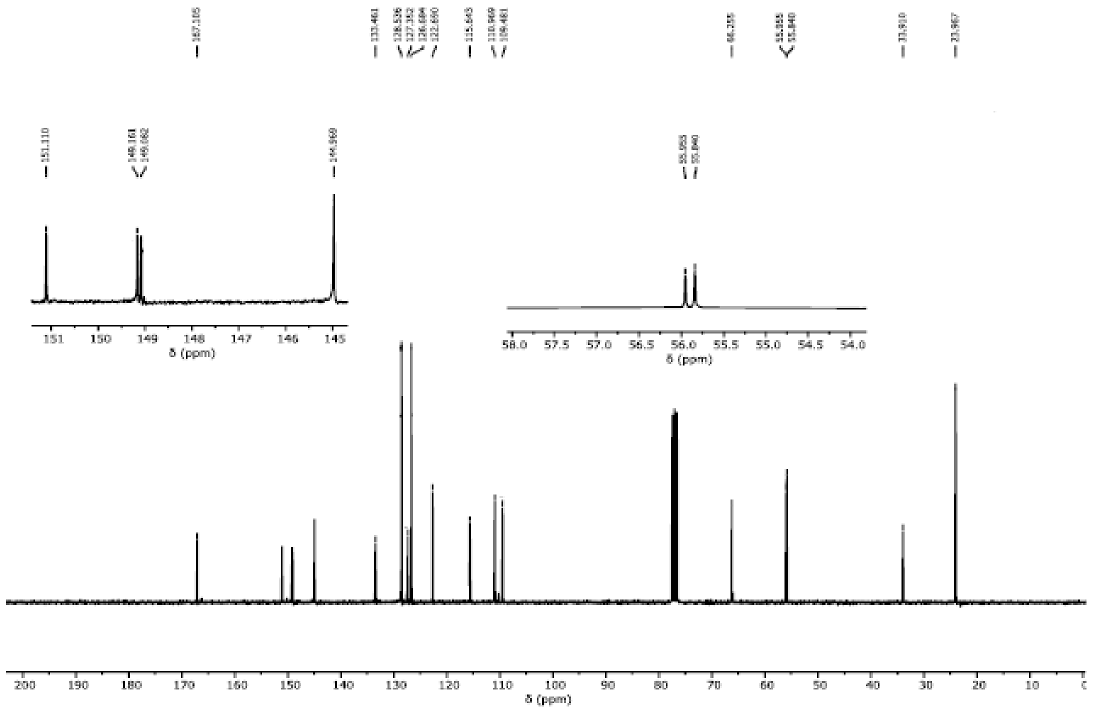

Figure S72 – ^{13}C NMR spectrum (75 MHz, $CDCl_3$) of **5b**.

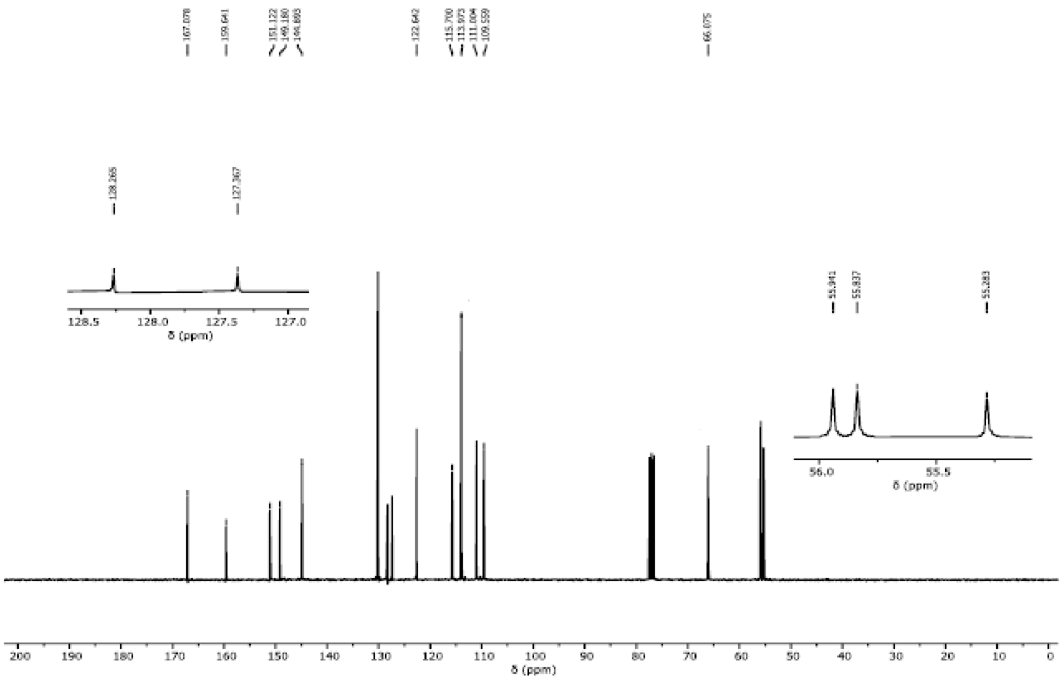

Figure S73 – ^{13}C NMR spectrum (75 MHz, $CDCl_3$) of **5c**.

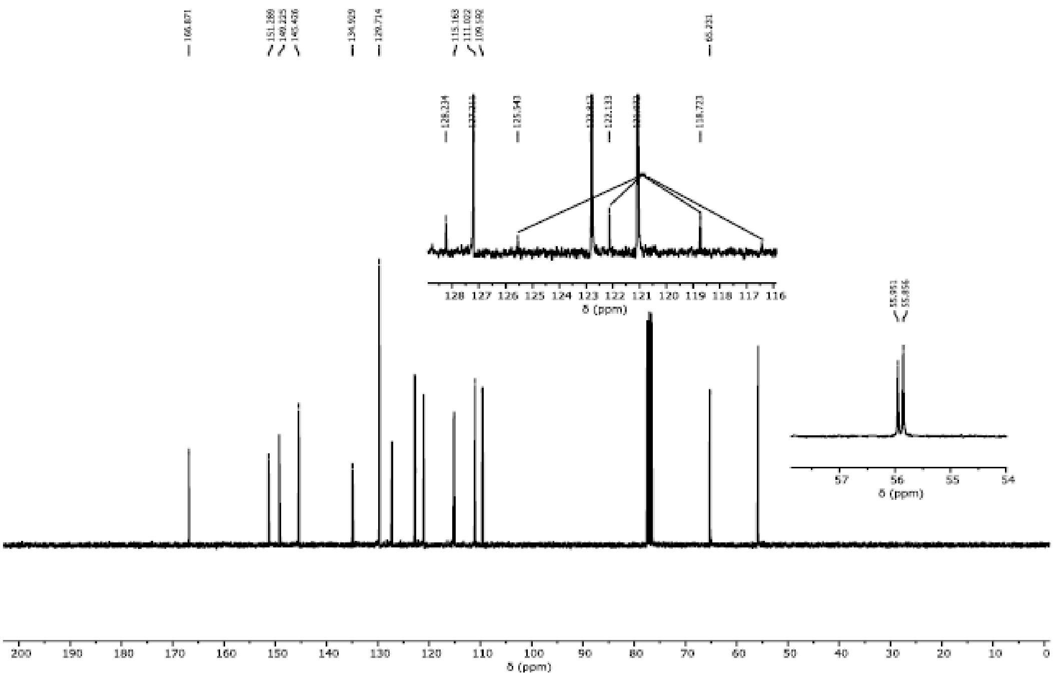

Figure S74 – ^{13}C NMR spectrum (75 MHz, $CDCl_3$) of **5d**.

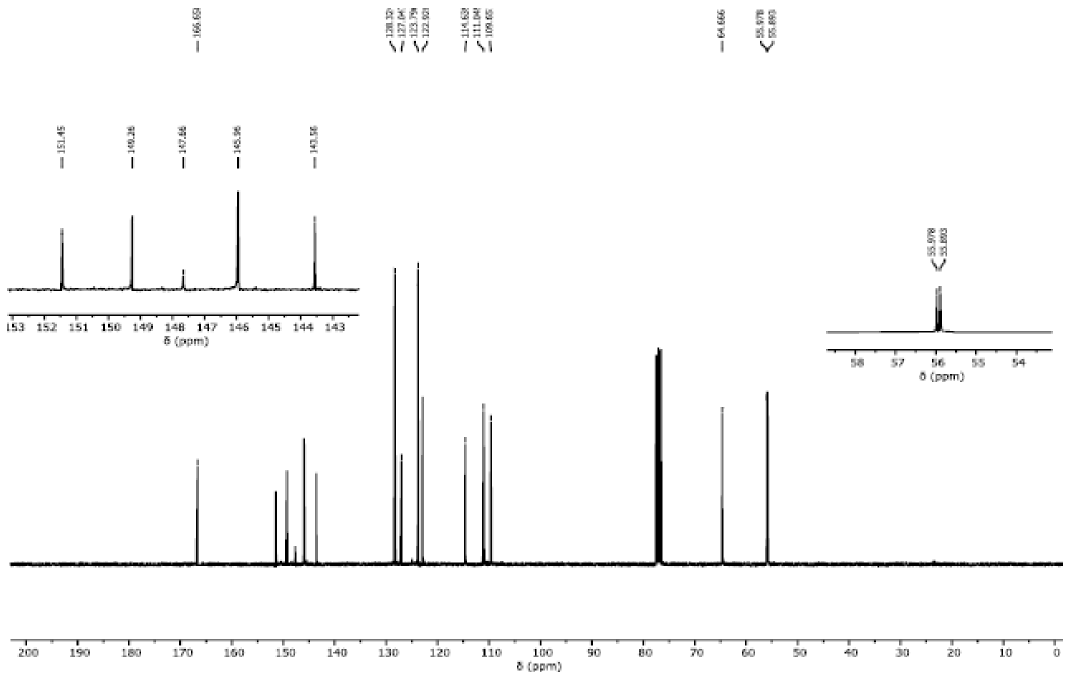

Figure S75 – ^{13}C NMR spectrum (75 MHz, $CDCl_3$) of **5e**.

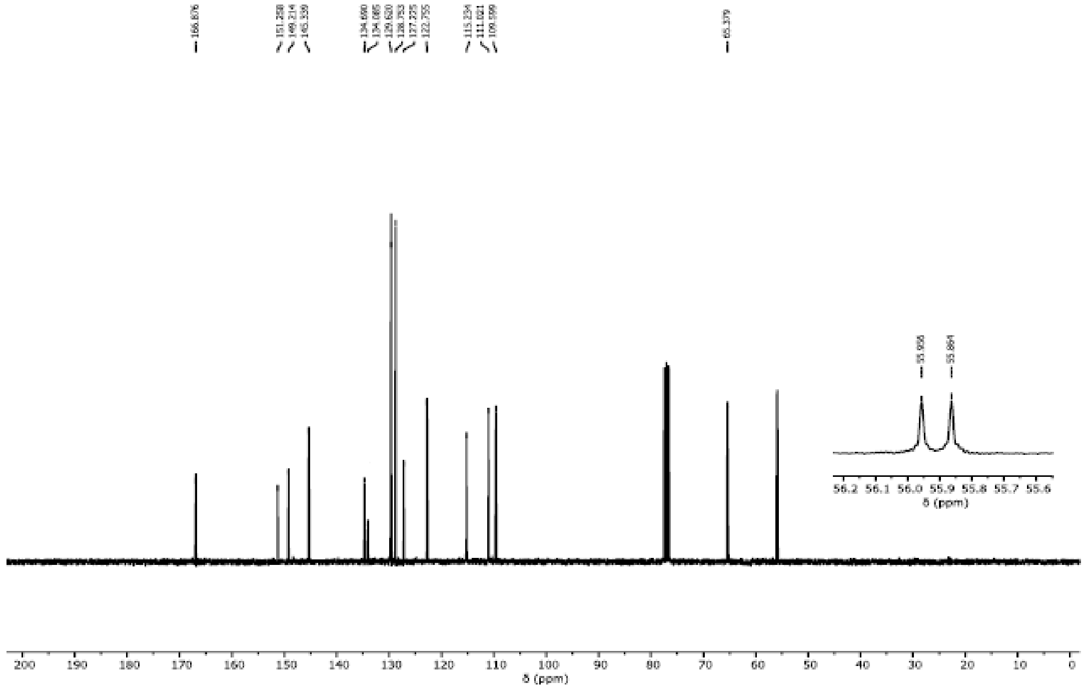

Figure S76 – ^{13}C NMR spectrum (75 MHz, $CDCl_3$) of **5f**.

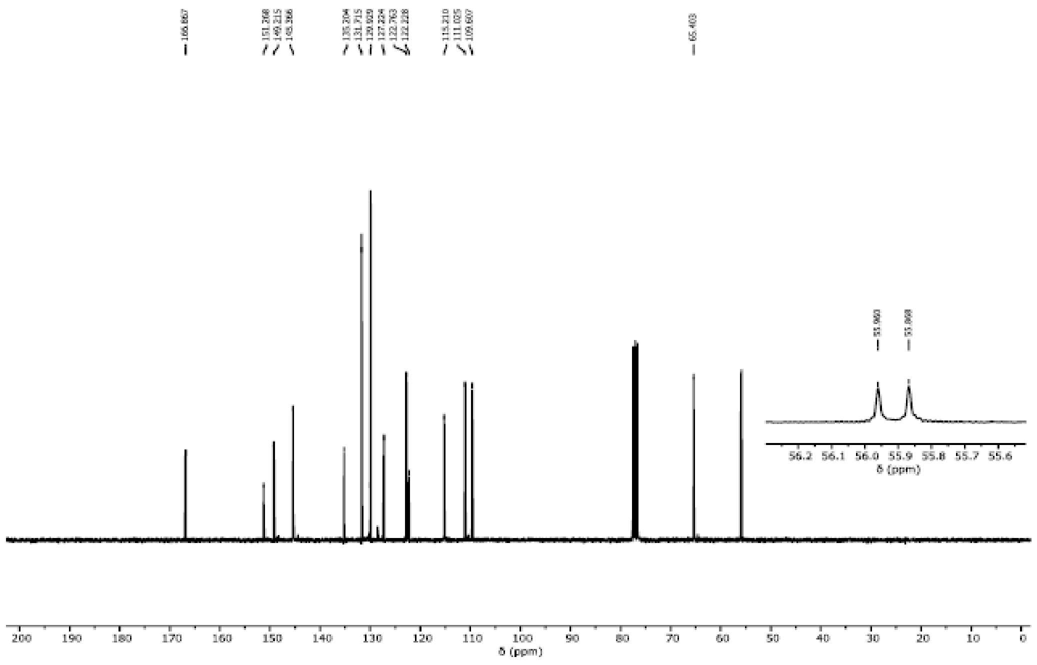

Figure S77 – ^{13}C NMR spectrum (75 MHz, CDCl$_3$) of **5g**.

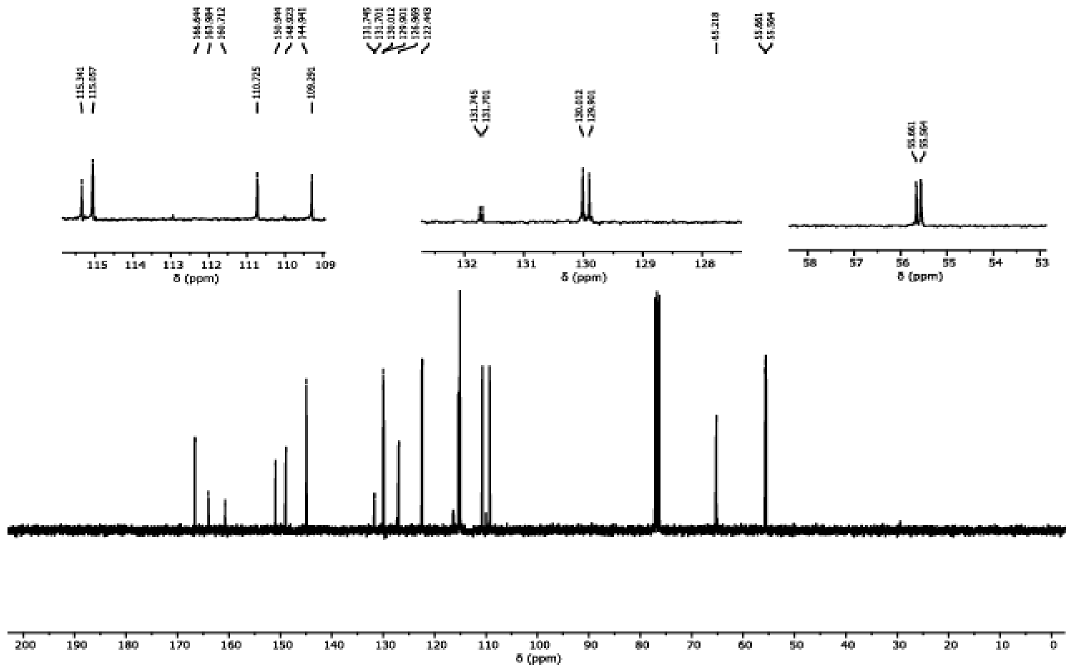

Figure S78 – ^{13}C NMR spectrum (75 MHz, CDCl$_3$) of **5h**.

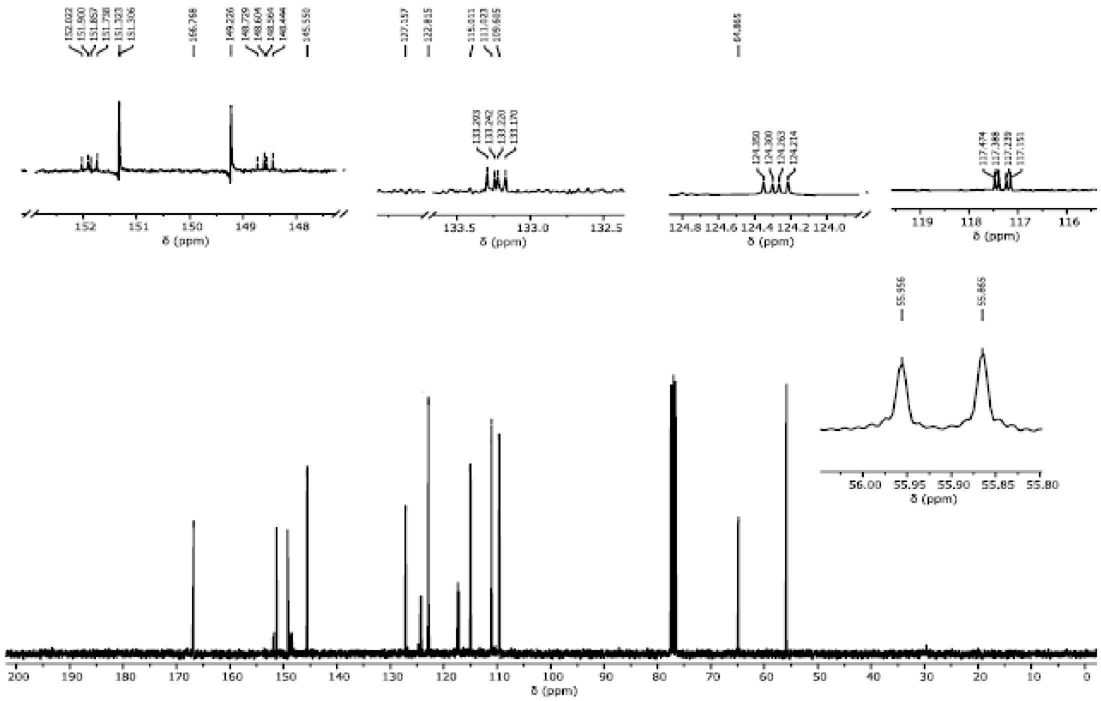

Figure S79 – ^{13}C NMR spectrum (75 MHz, $CDCl_3$) of **5i**.

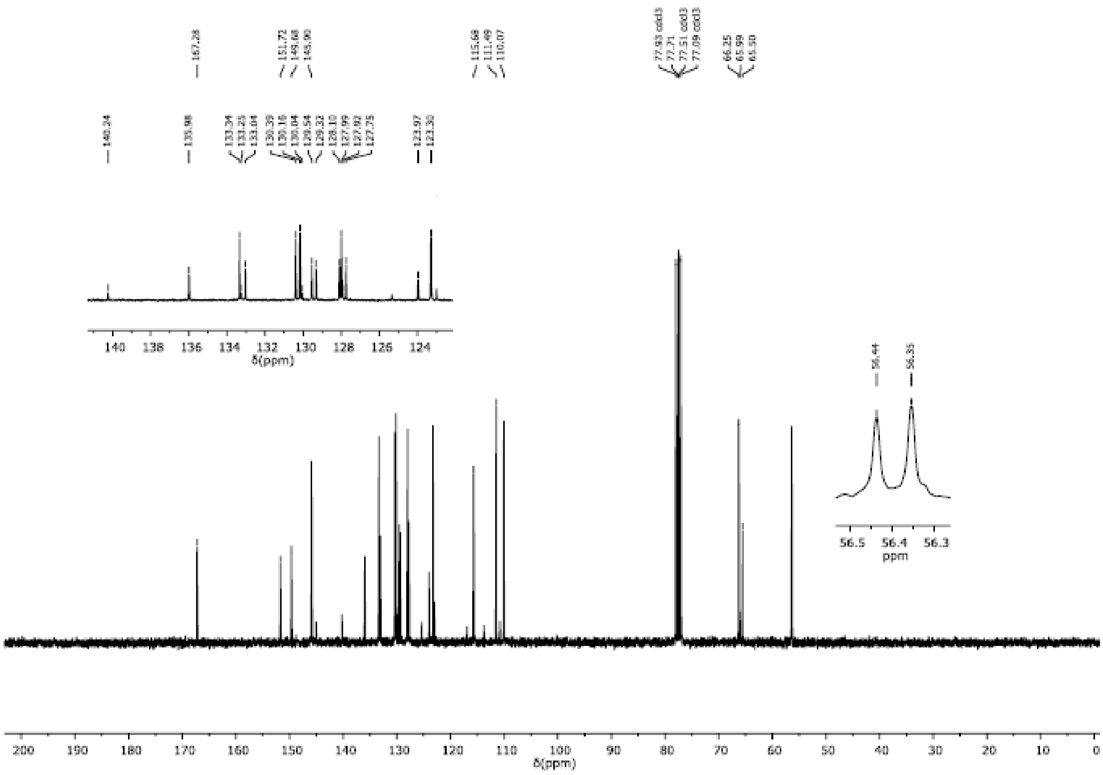

Figure S80 – ^{13}C NMR spectrum (75 MHz, $CDCl_3$) of **5j**.

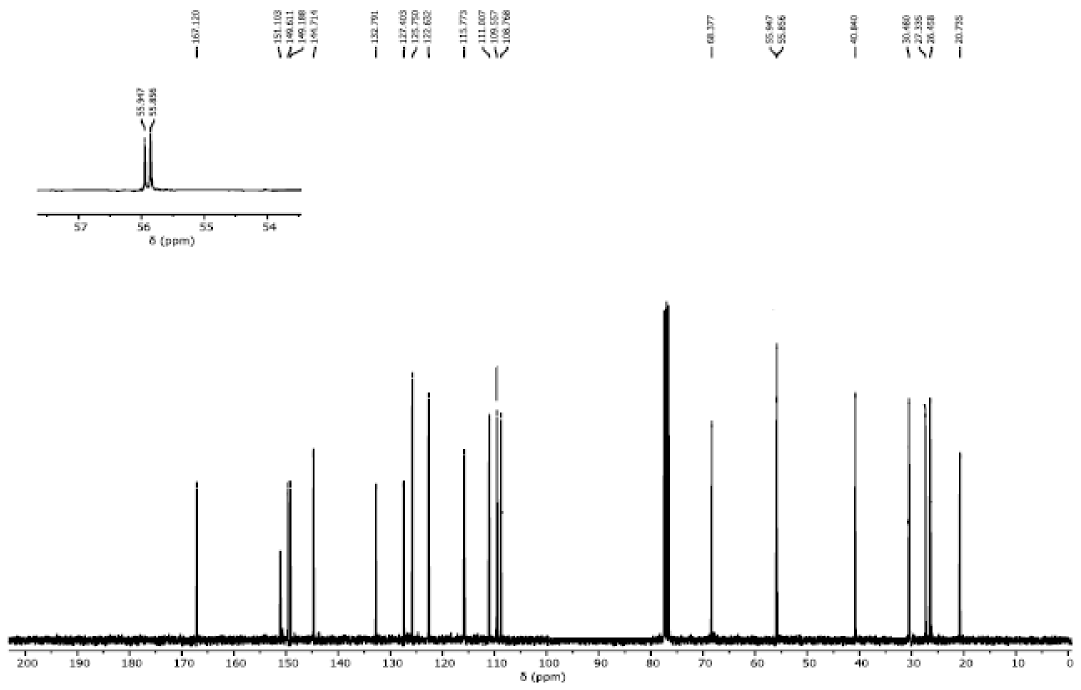

Figure S81 – ^{13}C NMR spectrum (75 MHz, $CDCl_3$) of **5k**.

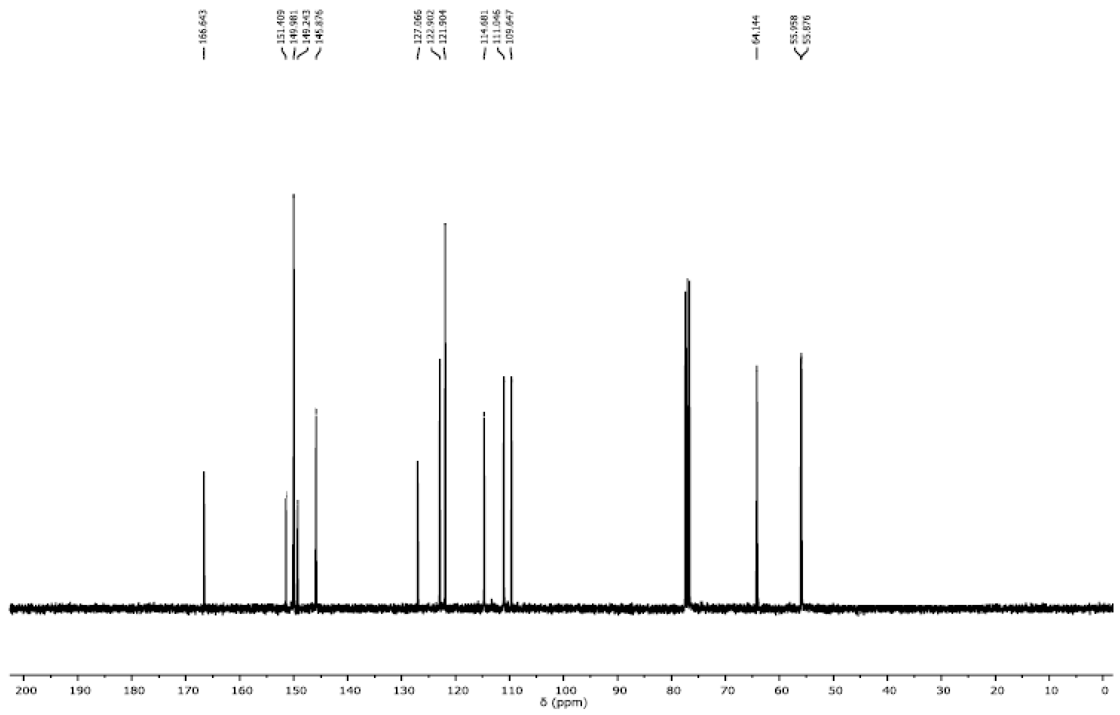

Figure S82 – ^{13}C NMR spectrum (75 MHz, $CDCl_3$) of **5l**.

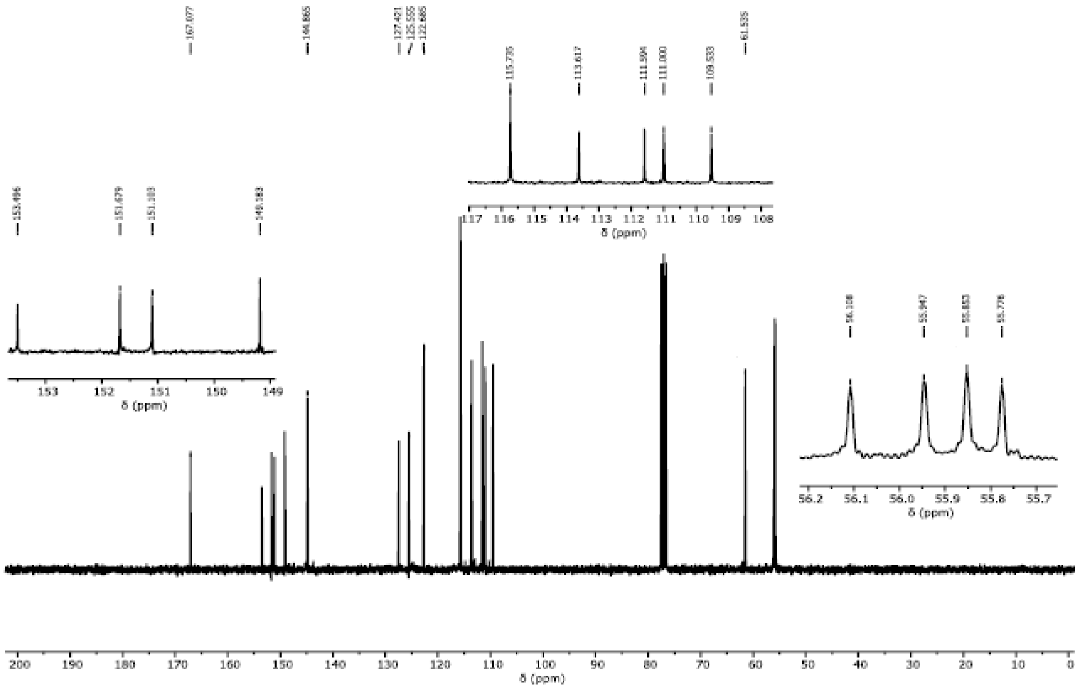

Figure S83 – ^{13}C NMR spectrum (75 MHz, $CDCl_3$) of **5m**.

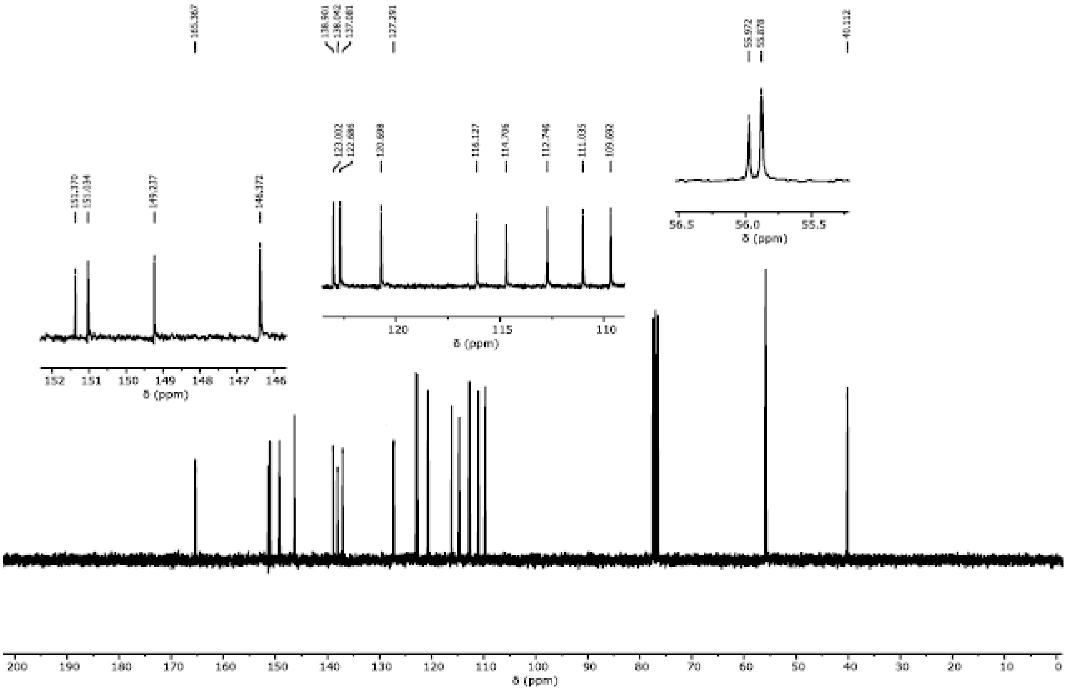

Figure S84 – ^{13}C NMR spectrum (75 MHz, $CDCl_3$) of **5n**.

.

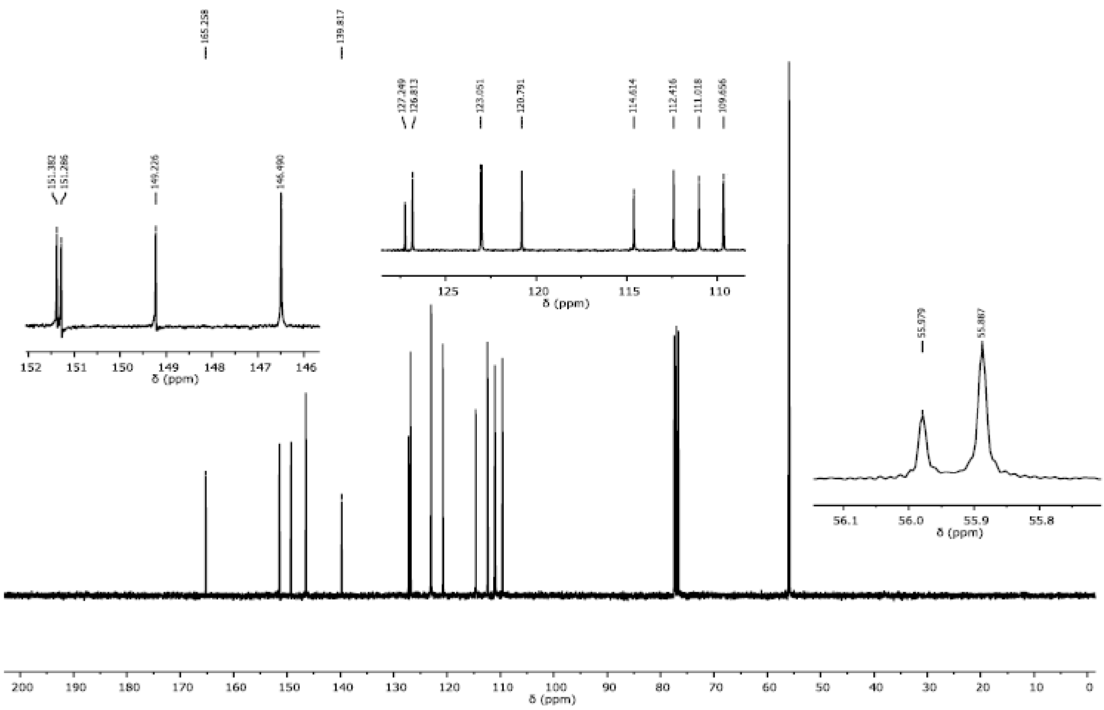

Figure S85 – ^{13}C NMR spectrum (75 MHz, $CDCl_3$) of **5o**.

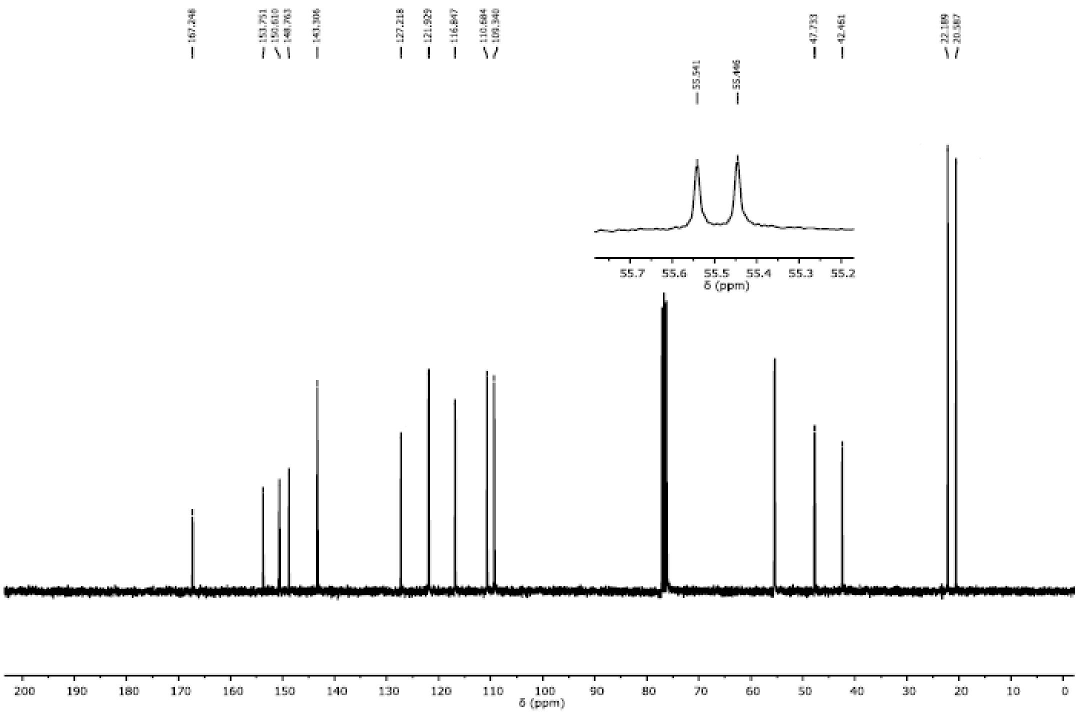

Figure S86 – ^{13}C NMR spectrum (75 MHz, $CDCl_3$) of **5p**.

MASS SPECTRA OF SELECTED COMPOUNDS

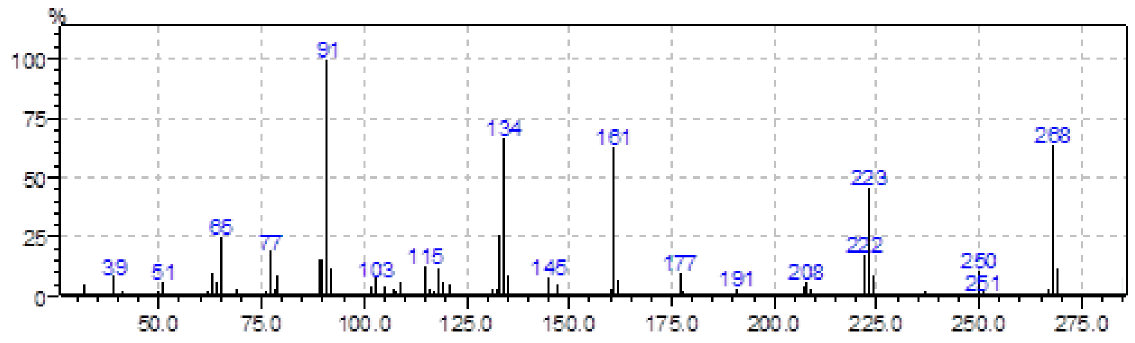

Figure S87 – Mass spectrum of compound **4a**.

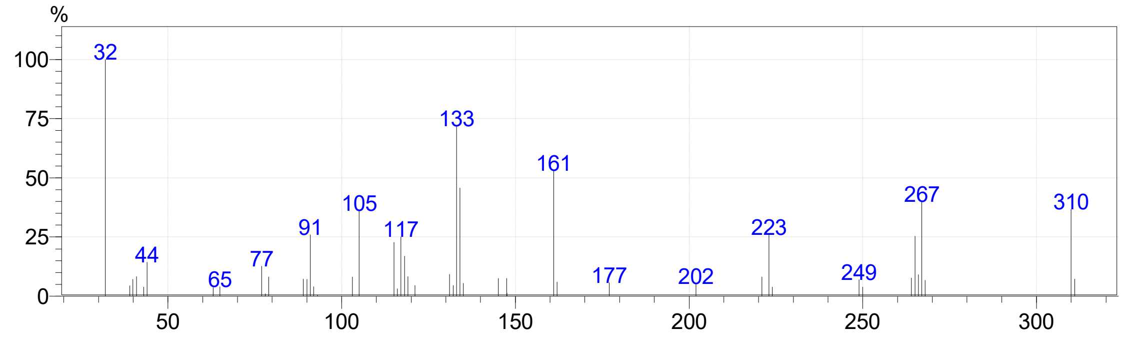

Figure S88 – Mass spectrum of compound **4b**.

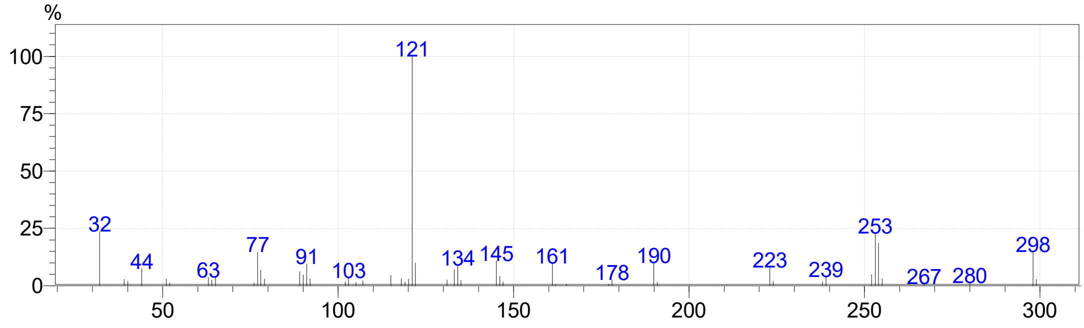

Figure S89 – Mass spectrum of compound **4c**.

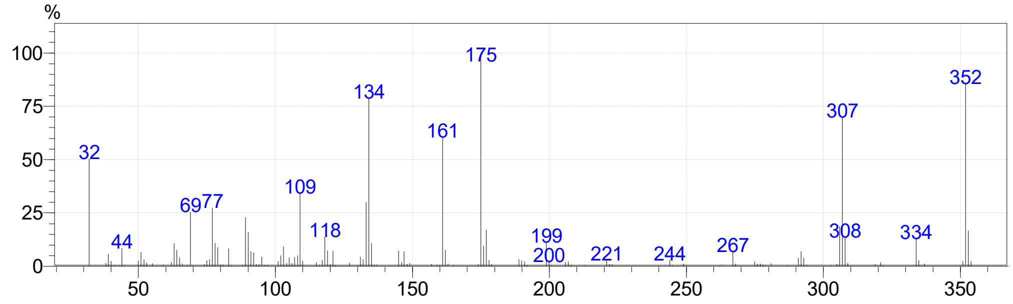

Figure S90 – Mass spectrum of compound **4d**.

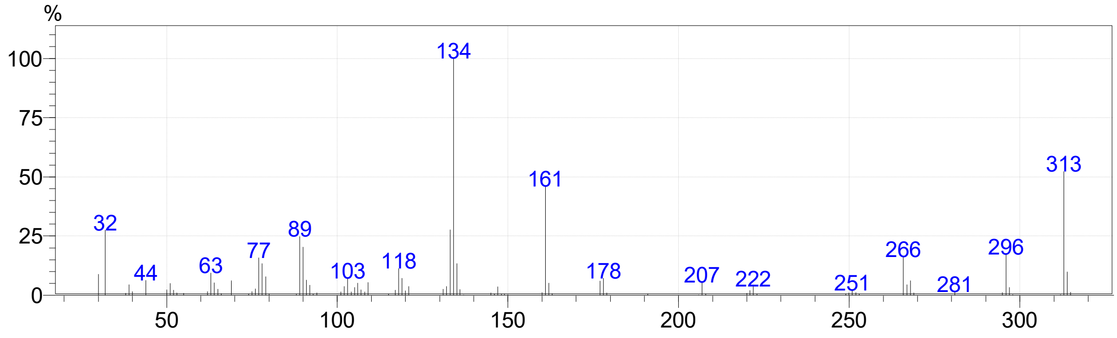

Figure S91 – Mass spectrum of compound **4e**.

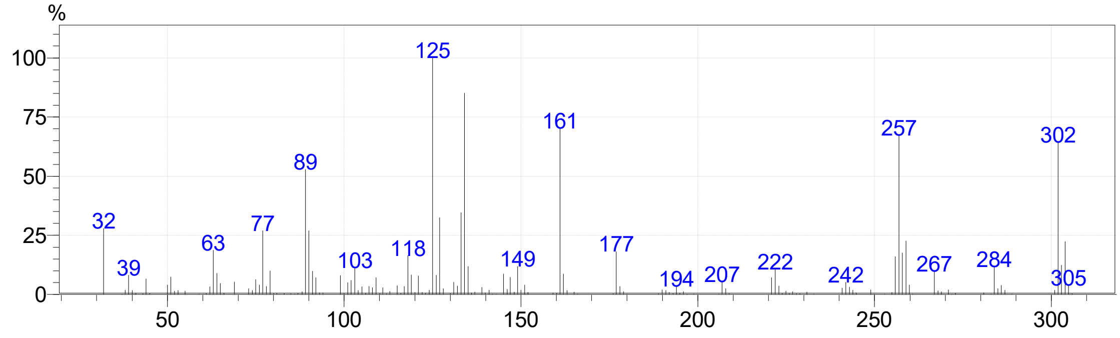

Figure 92 – Mass spectrum of compound **4f**.

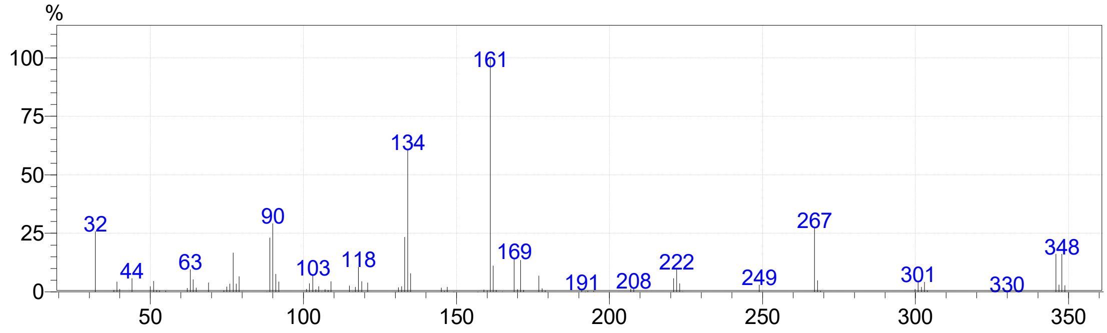

Figure 93 – Mass spectrum of compound **4g**.

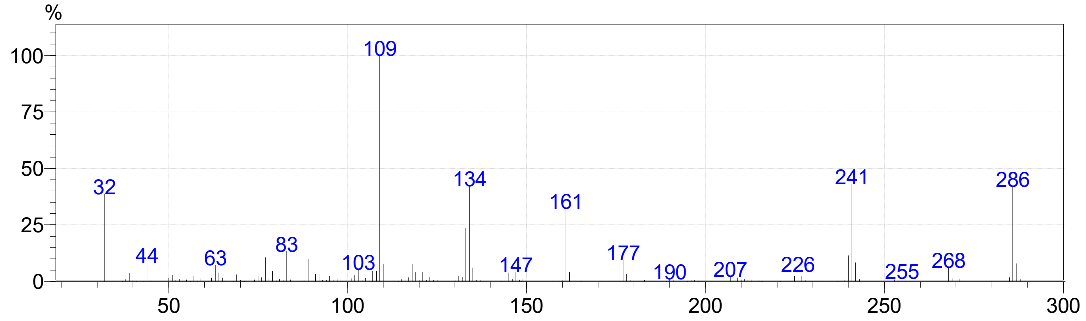

Figure 94 – Mass spectrum of compound **4h**.

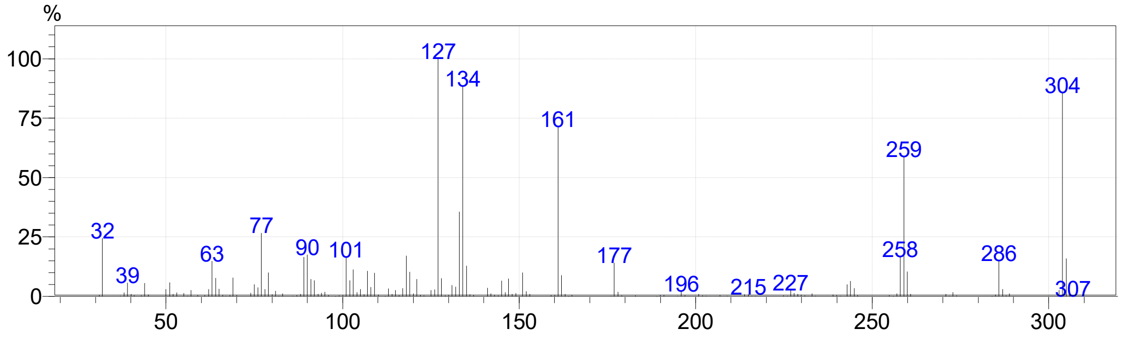

Figure 95 – Mass spectrum of compound **4i**.

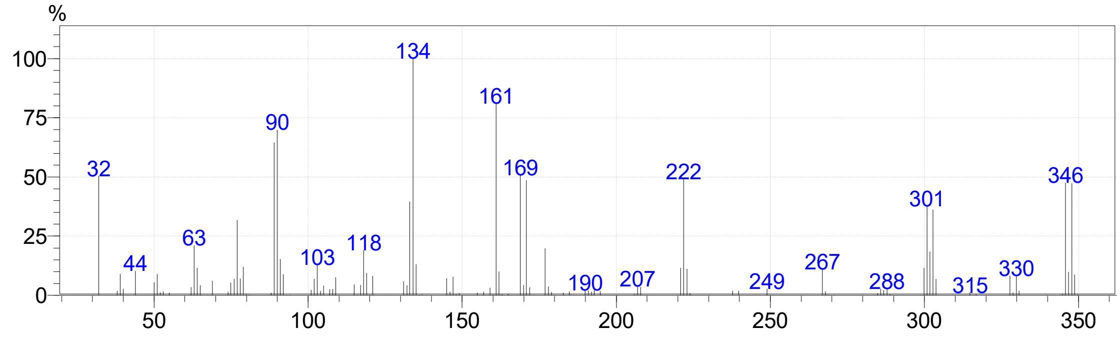

Figure 96 – Mass spectrum of compound **4j**.

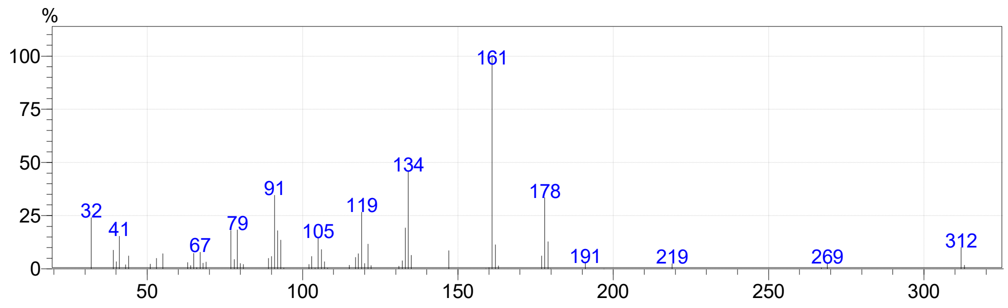

Figure 97 – Mass spectrum of compound **4k**.

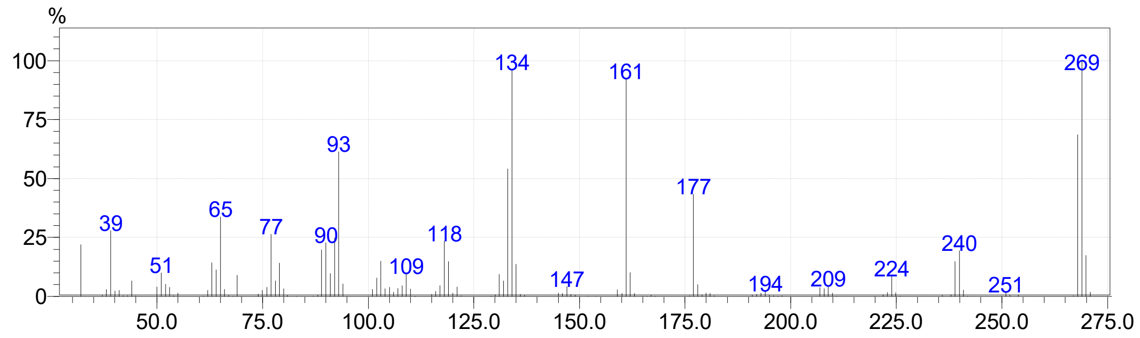

Figure 98 – Mass spectrum of compound **4l**.

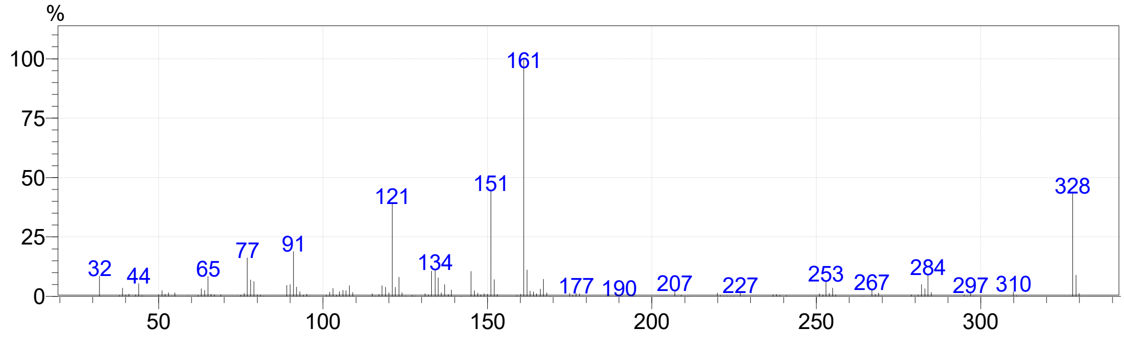

Figure S99 – Mass spectrum of compound **4m**.

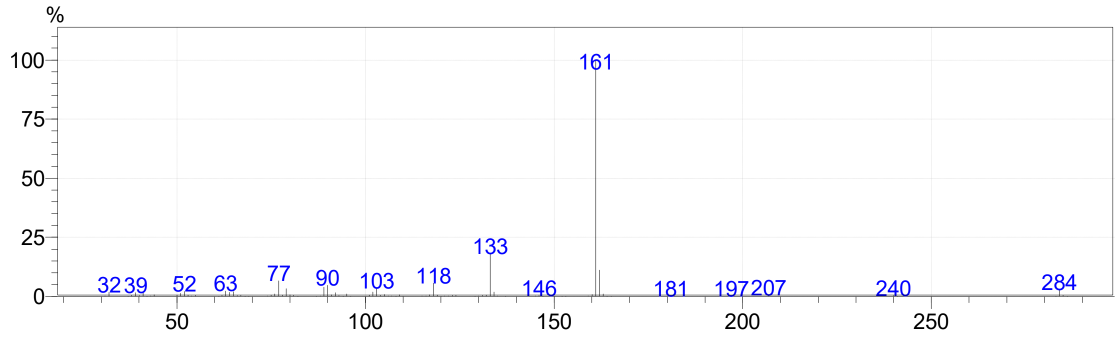

Figure S100 – Mass spectrum of compound **4n**.

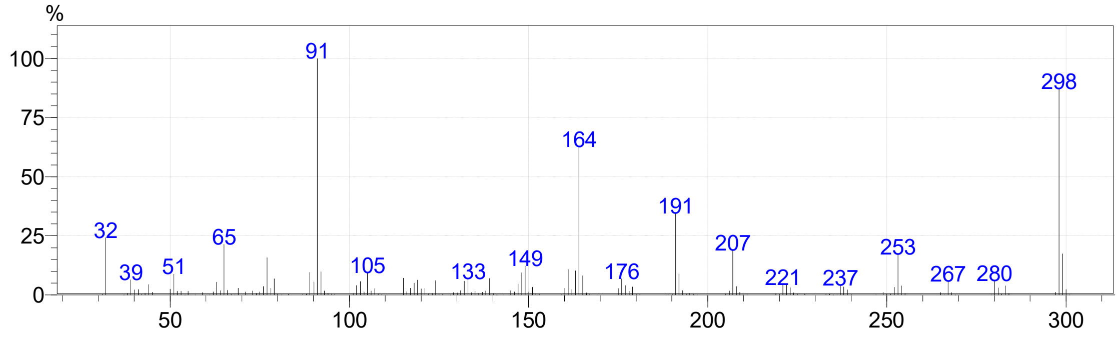

Figure S101 – Mass spectrum of compound **5a**.

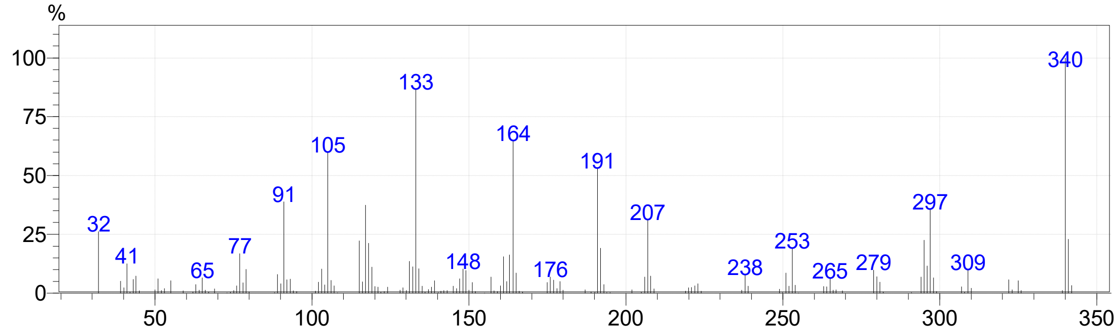

Figure S102 – Mass spectrum of compound **5b**.

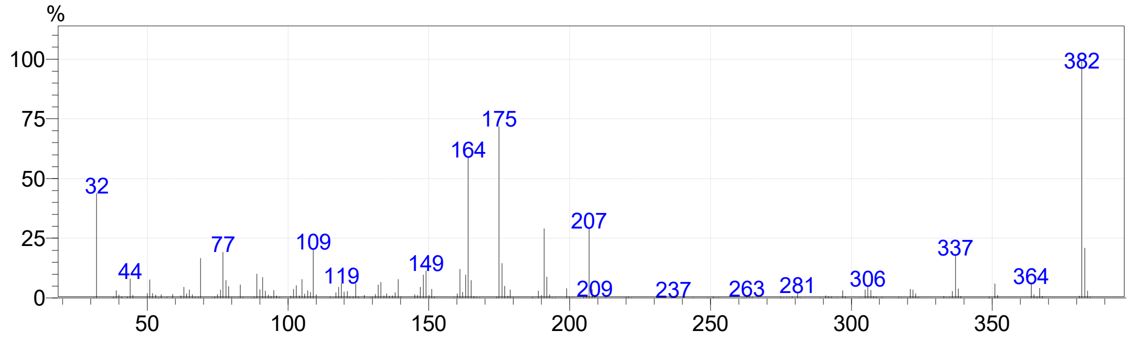

Figure S103 – Mass spectrum of compound **5d**.

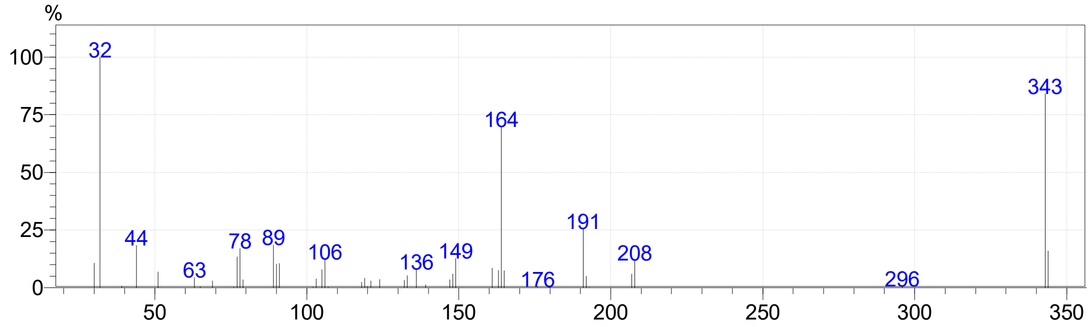

Figure S104 – Mass spectrum of compound **5e**.

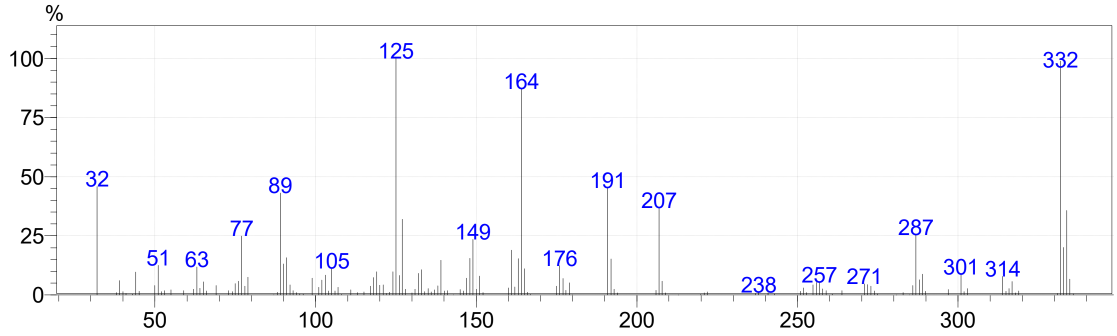

Figure S105 – Mass spectrum of compound **5f**.

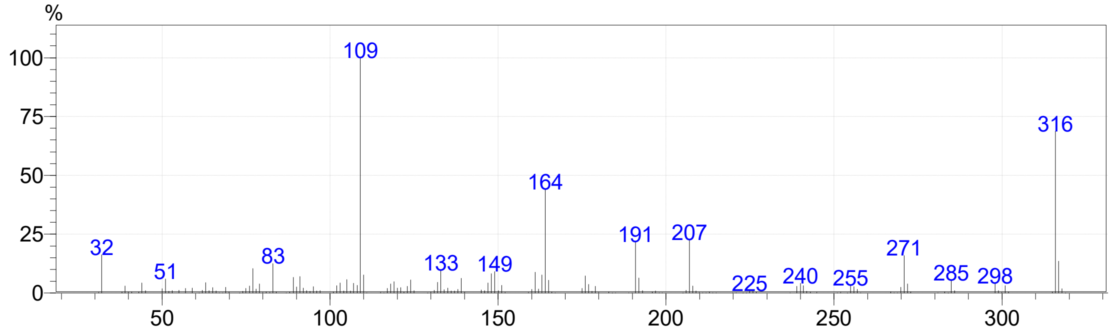

Figure S106 – Mass spectrum of compound **5h**.

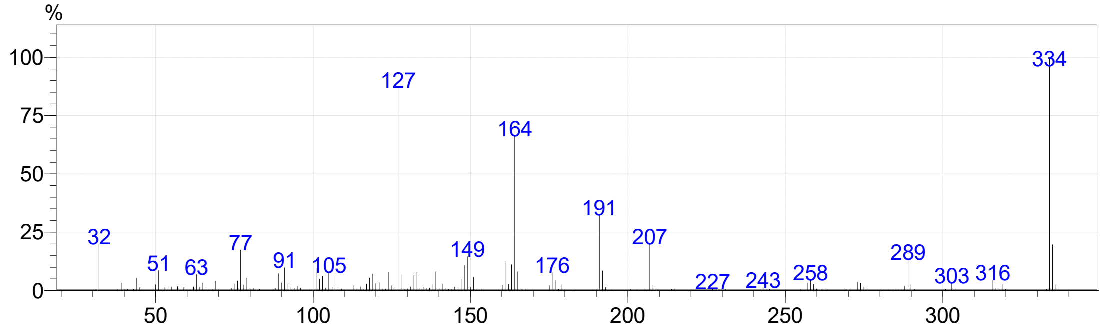

Figure S107 – Mass spectrum of compound **5i**.

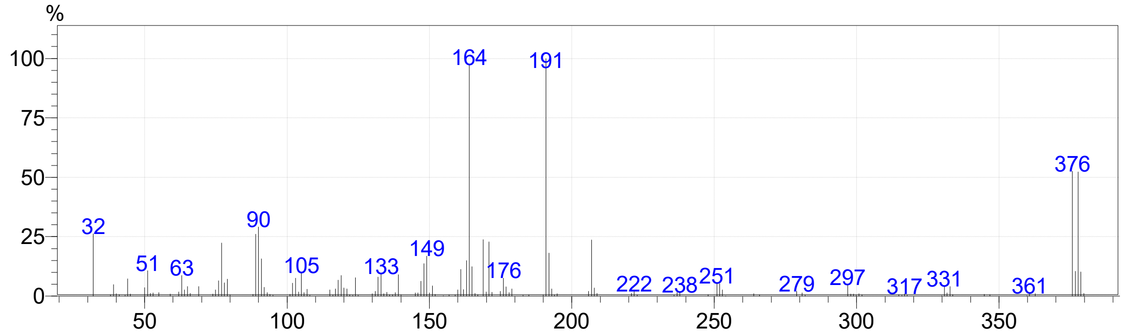

Figure S108 – Mass spectrum of compound **5j**.

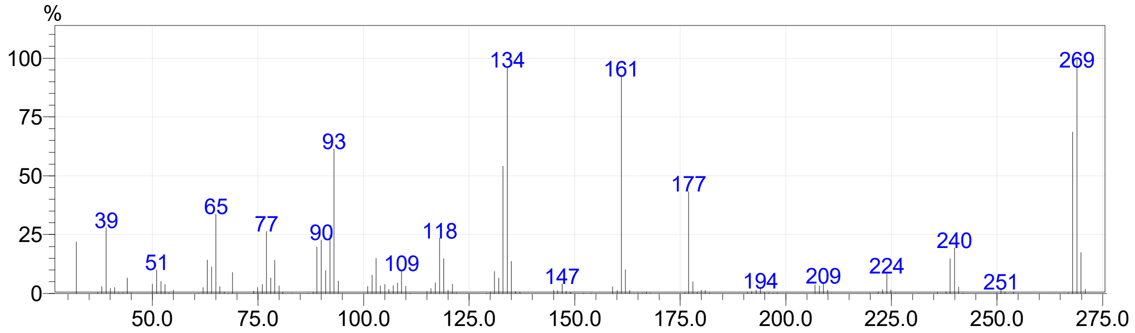

Figure S109 – Mass spectrum of compound **5l**.

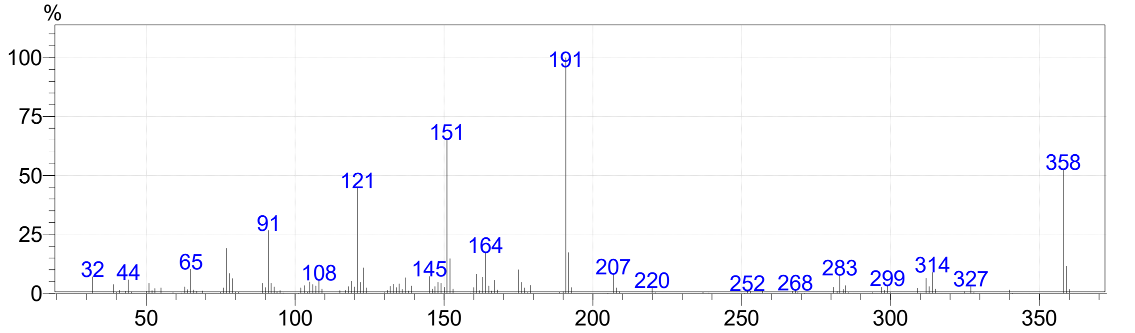

Figure S110 – Mass spectrum of compound **5m**.

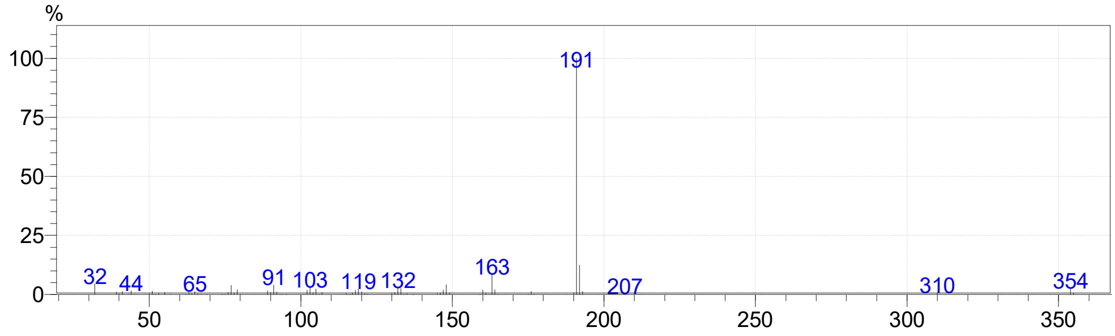

Figure S111 – Mass spectrum of compound **5n**.

CHARACTERIZATION OF COMPOUND (*E*)-2-METHOXYPHENYL 3-(3,4-DIMETHOXYPHENYL)ACRYLATE (5o) BY X-RAY DIFFRACTOMETRY

Room temperature (298(2) K) single-crystal X-ray diffraction data were measured on a Rigaku Oxford Diffraction 2020 X-ray diffractometer. The crystals were mounted on a goniostate and exposed to X-ray radiation (Mo Kα = 0.71073). The software used to collect data, structure solution, and refinements were the followings: CrysAlisPro 1.171.41.93a (Rigaku, OD, 2020) for collecting, refinements, and reduction of data; SHELST 2018/2 [48] and SHELXL 2018/3 [48] for structure solution and refinements; OLEX 2 1.5-beta [49] for structure analysis and molecular graphic construction. Nonhydrogen asymetric units were determined using electronic density distribution via Fourier transformation. A riding model was used for hydrogen postions with fixed valence bond lengths and angles. Nonhydrogen and hydrogen atoms were refined as anisotropic and isotropic ($U_{iso}(H) = 1.2U_{iso}(C_{CH})$ or $1.5U_{iso}(C_{CH3})$), respectively. Other collected parameters and the statistic refinements are shown in Table S1.

Tabela S1 – Crystallographic data and refinements for compound **5o**

Empirical formula	$C_{18}H_{18}O_5$
Formula weight	314.32
Crystaline sytem	Monoclinic
Spacial group	P 21/c
Z/Z′	4/1
T (K)/λ (Å)	293/0.71073
Unit cell dimensions	a = 9.40396(6) Å<br>b = 7.8454(5) Å<br>c = *22.2482(13)* Å<br>α = 90°<br>β = 101.934(6)°<br>γ = 90°
Volume of unit cell (Å^3)	1605.94(18)
Density – calculated (mg m^{-3})	1300
Absorption coefficient μ (mm^{-1})	0.095
2θ interval for data collection (°)	2.586 to 34.532
Intervals of index	h = -13-15<br>k = -11-12<br>l = -35-35
Collected reflections	26452
Independent reflections	6533
Observed reflections	3134
Symmetry factor	0.0395
Completeness to θ_{max} (%)	95.6
F(000)	664.0
Refined parameters	211
Goodness-of-fit on F^2	1.009
Residual index for I>2% (I) (R1)	0.0526
wR2 for all data	0.1689
Largest difference peak and hole (e/Å^3)	0.150 and -0.191
CCDC deposit number	2248204

The assymmetric unit of compound **5o** is depicted in Figure S112.

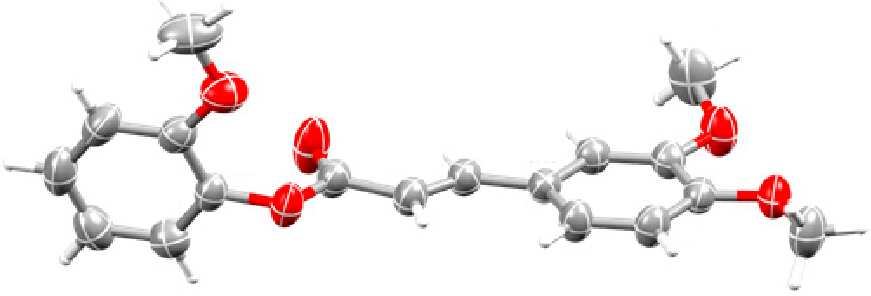

Figure S112 – Asymetric unit of compound **5o**. (50% probability ellipsoids for non hydrogen atoms, while hydrogens are arbitrary radius spheres)

The compound **5o** crystallizes in the P21/c monoclinic system, presenting only one molecule in the asymetric unit. The compound is essentially planar from the 3,4-dimethoxy phenyl group to the carbonyl ester functionaly, indication delocalized eletrons in this planar portion. However, the 2-methoxyphenyl group has its own plan. Moreover, the double bound presents a *trans* configuration, which agrees with theNMR data.

[48] Sheldrick, G. M. Crystal structure refinement with SHELXL. *Acta Crystallogr. Sect.* C **2015**, *71*, 3-8.

[49] Dolomanov, O.V.; Bourhis, L.J.; Gildea, R.J.; Howard, J. A. K.; Puschmann, H. OLEX2: A complete structure solution, refinement and analysis program. *J. Appl. Cristall.* **2009,** *42*, 339-341.
